# Power Aggregation Operators of Interval-Valued Atanassov-Intuitionistic Fuzzy Sets Based on Aczel–Alsina t-Norm and t-Conorm and Their Applications in Decision Making

**DOI:** 10.1007/s44196-023-00208-7

**Published:** 2023-03-28

**Authors:** Xinming Shi, Zeeshan Ali, Tahir Mahmood, Peide Liu

**Affiliations:** 1 School of Business Administration, Shandong Women’s University, Jinan, 250300 Shandong People’s Republic of China; 2 Department of Mathematics and Statistics, International Islamic University Islamabad, Islamabad, Pakistan; 3 Department of Mathematical Modeling, Statistics and Bioinformatics, KERMIT, Ghent University, Coupure Links 653, Ghent, Belgium

**Keywords:** Interval-valued Atanassov-intuitionistic fuzzy sets, Aczel–Alsina t-norm and t-conorm, Power aggregation operators, Decision-making techniques

## Abstract

Aczel–Alsina t-norm and t-conorm are important t-norm and t-conorm, and they are extended from algebraic t-norm and t-conorm. Obviously, Aczel–Alsina t-norm and t-conorm are more general than some existing t-norm and t-conorm. Furthermore, the power aggregation (PA) operator is also a very famous and valuable operator which can consider the power relation between any two input parameters. In addition, Interval-valued Atanassov-intuitionistic fuzzy set (IVA-IFS) can easily express uncertain information. In order to fully use their advantages, in this analysis, we extend the PA operators based on Aczel–Alsina t-norm and t-conorm to IVA-IFS and propose the interval-valued Atanassov-intuitionistic fuzzy Aczel–Alsina power averaging (IVA-IFAAPA), interval-valued Atanassov-intuitionistic fuzzy Aczel–Alsina power ordered averaging (IVA-IFAAPOA), interval-valued Atanassov-intuitionistic fuzzy Aczel–Alsina power geometric (IVA-IFAAPG) and interval-valued Atanassov-intuitionistic fuzzy Aczel–Alsina power ordered geometric (IVA-IFAAPOG) operators. Moreover, we discuss the properties of the presented operators such as idempotency, monotonicity, and boundedness. In addition, a multi-attribute decision-making (MADM) procedure is proposed to process the IVA-IF information. Finally, a practical example is used to show the effectiveness and superiority of the proposed method by comparing it with some existing operators.

## Introduction

The MADM is to select the best one or to give a ranking from the collection of a different and finite number of alternatives. In the traditional decision-making procedure, the attribute value can be only expressed by the crisp number, however, in real-life, many MADM problems cannot be described by real numbers because the attribute information is with ambiguity or uncertain. In order to process the ambiguity and complexity of the attributes, the FS [1] is presented which is an effective tool to express the uncertain information. Now, the FS theory has a lot of applications in various fields, for example, employment selection (doctors and nurses) during the COVID-19 pandemic [2], designing different clothes with the help of fuzzy superior mandelbrot set [3], business analysis and management research [4], data-driven modeling in manifolds based on the FS theory [5], environmental uncertainty and digital transformation for fuzzy information [6], N-soft set under the consideration of multi-agent preferences and their operators [7], the algebraic structure of multi-fuzzy soft information [8], multi q-fuzzy soft expert information [9], strategic decision-making analysis based on fuzzy N-soft expert information [10], and treatment decision of lung cancer with the help of fuzzy information [11].

Fuzzy set, soft set, and N-soft set are three different extensions, and the FS has received a lot of attention from various individuals, however, because there is only a membership grade in FS, in some cases, it is difficult to deal with some uncertain information. Some scholars pointed out that the negative or falsity or non-membership grade also played a very essential or critical role in some real-life problems, then, Atanassov [12] proposed a new structure called IFS which can deal with truth grade “$$\overline{\overline{{\zeta }_{\overline{\overline{{\mathord{\buildrel{\lower1pt\hbox{$\scriptscriptstyle\smile$}}\over{\mathfrak{W}}}}_{\mathfrak{L}}}}}}}\left(\widetilde{{\mu }_{\varrho }}\right)$$” and as well as falsity grade “$$\overline{\overline{{\eta }_{\overline{\overline{{\mathord{\buildrel{\lower1pt\hbox{$\scriptscriptstyle\smile$}}\over{\mathfrak{W}}}}_{\mathfrak{L}}}}}}}\left(\widetilde{{\mu }_{\varrho }}\right)$$” with a condition: $$0\le \overline{\overline{{\zeta }_{\overline{\overline{{\mathord{\buildrel{\lower1pt\hbox{$\scriptscriptstyle\smile$}}\over{\mathfrak{W}}}}_{\mathfrak{L}}}}}}}\left(\widetilde{{\mu }_{\varrho }}\right)+\overline{\overline{{\eta }_{\overline{\overline{{\mathord{\buildrel{\lower1pt\hbox{$\scriptscriptstyle\smile$}}\over{\mathfrak{W}}}}_{\mathfrak{L}}}}}}}\left(\widetilde{{\mu }_{\varrho }}\right)\le 1$$. Noticed that the FS is one of the valuable and famous cases when falsity grade $$\overline{\overline{{\eta }_{\overline{\overline{{\mathord{\buildrel{\lower1pt\hbox{$\scriptscriptstyle\smile$}}\over{\mathfrak{W}}}}_{\mathfrak{L}}}}}}}\left(\widetilde{{\mu }_{\varrho }}\right)=0$$ in the IFS. Furthermore, the IFS has been used in the fields of decision-making, artificial intelligence, machine learning, and clustering analysis, for example, IVIFS [13], stock prediction under the consideration of A-IF inferences [14], MADM technique for IFSs [15], distance measures for IFS and their application in various triangle centers [16], two novel types of measures in the IFSs [17], software bug triaging under the availability of IFSs and their application [18], the enhancement of color images under the uses of IFS [19], and positron emission tomography image segmentation for IFSs and their applications in clustering analysis [20].

Aggregating the collection of information into one is a very challenging task. Some scholars have proposed different types of operators such as arithmetic and geometric AOs. Further, Yager [21] developed the PA operator which is the generalized form of the above two different operators. Now the averaging, geometric, and PA operators are computed based on algebraic t-norm and t-conorm. Further, Aczel and Alsina [22] proposed Aczel–Alsina t-norm and t-conorm which are the modified and generalized forms of algebraic norms. The Aczel–Alsina t-norm and t-conorm are the generalization of the algebraic t-norm and t-conorm, so it is clear that the aggregation operators based on algebraic t-norm and t-conorm are the special cases of the proposed operators. Then they received a lot of attention from different scholars because of their remarkable features. For instance, Xu [23] proposed the averaging AOs for IFS, Xu and Yager [24] proposed the geometric AOs for IFS, Jiang et al. [25] and Xu [26] proposed the PA operators for IFS, Senapati et al. [27] and Senapati et al. [28] proposed the Aczel–Alsina averaging AOs for IFS and the geometric Aczel–Alsina AOs for IFS. Senapati et al. [29] proposed the weighted PA operators based on Aczel–Alsina operational laws. Furthermore, geometric AOs for IVIFS was discovered by Wei and Wang [30]. Wang et al. [31] also examined the theory of averaging AOs for IVIFSs. Aczel–Alsina AOs for IVIFS was invented by Senapati et al. [32]. Where the power AOs for IVIFS was derived by He et al. [33]. Therefore, we concluded that the proposed operators from Xu [23], Xu and Yager [24], Senapati et al. [32] and He et al. [33] are the special cases of proposed studies. Furthermore, we discuss the advantages of the proposed works, such as

1. If we have only used the theory of PA operators based on algebraic laws, then the derived theory will be changed for the proposed theory of He et al. [33].
2. If we have only used the theory of simple averaging and geometric aggregation operators based on Aczel–Alsina operational laws, then the derived theory will be changed for the proposed theory of Senapati et al. [32].
3. If we have only used the theory of simple averaging and geometric aggregation operators based on algebraic operational laws, then the derived theory will be changed for the proposed theory of Wei and Wang [30] and Wang et al. [31].

From the above analysis, it is clear that some scholars studied the power aggregation operators based on different types of norms, Senapati et al. [29] proposed the weighted PA operators based on Aczel–Alsina operational laws and they thought that the monotonicity is also kept for the proposed work, but in actuality, they are not held. In this manuscript, we aim to propose the PA operators based on Aczel–Alsina operational laws for IVA-IFSs. Since the PA operators based on Aczel–Alsina t-norm and t-conorm are more general than some existing averaging and geometric AOs, and IVA-IFS can easily express the uncertain information, it is necessary to develop some new AOs for IVA-IFS based on Aczel–Alsina t-norm and t-conorm. In this scenario, our main target or contributions are shown as follows.

1. To propose the IVA-IFAAPA, IVA-IFAAPOA, IVA-IFAAPG, and IVA-IFAAPOG operators.
2. To discuss the properties of the presented operators.
3. To develop a MADM method with IVA-IF information based on the proposed operators.
4. To demonstrate the advantages of the proposed method by comparing it with various prevailing operators by some examples.

This article is summarized as follows: in Sect. 2, we review the PA operators, Aczel–Alsina operational laws for IVA-IFSs, and the comparison method for two IVA-IFSs. In Sect. 3, we develop the IVA-IFAAPA, IVA-IFAAPOA, IVA-IFAAPG, and IVA-IFAAPOG operators, and discuss the properties of the presented operators such as idempotency, monotonicity, and boundedness. In Sect. 4, a procedure for decision-making is developed for the MADM problem with A-IF information. In Sect. 5, a practical example is used to show the advantages of the proposed technique by comparing it with various prevailing operators. Some valuable conclusions and future research are stated in Sect. 6.

## Preliminaries

In this section, we reviewed the PA operators, IVA-IFSs, and Aczel–Alsina operational laws.

### Definition 1

[21] For the finite positive numbers $$\overline{\overline{{\mathord{\buildrel{\lower1pt\hbox{$\scriptscriptstyle\smile$}}\over{\mathfrak{W}}}}_{{\mathfrak{L}}_{\tau }}}},\tau =\mathrm{1,2},\dots ,{\rm Z}$$, then the PA operator is derived by

1 $$PA\left(\overline{\overline{{\mathord{\buildrel{\lower1pt\hbox{$\scriptscriptstyle\smile$}}\over{\mathfrak{W}}}}_{{\mathfrak{L}}_{1}}}},\overline{\overline{{\mathord{\buildrel{\lower1pt\hbox{$\scriptscriptstyle\smile$}}\over{\mathfrak{W}}}}_{{\mathfrak{L}}_{2}}}},\dots ,\overline{\overline{{\mathord{\buildrel{\lower1pt\hbox{$\scriptscriptstyle\smile$}}\over{\mathfrak{W}}}}_{{\mathfrak{L}}_{\rm Z}}}}\right)=\frac{\sum_{\tau =1}^{\rm Z}\left(1+\overline{\overline{\mathfrak{I}}}\left(\overline{\overline{{\mathord{\buildrel{\lower1pt\hbox{$\scriptscriptstyle\smile$}}\over{\mathfrak{W}}}}_{{\mathfrak{L}}_{\tau }}}}\right)\right)\overline{\overline{{\mathord{\buildrel{\lower1pt\hbox{$\scriptscriptstyle\smile$}}\over{\mathfrak{W}}}}_{{\mathfrak{L}}_{\tau }}}}}{\sum_{\tau =1}^{\rm Z}\left(1+\overline{\overline{\mathfrak{I}}}\left(\overline{\overline{{\mathord{\buildrel{\lower1pt\hbox{$\scriptscriptstyle\smile$}}\over{\mathfrak{W}}}}_{{\mathfrak{L}}_{\tau }}}}\right)\right)}$$

Noticed that $$\overline{\overline{\mathfrak{I}}}\left(\overline{\overline{{\mathord{\buildrel{\lower1pt\hbox{$\scriptscriptstyle\smile$}}\over{\mathfrak{W}}}}_{{\mathfrak{L}}_{\tau }}}}\right)=\sum_{\begin{array}{c}s=1,\\ s\ne \tau \end{array}}^{\rm Z}Sup\left(\overline{\overline{{\mathord{\buildrel{\lower1pt\hbox{$\scriptscriptstyle\smile$}}\over{\mathfrak{W}}}}_{{\mathfrak{L}}_{\tau }}}},\overline{\overline{{\mathord{\buildrel{\lower1pt\hbox{$\scriptscriptstyle\smile$}}\over{\mathfrak{W}}}}_{{\mathfrak{L}}_{k}}}}\right)$$, which expresses the relationship between $$\overline{\overline{{\mathord{\buildrel{\lower1pt\hbox{$\scriptscriptstyle\smile$}}\over{\mathfrak{W}}}}_{{\mathfrak{L}}_{\tau }}}}$$ and $$\overline{\overline{{\mathord{\buildrel{\lower1pt\hbox{$\scriptscriptstyle\smile$}}\over{\mathfrak{W}}}}_{{\mathfrak{L}}_{k}}}}$$ with various characteristics:

1. $$Sup\left(\overline{\overline{{\mathord{\buildrel{\lower1pt\hbox{$\scriptscriptstyle\smile$}}\over{\mathfrak{W}}}}_{{\mathfrak{L}}_{\tau }}}},\overline{\overline{{\mathord{\buildrel{\lower1pt\hbox{$\scriptscriptstyle\smile$}}\over{\mathfrak{W}}}}_{{\mathfrak{L}}_{k}}}}\right)\in \left[\mathrm{0,1}\right].$$
2. $$Sup\left(\overline{\overline{{\mathord{\buildrel{\lower1pt\hbox{$\scriptscriptstyle\smile$}}\over{\mathfrak{W}}}}_{{\mathfrak{L}}_{\tau }}}},\overline{\overline{{\mathord{\buildrel{\lower1pt\hbox{$\scriptscriptstyle\smile$}}\over{\mathfrak{W}}}}_{{\mathfrak{L}}_{k}}}}\right)=Sup\left(\overline{\overline{{\mathord{\buildrel{\lower1pt\hbox{$\scriptscriptstyle\smile$}}\over{\mathfrak{W}}}}_{{\mathfrak{L}}_{k}}}},\overline{\overline{{\mathord{\buildrel{\lower1pt\hbox{$\scriptscriptstyle\smile$}}\over{\mathfrak{W}}}}_{{\mathfrak{L}}_{\tau }}}}\right).$$
3. $$Sup\left(\overline{\overline{{\mathord{\buildrel{\lower1pt\hbox{$\scriptscriptstyle\smile$}}\over{\mathfrak{W}}}}_{{\mathfrak{L}}_{\tau }}}},\overline{\overline{{\mathord{\buildrel{\lower1pt\hbox{$\scriptscriptstyle\smile$}}\over{\mathfrak{W}}}}_{{\mathfrak{L}}_{k}}}}\right)\ge Sup\left({\overline{\overline{{\mathord{\buildrel{\lower1pt\hbox{$\scriptscriptstyle\smile$}}\over{\mathfrak{W}}}}_{{\mathfrak{L}}_{\tau }}}}}^{*},{\overline{\overline{{\mathord{\buildrel{\lower1pt\hbox{$\scriptscriptstyle\smile$}}\over{\mathfrak{W}}}}_{{\mathfrak{L}}_{k}}}}}^{*}\right)$$ when $$\left|\overline{\overline{{\mathord{\buildrel{\lower1pt\hbox{$\scriptscriptstyle\smile$}}\over{\mathfrak{W}}}}_{{\mathfrak{L}}_{\tau }}}}-\overline{\overline{{\mathord{\buildrel{\lower1pt\hbox{$\scriptscriptstyle\smile$}}\over{\mathfrak{W}}}}_{{\mathfrak{L}}_{k}}}}\right|\le \left|{\overline{\overline{{\mathord{\buildrel{\lower1pt\hbox{$\scriptscriptstyle\smile$}}\over{\mathfrak{W}}}}_{{\mathfrak{L}}_{\tau }}}}}^{*}-{\overline{\overline{{\mathord{\buildrel{\lower1pt\hbox{$\scriptscriptstyle\smile$}}\over{\mathfrak{W}}}}_{{\mathfrak{L}}_{k}}}}}^{*}\right|.$$

### Definition 2

[13] For the universal set $\widetilde{{\mathbb{X}}_{U}}$, the IVA-IF set was given such that

2 $$\overline{\overline{{\mathord{\buildrel{\lower1pt\hbox{$\scriptscriptstyle\smile$}}\over{\mathfrak{W}}}}_{\mathfrak{L}}}}=\left\{\left(\overline{\overline{{\zeta }_{\overline{\overline{{\mathord{\buildrel{\lower1pt\hbox{$\scriptscriptstyle\smile$}}\over{\mathfrak{W}}}}_{\mathfrak{L}}}}}}}\left(\widetilde{{\mu }_{\varrho }}\right),\overline{\overline{{\eta }_{\overline{\overline{{\mathord{\buildrel{\lower1pt\hbox{$\scriptscriptstyle\smile$}}\over{\mathfrak{W}}}}_{\mathfrak{L}}}}}}}\left(\widetilde{{\mu }_{\varrho }}\right)\right):\widetilde{{\mu }_{\varrho }}\in \widetilde{{\mathbb{X}}_{U}}\right\}$$

Here, $$\overline{\overline{{\zeta }_{\overline{\overline{{\mathord{\buildrel{\lower1pt\hbox{$\scriptscriptstyle\smile$}}\over{\mathfrak{W}}}}_{\mathfrak{L}}}}}}}\left(\widetilde{{\mu }_{\varrho }}\right)=\left[{\overline{\overline{{\zeta }_{\overline{\overline{{\mathord{\buildrel{\lower1pt\hbox{$\scriptscriptstyle\smile$}}\over{\mathfrak{W}}}}_{\mathfrak{L}}}}}}}}^{l}\left(\widetilde{{\mu }_{\varrho }}\right),{\overline{\overline{{\zeta }_{\overline{\overline{{\mathord{\buildrel{\lower1pt\hbox{$\scriptscriptstyle\smile$}}\over{\mathfrak{W}}}}_{\mathfrak{L}}}}}}}}^{u}\left(\widetilde{{\mu }_{\varrho }}\right)\right]$$ and $$\overline{\overline{{\eta }_{\overline{\overline{{\mathord{\buildrel{\lower1pt\hbox{$\scriptscriptstyle\smile$}}\over{\mathfrak{W}}}}_{\mathfrak{L}}}}}}}\left(\widetilde{{\mu }_{\varrho }}\right)=\left[{\overline{\overline{{\eta }_{\overline{\overline{{\mathord{\buildrel{\lower1pt\hbox{$\scriptscriptstyle\smile$}}\over{\mathfrak{W}}}}_{\mathfrak{L}}}}}}}}^{l}\left(\widetilde{{\mu }_{\varrho }}\right),{\overline{\overline{{\eta }_{\overline{\overline{{\mathord{\buildrel{\lower1pt\hbox{$\scriptscriptstyle\smile$}}\over{\mathfrak{W}}}}_{\mathfrak{L}}}}}}}}^{u}\left(\widetilde{{\mu }_{\varrho }}\right)\right]$$ represents the values of truth and falsity and meets $$0\le {\overline{\overline{{\zeta }_{\overline{\overline{{\mathord{\buildrel{\lower1pt\hbox{$\scriptscriptstyle\smile$}}\over{\mathfrak{W}}}}_{\mathfrak{L}}}}}}}}^{u}\left(\widetilde{{\mu }_{\varrho }}\right)+{\overline{\overline{{\eta }_{\overline{\overline{{\mathord{\buildrel{\lower1pt\hbox{$\scriptscriptstyle\smile$}}\over{\mathfrak{W}}}}_{\mathfrak{L}}}}}}}}^{u}\left(\widetilde{{\mu }_{\varrho }}\right)\le 1$$, then a neutral grade is expressed by $$\overline{\overline{{\mathfrak{R}}_{\overline{\overline{{\mathord{\buildrel{\lower1pt\hbox{$\scriptscriptstyle\smile$}}\over{\mathfrak{W}}}}_{\mathfrak{L}}}}}}}\left(\widetilde{{\mu }_{\varrho }}\right)=\left[{\overline{\overline{{\mathfrak{R}}_{\overline{\overline{{\mathord{\buildrel{\lower1pt\hbox{$\scriptscriptstyle\smile$}}\over{\mathfrak{W}}}}_{\mathfrak{L}}}}}}}}^{l}\left(\widetilde{{\mu }_{\varrho }}\right),{\overline{\overline{{\mathfrak{R}}_{\overline{\overline{{\mathord{\buildrel{\lower1pt\hbox{$\scriptscriptstyle\smile$}}\over{\mathfrak{W}}}}_{\mathfrak{L}}}}}}}}^{u}\left(\widetilde{{\mu }_{\varrho }}\right)\right]=\left[\left(1-\left({\overline{\overline{{\zeta }_{\overline{\overline{{\mathord{\buildrel{\lower1pt\hbox{$\scriptscriptstyle\smile$}}\over{\mathfrak{W}}}}_{\mathfrak{L}}}}}}}}^{u}\left(\widetilde{{\mu }_{\varrho }}\right)+{\overline{\overline{{\eta }_{\overline{\overline{{\mathord{\buildrel{\lower1pt\hbox{$\scriptscriptstyle\smile$}}\over{\mathfrak{W}}}}_{\mathfrak{L}}}}}}}}^{u}\left(\widetilde{{\mu }_{\varrho }}\right)\right)\right),\left(1-\left({\overline{\overline{{\zeta }_{\overline{\overline{{\mathord{\buildrel{\lower1pt\hbox{$\scriptscriptstyle\smile$}}\over{\mathfrak{W}}}}_{\mathfrak{L}}}}}}}}^{l}\left(\widetilde{{\mu }_{\varrho }}\right)+{\overline{\overline{{\eta }_{\overline{\overline{{\mathord{\buildrel{\lower1pt\hbox{$\scriptscriptstyle\smile$}}\over{\mathfrak{W}}}}_{\mathfrak{L}}}}}}}}^{l}\left(\widetilde{{\mu }_{\varrho }}\right)\right)\right)\right]$$. Furthermore, the simple form of IVA-IFN is expressed by $$\overline{\overline{{\mathord{\buildrel{\lower1pt\hbox{$\scriptscriptstyle\smile$}}\over{\mathfrak{W}}}}_{{\mathfrak{L}}_{\tau }}}}=\left(\left[{\overline{\overline{{\zeta }_{\overline{\overline{{\mathord{\buildrel{\lower1pt\hbox{$\scriptscriptstyle\smile$}}\over{\mathfrak{W}}}}_{{\mathfrak{L}}_{\tau }}}}}}}}^{l},{\overline{\overline{{\zeta }_{\overline{\overline{{\mathord{\buildrel{\lower1pt\hbox{$\scriptscriptstyle\smile$}}\over{\mathfrak{W}}}}_{{\mathfrak{L}}_{\tau }}}}}}}}^{u}\right],\left[{\overline{\overline{{\eta }_{\overline{\overline{{\mathord{\buildrel{\lower1pt\hbox{$\scriptscriptstyle\smile$}}\over{\mathfrak{W}}}}_{{\mathfrak{L}}_{\tau }}}}}}}}^{l},{\overline{\overline{{\eta }_{\overline{\overline{{\mathord{\buildrel{\lower1pt\hbox{$\scriptscriptstyle\smile$}}\over{\mathfrak{W}}}}_{{\mathfrak{L}}_{\tau }}}}}}}}^{u}\right]\right),\tau =\mathrm{1,2},\dots ,{\rm Z}$$.

### Definition 3

For two IVA-IFNs $$\overline{\overline{{\mathord{\buildrel{\lower1pt\hbox{$\scriptscriptstyle\smile$}}\over{\mathfrak{W}}}}_{{\mathfrak{L}}_{\tau }}}}=\left(\left[{\overline{\overline{{\zeta }_{\overline{\overline{{\mathord{\buildrel{\lower1pt\hbox{$\scriptscriptstyle\smile$}}\over{\mathfrak{W}}}}_{{\mathfrak{L}}_{\tau }}}}}}}}^{l},{\overline{\overline{{\zeta }_{\overline{\overline{{\mathord{\buildrel{\lower1pt\hbox{$\scriptscriptstyle\smile$}}\over{\mathfrak{W}}}}_{{\mathfrak{L}}_{\tau }}}}}}}}^{u}\right],\left[{\overline{\overline{{\eta }_{\overline{\overline{{\mathord{\buildrel{\lower1pt\hbox{$\scriptscriptstyle\smile$}}\over{\mathfrak{W}}}}_{{\mathfrak{L}}_{\tau }}}}}}}}^{l},{\overline{\overline{{\eta }_{\overline{\overline{{\mathord{\buildrel{\lower1pt\hbox{$\scriptscriptstyle\smile$}}\over{\mathfrak{W}}}}_{{\mathfrak{L}}_{\tau }}}}}}}}^{u}\right]\right),\tau =\mathrm{1,2}$$, then the Aczel–Alsina operational laws are shown as

3 $$\overline{\overline{{\mathord{\buildrel{\lower1pt\hbox{$\scriptscriptstyle\smile$}}\over{\mathfrak{W}}}}_{{\mathfrak{L}}_{1}}}}\oplus \overline{\overline{{\mathord{\buildrel{\lower1pt\hbox{$\scriptscriptstyle\smile$}}\over{\mathfrak{W}}}}_{{\mathfrak{L}}_{2}}}}=\left(\begin{array}{c}\left[1-{e}^{-{\left({\left(-\mathrm{log}\left(1-{\overline{\overline{{\zeta }_{\overline{\overline{{\mathord{\buildrel{\lower1pt\hbox{$\scriptscriptstyle\smile$}}\over{\mathfrak{W}}}}_{{\mathfrak{L}}_{1}}}}}}}}^{l}\right)\right)}^{\Lambda }+{\left(-\mathrm{log}\left(1-{\overline{\overline{{\zeta }_{\overline{\overline{{\mathord{\buildrel{\lower1pt\hbox{$\scriptscriptstyle\smile$}}\over{\mathfrak{W}}}}_{{\mathfrak{L}}_{2}}}}}}}}^{l}\right)\right)}^{\Lambda }\right)}^{\frac{1}{\Lambda }}},1-{e}^{-{\left({\left(-\mathrm{log}\left(1-{\overline{\overline{{\zeta }_{\overline{\overline{{\mathord{\buildrel{\lower1pt\hbox{$\scriptscriptstyle\smile$}}\over{\mathfrak{W}}}}_{{\mathfrak{L}}_{1}}}}}}}}^{u}\right)\right)}^{\Lambda }+{\left(-\mathrm{log}\left(1-{\overline{\overline{{\zeta }_{\overline{\overline{{\mathord{\buildrel{\lower1pt\hbox{$\scriptscriptstyle\smile$}}\over{\mathfrak{W}}}}_{{\mathfrak{L}}_{2}}}}}}}}^{u}\right)\right)}^{\Lambda }\right)}^{\frac{1}{\Lambda }}}\right],\\ \left[{e}^{-{\left({\left(-\mathrm{log}\left({\overline{\overline{{\eta }_{\overline{\overline{{\mathord{\buildrel{\lower1pt\hbox{$\scriptscriptstyle\smile$}}\over{\mathfrak{W}}}}_{{\mathfrak{L}}_{1}}}}}}}}^{l}\right)\right)}^{\Lambda }+{\left(-\mathrm{log}\left({\overline{\overline{{\eta }_{\overline{\overline{{\mathord{\buildrel{\lower1pt\hbox{$\scriptscriptstyle\smile$}}\over{\mathfrak{W}}}}_{{\mathfrak{L}}_{2}}}}}}}}^{l}\right)\right)}^{\Lambda }\right)}^{\frac{1}{\Lambda }}},{e}^{-{\left({\left(-\mathrm{log}\left({\overline{\overline{{\eta }_{\overline{\overline{{\mathord{\buildrel{\lower1pt\hbox{$\scriptscriptstyle\smile$}}\over{\mathfrak{W}}}}_{{\mathfrak{L}}_{1}}}}}}}}^{u}\right)\right)}^{\Lambda }+{\left(-\mathrm{log}\left({\overline{\overline{{\eta }_{\overline{\overline{{\mathord{\buildrel{\lower1pt\hbox{$\scriptscriptstyle\smile$}}\over{\mathfrak{W}}}}_{{\mathfrak{L}}_{2}}}}}}}}^{u}\right)\right)}^{\Lambda }\right)}^{\frac{1}{\Lambda }}}\right]\end{array}\right)$$

4 $$\overline{\overline{{\mathord{\buildrel{\lower1pt\hbox{$\scriptscriptstyle\smile$}}\over{\mathfrak{W}}}}_{{\mathfrak{L}}_{1}}}}\otimes \overline{\overline{{\mathord{\buildrel{\lower1pt\hbox{$\scriptscriptstyle\smile$}}\over{\mathfrak{W}}}}_{{\mathfrak{L}}_{2}}}}=\left(\begin{array}{c}\left[{e}^{-{\left({\left(-\mathrm{log}\left({\overline{\overline{{\zeta }_{\overline{\overline{{\mathord{\buildrel{\lower1pt\hbox{$\scriptscriptstyle\smile$}}\over{\mathfrak{W}}}}_{{\mathfrak{L}}_{1}}}}}}}}^{l}\right)\right)}^{\Lambda }+{\left(-\mathrm{log}\left({\overline{\overline{{\zeta }_{\overline{\overline{{\mathord{\buildrel{\lower1pt\hbox{$\scriptscriptstyle\smile$}}\over{\mathfrak{W}}}}_{{\mathfrak{L}}_{2}}}}}}}}^{l}\right)\right)}^{\Lambda }\right)}^{\frac{1}{\Lambda }}},{e}^{-{\left({\left(-\mathrm{log}\left({\overline{\overline{{\zeta }_{\overline{\overline{{\mathord{\buildrel{\lower1pt\hbox{$\scriptscriptstyle\smile$}}\over{\mathfrak{W}}}}_{{\mathfrak{L}}_{1}}}}}}}}^{u}\right)\right)}^{\Lambda }+{\left(-\mathrm{log}\left({\overline{\overline{{\zeta }_{\overline{\overline{{\mathord{\buildrel{\lower1pt\hbox{$\scriptscriptstyle\smile$}}\over{\mathfrak{W}}}}_{{\mathfrak{L}}_{2}}}}}}}}^{u}\right)\right)}^{\Lambda }\right)}^{\frac{1}{\Lambda }}}\right],\\ \left[1-{e}^{-{\left({\left(-\mathrm{log}\left(1-{\overline{\overline{{\eta }_{\overline{\overline{{\mathord{\buildrel{\lower1pt\hbox{$\scriptscriptstyle\smile$}}\over{\mathfrak{W}}}}_{{\mathfrak{L}}_{1}}}}}}}}^{l}\right)\right)}^{\Lambda }+{\left(-\mathrm{log}\left(1-{\overline{\overline{{\eta }_{\overline{\overline{{\mathord{\buildrel{\lower1pt\hbox{$\scriptscriptstyle\smile$}}\over{\mathfrak{W}}}}_{{\mathfrak{L}}_{2}}}}}}}}^{l}\right)\right)}^{\Lambda }\right)}^{\frac{1}{\Lambda }}},1-{e}^{-{\left({\left(-\mathrm{log}\left(1-{\overline{\overline{{\eta }_{\overline{\overline{{\mathord{\buildrel{\lower1pt\hbox{$\scriptscriptstyle\smile$}}\over{\mathfrak{W}}}}_{{\mathfrak{L}}_{1}}}}}}}}^{u}\right)\right)}^{\Lambda }+{\left(-\mathrm{log}\left(1-{\overline{\overline{{\eta }_{\overline{\overline{{\mathord{\buildrel{\lower1pt\hbox{$\scriptscriptstyle\smile$}}\over{\mathfrak{W}}}}_{{\mathfrak{L}}_{2}}}}}}}}^{u}\right)\right)}^{\Lambda }\right)}^{\frac{1}{\Lambda }}}\right]\end{array}\right)$$

5 $$\overline{\overline{{\theta }_{S}}}\overline{\overline{{\mathord{\buildrel{\lower1pt\hbox{$\scriptscriptstyle\smile$}}\over{\mathfrak{W}}}}_{{\mathfrak{L}}_{1}}}}=\left(\begin{array}{c}\left[1-{e}^{-{\left(\overline{\overline{{\theta }_{S}}}{\left(-\mathrm{log}\left(1-{\overline{\overline{{\zeta }_{\overline{\overline{{\mathord{\buildrel{\lower1pt\hbox{$\scriptscriptstyle\smile$}}\over{\mathfrak{W}}}}_{{\mathfrak{L}}_{1}}}}}}}}^{l}\right)\right)}^{\Lambda }\right)}^{\frac{1}{\Lambda }}},1-{e}^{-{\left(\overline{\overline{{\theta }_{S}}}{\left(-\mathrm{log}\left(1-{\overline{\overline{{\zeta }_{\overline{\overline{{\mathord{\buildrel{\lower1pt\hbox{$\scriptscriptstyle\smile$}}\over{\mathfrak{W}}}}_{{\mathfrak{L}}_{1}}}}}}}}^{u}\right)\right)}^{\Lambda }\right)}^{\frac{1}{\Lambda }}}\right],\\ \left[{e}^{-{\left(\overline{\overline{{\theta }_{S}}}{\left(-\mathrm{log}\left({\overline{\overline{{\eta }_{\overline{\overline{{\mathord{\buildrel{\lower1pt\hbox{$\scriptscriptstyle\smile$}}\over{\mathfrak{W}}}}_{{\mathfrak{L}}_{1}}}}}}}}^{l}\right)\right)}^{\Lambda }\right)}^{\frac{1}{\Lambda }}},{e}^{-{\left(\overline{\overline{{\theta }_{S}}}{\left(-\mathrm{log}\left({\overline{\overline{{\eta }_{\overline{\overline{{\mathord{\buildrel{\lower1pt\hbox{$\scriptscriptstyle\smile$}}\over{\mathfrak{W}}}}_{{\mathfrak{L}}_{1}}}}}}}}^{u}\right)\right)}^{\Lambda }\right)}^{\frac{1}{\Lambda }}}\right]\end{array}\right)$$

6 $${\overline{\overline{{\mathord{\buildrel{\lower1pt\hbox{$\scriptscriptstyle\smile$}}\over{\mathfrak{W}}}}_{{\mathfrak{L}}_{1}}}}}^{\overline{\overline{{\theta }_{S}}}}=\left(\begin{array}{c}\left[{e}^{-{\left(\overline{\overline{{\theta }_{S}}}{\left(-\mathrm{log}\left({\overline{\overline{{\zeta }_{\overline{\overline{{\mathord{\buildrel{\lower1pt\hbox{$\scriptscriptstyle\smile$}}\over{\mathfrak{W}}}}_{{\mathfrak{L}}_{1}}}}}}}}^{l}\right)\right)}^{\Lambda }\right)}^{\frac{1}{\Lambda }}},{e}^{-{\left(\overline{\overline{{\theta }_{S}}}{\left(-\mathrm{log}\left({\overline{\overline{{\zeta }_{\overline{\overline{{\mathord{\buildrel{\lower1pt\hbox{$\scriptscriptstyle\smile$}}\over{\mathfrak{W}}}}_{{\mathfrak{L}}_{1}}}}}}}}^{u}\right)\right)}^{\Lambda }\right)}^{\frac{1}{\Lambda }}}\right],\\ \left[1-{e}^{-{\left(\overline{\overline{{\theta }_{S}}}{\left(-\mathrm{log}\left(1-{\overline{\overline{{\eta }_{\overline{\overline{{\mathord{\buildrel{\lower1pt\hbox{$\scriptscriptstyle\smile$}}\over{\mathfrak{W}}}}_{{\mathfrak{L}}_{1}}}}}}}}^{l}\right)\right)}^{\Lambda }\right)}^{\frac{1}{\Lambda }}},1-{e}^{-{\left(\overline{\overline{{\theta }_{S}}}{\left(-\mathrm{log}\left(1-{\overline{\overline{{\eta }_{\overline{\overline{{\mathord{\buildrel{\lower1pt\hbox{$\scriptscriptstyle\smile$}}\over{\mathfrak{W}}}}_{{\mathfrak{L}}_{1}}}}}}}}^{u}\right)\right)}^{\Lambda }\right)}^{\frac{1}{\Lambda }}}\right]\end{array}\right)$$

### Definition 4

[13] For two IVA-IFNs $$\overline{\overline{{\mathord{\buildrel{\lower1pt\hbox{$\scriptscriptstyle\smile$}}\over{\mathfrak{W}}}}_{{\mathfrak{L}}_{\tau }}}}=\left(\left[{\overline{\overline{{\zeta }_{\overline{\overline{{\mathord{\buildrel{\lower1pt\hbox{$\scriptscriptstyle\smile$}}\over{\mathfrak{W}}}}_{{\mathfrak{L}}_{\tau }}}}}}}}^{l},{\overline{\overline{{\zeta }_{\overline{\overline{{\mathord{\buildrel{\lower1pt\hbox{$\scriptscriptstyle\smile$}}\over{\mathfrak{W}}}}_{{\mathfrak{L}}_{\tau }}}}}}}}^{u}\right],\left[{\overline{\overline{{\eta }_{\overline{\overline{{\mathord{\buildrel{\lower1pt\hbox{$\scriptscriptstyle\smile$}}\over{\mathfrak{W}}}}_{{\mathfrak{L}}_{\tau }}}}}}}}^{l},{\overline{\overline{{\eta }_{\overline{\overline{{\mathord{\buildrel{\lower1pt\hbox{$\scriptscriptstyle\smile$}}\over{\mathfrak{W}}}}_{{\mathfrak{L}}_{\tau }}}}}}}}^{u}\right]\right),\tau =\mathrm{1,2},$$ then,

7 $$\overline{\overline{{\Theta }_{SV}}}\left(\overline{\overline{{\mathord{\buildrel{\lower1pt\hbox{$\scriptscriptstyle\smile$}}\over{\mathfrak{W}}}}_{{\mathfrak{L}}_{\tau }}}}\right)=\frac{1}{2}\left({\overline{\overline{{\zeta }_{\overline{\overline{{\mathord{\buildrel{\lower1pt\hbox{$\scriptscriptstyle\smile$}}\over{\mathfrak{W}}}}_{{\mathfrak{L}}_{\tau }}}}}}}}^{l}-{\overline{\overline{{\eta }_{\overline{\overline{{\mathord{\buildrel{\lower1pt\hbox{$\scriptscriptstyle\smile$}}\over{\mathfrak{W}}}}_{{\mathfrak{L}}_{\tau }}}}}}}}^{l}+{\overline{\overline{{\zeta }_{\overline{\overline{{\mathord{\buildrel{\lower1pt\hbox{$\scriptscriptstyle\smile$}}\over{\mathfrak{W}}}}_{{\mathfrak{L}}_{\tau }}}}}}}}^{u}-{\overline{\overline{{\eta }_{\overline{\overline{{\mathord{\buildrel{\lower1pt\hbox{$\scriptscriptstyle\smile$}}\over{\mathfrak{W}}}}_{{\mathfrak{L}}_{\tau }}}}}}}}^{u}\right)\in \left[-\mathrm{1,1}\right]$$

8 $$\overline{\overline{{\Theta }_{AV}}}\left(\overline{\overline{{\mathord{\buildrel{\lower1pt\hbox{$\scriptscriptstyle\smile$}}\over{\mathfrak{W}}}}_{{\mathfrak{L}}_{\tau }}}}\right)=\frac{1}{2}\left({\overline{\overline{{\zeta }_{\overline{\overline{{\mathord{\buildrel{\lower1pt\hbox{$\scriptscriptstyle\smile$}}\over{\mathfrak{W}}}}_{{\mathfrak{L}}_{\tau }}}}}}}}^{l}+{\overline{\overline{{\eta }_{\overline{\overline{{\mathord{\buildrel{\lower1pt\hbox{$\scriptscriptstyle\smile$}}\over{\mathfrak{W}}}}_{{\mathfrak{L}}_{\tau }}}}}}}}^{l}+{\overline{\overline{{\zeta }_{\overline{\overline{{\mathord{\buildrel{\lower1pt\hbox{$\scriptscriptstyle\smile$}}\over{\mathfrak{W}}}}_{{\mathfrak{L}}_{\tau }}}}}}}}^{u}+{\overline{\overline{{\eta }_{\overline{\overline{{\mathord{\buildrel{\lower1pt\hbox{$\scriptscriptstyle\smile$}}\over{\mathfrak{W}}}}_{{\mathfrak{L}}_{\tau }}}}}}}}^{u}\right)\in \left[\mathrm{0,1}\right]$$

$$\overline{\overline{{\Theta }_{SV}}}\left(\overline{\overline{{\mathord{\buildrel{\lower1pt\hbox{$\scriptscriptstyle\smile$}}\over{\mathfrak{W}}}}_{{\mathfrak{L}}_{\tau }}}}\right)$$ and $$\overline{\overline{{\Theta }_{AV}}}\left(\overline{\overline{{\mathord{\buildrel{\lower1pt\hbox{$\scriptscriptstyle\smile$}}\over{\mathfrak{W}}}}_{{\mathfrak{L}}_{\tau }}}}\right)$$ represented the score function and accuracy function with some rules:

1. If $$\overline{\overline{{\Theta }_{SV}}}\left(\overline{\overline{{\mathord{\buildrel{\lower1pt\hbox{$\scriptscriptstyle\smile$}}\over{\mathfrak{W}}}}_{{\mathfrak{L}}_{1}}}}\right)>\overline{\overline{{\Theta }_{SV}}}\left(\overline{\overline{{\mathord{\buildrel{\lower1pt\hbox{$\scriptscriptstyle\smile$}}\over{\mathfrak{W}}}}_{{\mathfrak{L}}_{2}}}}\right)\Rightarrow \overline{\overline{{\mathord{\buildrel{\lower1pt\hbox{$\scriptscriptstyle\smile$}}\over{\mathfrak{W}}}}_{{\mathfrak{L}}_{1}}}}>\overline{\overline{{\mathord{\buildrel{\lower1pt\hbox{$\scriptscriptstyle\smile$}}\over{\mathfrak{W}}}}_{{\mathfrak{L}}_{2}}}}$$;
2. If $$\overline{\overline{{\Theta }_{SV}}}\left(\overline{\overline{{\mathord{\buildrel{\lower1pt\hbox{$\scriptscriptstyle\smile$}}\over{\mathfrak{W}}}}_{{\mathfrak{L}}_{1}}}}\right)<\overline{\overline{{\Theta }_{SV}}}\left(\overline{\overline{{\mathord{\buildrel{\lower1pt\hbox{$\scriptscriptstyle\smile$}}\over{\mathfrak{W}}}}_{{\mathfrak{L}}_{2}}}}\right)\Rightarrow \overline{\overline{{\mathord{\buildrel{\lower1pt\hbox{$\scriptscriptstyle\smile$}}\over{\mathfrak{W}}}}_{{\mathfrak{L}}_{1}}}}<\overline{\overline{{\mathord{\buildrel{\lower1pt\hbox{$\scriptscriptstyle\smile$}}\over{\mathfrak{W}}}}_{{\mathfrak{L}}_{2}}}}$$;
3. If $$\overline{\overline{{\Theta }_{SV}}}\left(\overline{\overline{{\mathord{\buildrel{\lower1pt\hbox{$\scriptscriptstyle\smile$}}\over{\mathfrak{W}}}}_{{\mathfrak{L}}_{1}}}}\right)=\overline{\overline{{\Theta }_{SV}}}\left(\overline{\overline{{\mathord{\buildrel{\lower1pt\hbox{$\scriptscriptstyle\smile$}}\over{\mathfrak{W}}}}_{{\mathfrak{L}}_{2}}}}\right)$$ then

If $$\overline{\overline{{\Theta }_{AV}}}\left(\overline{\overline{{\mathord{\buildrel{\lower1pt\hbox{$\scriptscriptstyle\smile$}}\over{\mathfrak{W}}}}_{{\mathfrak{L}}_{1}}}}\right)>\overline{\overline{{\Theta }_{AV}}}\left(\overline{\overline{{\mathord{\buildrel{\lower1pt\hbox{$\scriptscriptstyle\smile$}}\over{\mathfrak{W}}}}_{{\mathfrak{L}}_{2}}}}\right)\Rightarrow \overline{\overline{{\mathord{\buildrel{\lower1pt\hbox{$\scriptscriptstyle\smile$}}\over{\mathfrak{W}}}}_{{\mathfrak{L}}_{1}}}}>\overline{\overline{{\mathord{\buildrel{\lower1pt\hbox{$\scriptscriptstyle\smile$}}\over{\mathfrak{W}}}}_{{\mathfrak{L}}_{2}}}}$$;

If $$\overline{\overline{{\Theta }_{AV}}}\left(\overline{\overline{{\mathord{\buildrel{\lower1pt\hbox{$\scriptscriptstyle\smile$}}\over{\mathfrak{W}}}}_{{\mathfrak{L}}_{1}}}}\right)<\overline{\overline{{\Theta }_{AV}}}\left(\overline{\overline{{\mathord{\buildrel{\lower1pt\hbox{$\scriptscriptstyle\smile$}}\over{\mathfrak{W}}}}_{{\mathfrak{L}}_{2}}}}\right)\Rightarrow \overline{\overline{{\mathord{\buildrel{\lower1pt\hbox{$\scriptscriptstyle\smile$}}\over{\mathfrak{W}}}}_{{\mathfrak{L}}_{1}}}}<\overline{\overline{{\mathord{\buildrel{\lower1pt\hbox{$\scriptscriptstyle\smile$}}\over{\mathfrak{W}}}}_{{\mathfrak{L}}_{2}}}}$$;

If $$\overline{\overline{{\Theta }_{AV}}}\left(\overline{\overline{{\mathord{\buildrel{\lower1pt\hbox{$\scriptscriptstyle\smile$}}\over{\mathfrak{W}}}}_{{\mathfrak{L}}_{1}}}}\right)=\overline{\overline{{\Theta }_{AV}}}\left(\overline{\overline{{\mathord{\buildrel{\lower1pt\hbox{$\scriptscriptstyle\smile$}}\over{\mathfrak{W}}}}_{{\mathfrak{L}}_{2}}}}\right)\Rightarrow \overline{\overline{{\mathord{\buildrel{\lower1pt\hbox{$\scriptscriptstyle\smile$}}\over{\mathfrak{W}}}}_{{\mathfrak{L}}_{1}}}}=\overline{\overline{{\mathord{\buildrel{\lower1pt\hbox{$\scriptscriptstyle\smile$}}\over{\mathfrak{W}}}}_{{\mathfrak{L}}_{2}}}}$$.

## Aczel–Alsina PA Operators for IVA-IFSs

The Aczel–Alsina t-norm and t-conorm and the PA operator are very famous and valuable tools for getting the finest preferences from the collection of preferences. In this section, we propose the IVA-IFAAPA, IVA-IFAAPOA, IVA-IFAAPG, and IVA-IFAAPOG operators. Moreover, we discuss some of the properties of the presented operators such as idempotency, monotonicity, and boundedness.

### Definition 5

For a group of IVA-IFNs $$\overline{\overline{{\mathord{\buildrel{\lower1pt\hbox{$\scriptscriptstyle\smile$}}\over{\mathfrak{W}}}}_{{\mathfrak{L}}_{\tau }}}}=\left(\left[{\overline{\overline{{\zeta }_{\overline{\overline{{\mathord{\buildrel{\lower1pt\hbox{$\scriptscriptstyle\smile$}}\over{\mathfrak{W}}}}_{{\mathfrak{L}}_{\tau }}}}}}}}^{l},{\overline{\overline{{\zeta }_{\overline{\overline{{\mathord{\buildrel{\lower1pt\hbox{$\scriptscriptstyle\smile$}}\over{\mathfrak{W}}}}_{{\mathfrak{L}}_{\tau }}}}}}}}^{u}\right],\left[{\overline{\overline{{\eta }_{\overline{\overline{{\mathord{\buildrel{\lower1pt\hbox{$\scriptscriptstyle\smile$}}\over{\mathfrak{W}}}}_{{\mathfrak{L}}_{\tau }}}}}}}}^{l},{\overline{\overline{{\eta }_{\overline{\overline{{\mathord{\buildrel{\lower1pt\hbox{$\scriptscriptstyle\smile$}}\over{\mathfrak{W}}}}_{{\mathfrak{L}}_{\tau }}}}}}}}^{u}\right]\right),\tau =\mathrm{1,2},\dots ,{\rm Z},$$ then an IVA-IFAAPA operator is described as

9 $$IVA-IFAAPA\left(\overline{\overline{{\mathord{\buildrel{\lower1pt\hbox{$\scriptscriptstyle\smile$}}\over{\mathfrak{W}}}}_{{\mathfrak{L}}_{1}}}},\overline{\overline{{\mathord{\buildrel{\lower1pt\hbox{$\scriptscriptstyle\smile$}}\over{\mathfrak{W}}}}_{{\mathfrak{L}}_{2}}}},\dots ,\overline{\overline{{\mathord{\buildrel{\lower1pt\hbox{$\scriptscriptstyle\smile$}}\over{\mathfrak{W}}}}_{{\mathfrak{L}}_{\rm Z}}}}\right)=\overline{\overline{{\Xi }_{1}}}\overline{\overline{{\mathord{\buildrel{\lower1pt\hbox{$\scriptscriptstyle\smile$}}\over{\mathfrak{W}}}}_{{\mathfrak{L}}_{1}}}}\oplus \overline{\overline{{\Xi }_{2}}}\overline{\overline{{\mathord{\buildrel{\lower1pt\hbox{$\scriptscriptstyle\smile$}}\over{\mathfrak{W}}}}_{{\mathfrak{L}}_{2}}}}\oplus \dots \oplus \overline{\overline{{\Xi }_{\rm Z}}}\overline{\overline{{\mathord{\buildrel{\lower1pt\hbox{$\scriptscriptstyle\smile$}}\over{\mathfrak{W}}}}_{{\mathfrak{L}}_{\rm Z}}}}={\oplus }_{\tau =1}^{\rm Z}\left(\overline{\overline{{\Xi }_{\tau }}}\overline{\overline{{\mathord{\buildrel{\lower1pt\hbox{$\scriptscriptstyle\smile$}}\over{\mathfrak{W}}}}_{{\mathfrak{L}}_{\tau }}}}\right)$$

Noticed that

10 $$\overline{\overline{{\Xi }_{\tau }}}=\frac{\left(1+\overline{\overline{\mathfrak{I}}}\left(\overline{\overline{{\mathord{\buildrel{\lower1pt\hbox{$\scriptscriptstyle\smile$}}\over{\mathfrak{W}}}}_{{\mathfrak{L}}_{\tau }}}}\right)\right)}{\sum_{\tau =1}^{\rm Z}\left(1+\overline{\overline{\mathfrak{I}}}\left(\overline{\overline{{\mathord{\buildrel{\lower1pt\hbox{$\scriptscriptstyle\smile$}}\over{\mathfrak{W}}}}_{{\mathfrak{L}}_{\tau }}}}\right)\right)}$$

where $$\overline{\overline{\mathfrak{I}}}\left(\overline{\overline{{\mathord{\buildrel{\lower1pt\hbox{$\scriptscriptstyle\smile$}}\over{\mathfrak{W}}}}_{{\mathfrak{L}}_{\tau }}}}\right)=\sum_{\begin{array}{c}s=1,\\ s\ne \tau \end{array}}^{\rm Z}Sup\left(\overline{\overline{{\mathord{\buildrel{\lower1pt\hbox{$\scriptscriptstyle\smile$}}\over{\mathfrak{W}}}}_{{\mathfrak{L}}_{\tau }}}},\overline{\overline{{\mathord{\buildrel{\lower1pt\hbox{$\scriptscriptstyle\smile$}}\over{\mathfrak{W}}}}_{{\mathfrak{L}}_{k}}}}\right)$$which expresses the relationship between $$\overline{\overline{{\mathord{\buildrel{\lower1pt\hbox{$\scriptscriptstyle\smile$}}\over{\mathfrak{W}}}}_{{\mathfrak{L}}_{\tau }}}}$$ and $$\overline{\overline{{\mathord{\buildrel{\lower1pt\hbox{$\scriptscriptstyle\smile$}}\over{\mathfrak{W}}}}_{{\mathfrak{L}}_{k}}}}$$ with various characteristics:

1. $$Sup\left(\overline{\overline{{\mathord{\buildrel{\lower1pt\hbox{$\scriptscriptstyle\smile$}}\over{\mathfrak{W}}}}_{{\mathfrak{L}}_{\tau }}}},\overline{\overline{{\mathord{\buildrel{\lower1pt\hbox{$\scriptscriptstyle\smile$}}\over{\mathfrak{W}}}}_{{\mathfrak{L}}_{k}}}}\right)\in \left[\mathrm{0,1}\right]$$.
2. $$Sup\left(\overline{\overline{{\mathord{\buildrel{\lower1pt\hbox{$\scriptscriptstyle\smile$}}\over{\mathfrak{W}}}}_{{\mathfrak{L}}_{\tau }}}},\overline{\overline{{\mathord{\buildrel{\lower1pt\hbox{$\scriptscriptstyle\smile$}}\over{\mathfrak{W}}}}_{{\mathfrak{L}}_{k}}}}\right)=Sup\left(\overline{\overline{{\mathord{\buildrel{\lower1pt\hbox{$\scriptscriptstyle\smile$}}\over{\mathfrak{W}}}}_{{\mathfrak{L}}_{k}}}},\overline{\overline{{\mathord{\buildrel{\lower1pt\hbox{$\scriptscriptstyle\smile$}}\over{\mathfrak{W}}}}_{{\mathfrak{L}}_{\tau }}}}\right)$$.
3. $$Sup\left(\overline{\overline{{\mathord{\buildrel{\lower1pt\hbox{$\scriptscriptstyle\smile$}}\over{\mathfrak{W}}}}_{{\mathfrak{L}}_{\tau }}}},\overline{\overline{{\mathord{\buildrel{\lower1pt\hbox{$\scriptscriptstyle\smile$}}\over{\mathfrak{W}}}}_{{\mathfrak{L}}_{k}}}}\right)\ge Sup\left({\overline{\overline{{\mathord{\buildrel{\lower1pt\hbox{$\scriptscriptstyle\smile$}}\over{\mathfrak{W}}}}_{{\mathfrak{L}}_{\tau }}}}}^{*},{\overline{\overline{{\mathord{\buildrel{\lower1pt\hbox{$\scriptscriptstyle\smile$}}\over{\mathfrak{W}}}}_{{\mathfrak{L}}_{k}}}}}^{*}\right)$$ when $$\left|\overline{\overline{{\mathord{\buildrel{\lower1pt\hbox{$\scriptscriptstyle\smile$}}\over{\mathfrak{W}}}}_{{\mathfrak{L}}_{\tau }}}}-\overline{\overline{{\mathord{\buildrel{\lower1pt\hbox{$\scriptscriptstyle\smile$}}\over{\mathfrak{W}}}}_{{\mathfrak{L}}_{k}}}}\right|\le \left|{\overline{\overline{{\mathord{\buildrel{\lower1pt\hbox{$\scriptscriptstyle\smile$}}\over{\mathfrak{W}}}}_{{\mathfrak{L}}_{\tau }}}}}^{*}-{\overline{\overline{{\mathord{\buildrel{\lower1pt\hbox{$\scriptscriptstyle\smile$}}\over{\mathfrak{W}}}}_{{\mathfrak{L}}_{k}}}}}^{*}\right|$$

where $$Sup\left(\overline{\overline{{\mathord{\buildrel{\lower1pt\hbox{$\scriptscriptstyle\smile$}}\over{\mathfrak{W}}}}_{{\mathfrak{L}}_{\tau }}}},\overline{\overline{{\mathord{\buildrel{\lower1pt\hbox{$\scriptscriptstyle\smile$}}\over{\mathfrak{W}}}}_{{\mathfrak{L}}_{k}}}}\right)=1-Dis\left(\overline{\overline{{\mathord{\buildrel{\lower1pt\hbox{$\scriptscriptstyle\smile$}}\over{\mathfrak{W}}}}_{{\mathfrak{L}}_{\tau }}}},\overline{\overline{{\mathord{\buildrel{\lower1pt\hbox{$\scriptscriptstyle\smile$}}\over{\mathfrak{W}}}}_{{\mathfrak{L}}_{k}}}}\right)$$ and

11 $$Dis\left(\overline{\overline{{\mathord{\buildrel{\lower1pt\hbox{$\scriptscriptstyle\smile$}}\over{\mathfrak{W}}}}_{{\mathfrak{L}}_{\tau }}}},\overline{\overline{{\mathord{\buildrel{\lower1pt\hbox{$\scriptscriptstyle\smile$}}\over{\mathfrak{W}}}}_{{\mathfrak{L}}_{k}}}}\right)=\frac{1}{4}\left(\left|{\overline{\overline{{\zeta }_{\overline{\overline{{\mathord{\buildrel{\lower1pt\hbox{$\scriptscriptstyle\smile$}}\over{\mathfrak{W}}}}_{{\mathfrak{L}}_{\tau }}}}}}}}^{l}-{\overline{\overline{{\zeta }_{\overline{\overline{{\mathord{\buildrel{\lower1pt\hbox{$\scriptscriptstyle\smile$}}\over{\mathfrak{W}}}}_{{\mathfrak{L}}_{k}}}}}}}}^{l}\right|+\left|{\overline{\overline{{\eta }_{\overline{\overline{{\mathord{\buildrel{\lower1pt\hbox{$\scriptscriptstyle\smile$}}\over{\mathfrak{W}}}}_{{\mathfrak{L}}_{\tau }}}}}}}}^{l}-{\overline{\overline{{\eta }_{\overline{\overline{{\mathord{\buildrel{\lower1pt\hbox{$\scriptscriptstyle\smile$}}\over{\mathfrak{W}}}}_{{\mathfrak{L}}_{k}}}}}}}}^{l}\right|+\left|{\overline{\overline{{\zeta }_{\overline{\overline{{\mathord{\buildrel{\lower1pt\hbox{$\scriptscriptstyle\smile$}}\over{\mathfrak{W}}}}_{{\mathfrak{L}}_{\tau }}}}}}}}^{u}-{\overline{\overline{{\zeta }_{\overline{\overline{{\mathord{\buildrel{\lower1pt\hbox{$\scriptscriptstyle\smile$}}\over{\mathfrak{W}}}}_{{\mathfrak{L}}_{k}}}}}}}}^{u}\right|+\left|{\overline{\overline{{\eta }_{\overline{\overline{{\mathord{\buildrel{\lower1pt\hbox{$\scriptscriptstyle\smile$}}\over{\mathfrak{W}}}}_{{\mathfrak{L}}_{\tau }}}}}}}}^{u}-{\overline{\overline{{\eta }_{\overline{\overline{{\mathord{\buildrel{\lower1pt\hbox{$\scriptscriptstyle\smile$}}\over{\mathfrak{W}}}}_{{\mathfrak{L}}_{k}}}}}}}}^{u}\right|\right)$$

where Eq. (11) represents the distance measures between $$\overline{\overline{{\mathord{\buildrel{\lower1pt\hbox{$\scriptscriptstyle\smile$}}\over{\mathfrak{W}}}}_{{\mathfrak{L}}_{\tau }}}}$$ and $$\overline{\overline{{\mathord{\buildrel{\lower1pt\hbox{$\scriptscriptstyle\smile$}}\over{\mathfrak{W}}}}_{{\mathfrak{L}}_{k}}}}$$.

### Theorem 1

For a group of IVA-IFNs $$\overline{\overline{{\mathord{\buildrel{\lower1pt\hbox{$\scriptscriptstyle\smile$}}\over{\mathfrak{W}}}}_{{\mathfrak{L}}_{\tau }}}}=\left(\left[{\overline{\overline{{\zeta }_{\overline{\overline{{\mathord{\buildrel{\lower1pt\hbox{$\scriptscriptstyle\smile$}}\over{\mathfrak{W}}}}_{{\mathfrak{L}}_{\tau }}}}}}}}^{l},{\overline{\overline{{\zeta }_{\overline{\overline{{\mathord{\buildrel{\lower1pt\hbox{$\scriptscriptstyle\smile$}}\over{\mathfrak{W}}}}_{{\mathfrak{L}}_{\tau }}}}}}}}^{u}\right],\left[{\overline{\overline{{\eta }_{\overline{\overline{{\mathord{\buildrel{\lower1pt\hbox{$\scriptscriptstyle\smile$}}\over{\mathfrak{W}}}}_{{\mathfrak{L}}_{\tau }}}}}}}}^{l},{\overline{\overline{{\eta }_{\overline{\overline{{\mathord{\buildrel{\lower1pt\hbox{$\scriptscriptstyle\smile$}}\over{\mathfrak{W}}}}_{{\mathfrak{L}}_{\tau }}}}}}}}^{u}\right]\right),\tau =\mathrm{1,2},\dots ,{\rm Z},$$ the aggregation result from Eq. (9) is still an IVA-IFN, such as

12 $$IVA-IFAAPA\left(\overline{\overline{{\mathord{\buildrel{\lower1pt\hbox{$\scriptscriptstyle\smile$}}\over{\mathfrak{W}}}}_{{\mathfrak{L}}_{1}}}},\overline{\overline{{\mathord{\buildrel{\lower1pt\hbox{$\scriptscriptstyle\smile$}}\over{\mathfrak{W}}}}_{{\mathfrak{L}}_{2}}}},\dots ,\overline{\overline{{\mathord{\buildrel{\lower1pt\hbox{$\scriptscriptstyle\smile$}}\over{\mathfrak{W}}}}_{{\mathfrak{L}}_{\rm Z}}}}\right)=\left(\begin{array}{c}\left[1-{e}^{-{\left(\sum_{\tau =1}^{\rm Z}\overline{\overline{{\Xi }_{\tau }}}{\left(-\mathrm{log}\left(1-{\overline{\overline{{\zeta }_{\overline{\overline{{\mathord{\buildrel{\lower1pt\hbox{$\scriptscriptstyle\smile$}}\over{\mathfrak{W}}}}_{{\mathfrak{L}}_{\tau }}}}}}}}^{l}\right)\right)}^{\Lambda }\right)}^{\frac{1}{\Lambda }}},1-{e}^{-{\left(\sum_{\tau =1}^{\rm Z}\overline{\overline{{\Xi }_{\tau }}}{\left(-\mathrm{log}\left(1-{\overline{\overline{{\zeta }_{\overline{\overline{{\mathord{\buildrel{\lower1pt\hbox{$\scriptscriptstyle\smile$}}\over{\mathfrak{W}}}}_{{\mathfrak{L}}_{\tau }}}}}}}}^{u}\right)\right)}^{\Lambda }\right)}^{\frac{1}{\Lambda }}}\right],\\ \left[{e}^{-{\left(\sum_{\tau =1}^{\rm Z}\overline{\overline{{\Xi }_{\tau }}}{\left(-\mathrm{log}\left({\overline{\overline{{\eta }_{\overline{\overline{{\mathord{\buildrel{\lower1pt\hbox{$\scriptscriptstyle\smile$}}\over{\mathfrak{W}}}}_{{\mathfrak{L}}_{\tau }}}}}}}}^{l}\right)\right)}^{\Lambda }\right)}^{\frac{1}{\Lambda }}},{e}^{-{\left(\sum_{\tau =1}^{\rm Z}\overline{\overline{{\Xi }_{\tau }}}{\left(-\mathrm{log}\left({\overline{\overline{{\eta }_{\overline{\overline{{\mathord{\buildrel{\lower1pt\hbox{$\scriptscriptstyle\smile$}}\over{\mathfrak{W}}}}_{{\mathfrak{L}}_{\tau }}}}}}}}^{u}\right)\right)}^{\Lambda }\right)}^{\frac{1}{\Lambda }}}\right]\end{array}\right)$$

### Proof

(Using Mathematical induction).

For ${\rm Z}=2$, we have

$$\overline{\overline{{\Xi }_{1}}}\overline{\overline{{\mathord{\buildrel{\lower1pt\hbox{$\scriptscriptstyle\smile$}}\over{\mathfrak{W}}}}_{{\mathfrak{L}}_{1}}}}=\left(\begin{array}{c}\left[1-{e}^{-{\left(\overline{\overline{{\Xi }_{1}}}{\left(-\mathrm{log}\left(1-{\overline{\overline{{\zeta }_{\overline{\overline{{\mathord{\buildrel{\lower1pt\hbox{$\scriptscriptstyle\smile$}}\over{\mathfrak{W}}}}_{{\mathfrak{L}}_{1}}}}}}}}^{l}\right)\right)}^{\Lambda }\right)}^{\frac{1}{\Lambda }}},1-{e}^{-{\left(\overline{\overline{{\Xi }_{1}}}{\left(-\mathrm{log}\left(1-{\overline{\overline{{\zeta }_{\overline{\overline{{\mathord{\buildrel{\lower1pt\hbox{$\scriptscriptstyle\smile$}}\over{\mathfrak{W}}}}_{{\mathfrak{L}}_{1}}}}}}}}^{u}\right)\right)}^{\Lambda }\right)}^{\frac{1}{\Lambda }}}\right],\\ \left[{e}^{-{\left(\overline{\overline{{\Xi }_{1}}}{\left(-\mathrm{log}\left({\overline{\overline{{\eta }_{\overline{\overline{{\mathord{\buildrel{\lower1pt\hbox{$\scriptscriptstyle\smile$}}\over{\mathfrak{W}}}}_{{\mathfrak{L}}_{1}}}}}}}}^{l}\right)\right)}^{\Lambda }\right)}^{\frac{1}{\Lambda }}},{e}^{-{\left(\overline{\overline{{\Xi }_{1}}}{\left(-\mathrm{log}\left({\overline{\overline{{\eta }_{\overline{\overline{{\mathord{\buildrel{\lower1pt\hbox{$\scriptscriptstyle\smile$}}\over{\mathfrak{W}}}}_{{\mathfrak{L}}_{1}}}}}}}}^{u}\right)\right)}^{\Lambda }\right)}^{\frac{1}{\Lambda }}}\right]\end{array}\right)$$

$$\overline{\overline{{\Xi }_{2}}}\overline{\overline{{\mathord{\buildrel{\lower1pt\hbox{$\scriptscriptstyle\smile$}}\over{\mathfrak{W}}}}_{{\mathfrak{L}}_{2}}}}=\left(\begin{array}{c}\left[1-{e}^{-{\left(\overline{\overline{{\Xi }_{2}}}{\left(-\mathrm{log}\left(1-{\overline{\overline{{\zeta }_{\overline{\overline{{\mathord{\buildrel{\lower1pt\hbox{$\scriptscriptstyle\smile$}}\over{\mathfrak{W}}}}_{{\mathfrak{L}}_{2}}}}}}}}^{l}\right)\right)}^{\Lambda }\right)}^{\frac{1}{\Lambda }}},1-{e}^{-{\left(\overline{\overline{{\Xi }_{2}}}{\left(-\mathrm{log}\left(1-{\overline{\overline{{\zeta }_{\overline{\overline{{\mathord{\buildrel{\lower1pt\hbox{$\scriptscriptstyle\smile$}}\over{\mathfrak{W}}}}_{{\mathfrak{L}}_{2}}}}}}}}^{u}\right)\right)}^{\Lambda }\right)}^{\frac{1}{\Lambda }}}\right],\\ \left[{e}^{-{\left(\overline{\overline{{\Xi }_{2}}}{\left(-\mathrm{log}\left({\overline{\overline{{\eta }_{\overline{\overline{{\mathord{\buildrel{\lower1pt\hbox{$\scriptscriptstyle\smile$}}\over{\mathfrak{W}}}}_{{\mathfrak{L}}_{2}}}}}}}}^{l}\right)\right)}^{\Lambda }\right)}^{\frac{1}{\Lambda }}},{e}^{-{\left(\overline{\overline{{\Xi }_{2}}}{\left(-\mathrm{log}\left({\overline{\overline{{\eta }_{\overline{\overline{{\mathord{\buildrel{\lower1pt\hbox{$\scriptscriptstyle\smile$}}\over{\mathfrak{W}}}}_{{\mathfrak{L}}_{2}}}}}}}}^{u}\right)\right)}^{\Lambda }\right)}^{\frac{1}{\Lambda }}}\right]\end{array}\right)$$

Thus, we have

$$IVA-IFAAPA\left(\overline{\overline{{\mathord{\buildrel{\lower1pt\hbox{$\scriptscriptstyle\smile$}}\over{\mathfrak{W}}}}_{{\mathfrak{L}}_{1}}}},\overline{\overline{{\mathord{\buildrel{\lower1pt\hbox{$\scriptscriptstyle\smile$}}\over{\mathfrak{W}}}}_{{\mathfrak{L}}_{2}}}}\right)=\overline{\overline{{\Xi }_{1}}}\overline{\overline{{\mathord{\buildrel{\lower1pt\hbox{$\scriptscriptstyle\smile$}}\over{\mathfrak{W}}}}_{{\mathfrak{L}}_{1}}}}\oplus \overline{\overline{{\Xi }_{2}}}\overline{\overline{{\mathord{\buildrel{\lower1pt\hbox{$\scriptscriptstyle\smile$}}\over{\mathfrak{W}}}}_{{\mathfrak{L}}_{2}}}}$$

$$=\left(\begin{array}{c}\left[1-{e}^{-{\left(\overline{\overline{{\Xi }_{1}}}{\left(-\mathrm{log}\left(1-{\overline{\overline{{\zeta }_{\overline{\overline{{\mathord{\buildrel{\lower1pt\hbox{$\scriptscriptstyle\smile$}}\over{\mathfrak{W}}}}_{{\mathfrak{L}}_{1}}}}}}}}^{l}\right)\right)}^{\Lambda }\right)}^{\frac{1}{\Lambda }}},1-{e}^{-{\left(\overline{\overline{{\Xi }_{1}}}{\left(-\mathrm{log}\left(1-{\overline{\overline{{\zeta }_{\overline{\overline{{\mathord{\buildrel{\lower1pt\hbox{$\scriptscriptstyle\smile$}}\over{\mathfrak{W}}}}_{{\mathfrak{L}}_{1}}}}}}}}^{u}\right)\right)}^{\Lambda }\right)}^{\frac{1}{\Lambda }}}\right],\\ \left[{e}^{-{\left(\overline{\overline{{\Xi }_{1}}}{\left(-\mathrm{log}\left({\overline{\overline{{\eta }_{\overline{\overline{{\mathord{\buildrel{\lower1pt\hbox{$\scriptscriptstyle\smile$}}\over{\mathfrak{W}}}}_{{\mathfrak{L}}_{1}}}}}}}}^{l}\right)\right)}^{\Lambda }\right)}^{\frac{1}{\Lambda }}},{e}^{-{\left(\overline{\overline{{\Xi }_{1}}}{\left(-\mathrm{log}\left({\overline{\overline{{\eta }_{\overline{\overline{{\mathord{\buildrel{\lower1pt\hbox{$\scriptscriptstyle\smile$}}\over{\mathfrak{W}}}}_{{\mathfrak{L}}_{1}}}}}}}}^{u}\right)\right)}^{\Lambda }\right)}^{\frac{1}{\Lambda }}}\right]\end{array}\right)\oplus \left(\begin{array}{c}\left[1-{e}^{-{\left(\overline{\overline{{\Xi }_{2}}}{\left(-\mathrm{log}\left(1-{\overline{\overline{{\zeta }_{\overline{\overline{{\mathord{\buildrel{\lower1pt\hbox{$\scriptscriptstyle\smile$}}\over{\mathfrak{W}}}}_{{\mathfrak{L}}_{2}}}}}}}}^{l}\right)\right)}^{\Lambda }\right)}^{\frac{1}{\Lambda }}},1-{e}^{-{\left(\overline{\overline{{\Xi }_{2}}}{\left(-\mathrm{log}\left(1-{\overline{\overline{{\zeta }_{\overline{\overline{{\mathord{\buildrel{\lower1pt\hbox{$\scriptscriptstyle\smile$}}\over{\mathfrak{W}}}}_{{\mathfrak{L}}_{2}}}}}}}}^{u}\right)\right)}^{\Lambda }\right)}^{\frac{1}{\Lambda }}}\right],\\ \left[{e}^{-{\left(\overline{\overline{{\Xi }_{2}}}{\left(-\mathrm{log}\left({\overline{\overline{{\eta }_{\overline{\overline{{\mathord{\buildrel{\lower1pt\hbox{$\scriptscriptstyle\smile$}}\over{\mathfrak{W}}}}_{{\mathfrak{L}}_{2}}}}}}}}^{l}\right)\right)}^{\Lambda }\right)}^{\frac{1}{\Lambda }}},{e}^{-{\left(\overline{\overline{{\Xi }_{2}}}{\left(-\mathrm{log}\left({\overline{\overline{{\eta }_{\overline{\overline{{\mathord{\buildrel{\lower1pt\hbox{$\scriptscriptstyle\smile$}}\over{\mathfrak{W}}}}_{{\mathfrak{L}}_{2}}}}}}}}^{u}\right)\right)}^{\Lambda }\right)}^{\frac{1}{\Lambda }}}\right]\end{array}\right)$$

$$=\left(\begin{array}{c}\left[1-{e}^{-{\left(\sum_{\tau =1}^{2}\overline{\overline{{\Xi }_{\tau }}}{\left(-\mathrm{log}\left(1-{\overline{\overline{{\zeta }_{\overline{\overline{{\mathord{\buildrel{\lower1pt\hbox{$\scriptscriptstyle\smile$}}\over{\mathfrak{W}}}}_{{\mathfrak{L}}_{\tau }}}}}}}}^{l}\right)\right)}^{\Lambda }\right)}^{\frac{1}{\Lambda }}},1-{e}^{-{\left(\sum_{\tau =1}^{2}\overline{\overline{{\Xi }_{\tau }}}{\left(-\mathrm{log}\left(1-{\overline{\overline{{\zeta }_{\overline{\overline{{\mathord{\buildrel{\lower1pt\hbox{$\scriptscriptstyle\smile$}}\over{\mathfrak{W}}}}_{{\mathfrak{L}}_{\tau }}}}}}}}^{u}\right)\right)}^{\Lambda }\right)}^{\frac{1}{\Lambda }}}\right],\\ \left[{e}^{-{\left(\sum_{\tau =1}^{2}\overline{\overline{{\Xi }_{\tau }}}{\left(-\mathrm{log}\left({\overline{\overline{{\eta }_{\overline{\overline{{\mathord{\buildrel{\lower1pt\hbox{$\scriptscriptstyle\smile$}}\over{\mathfrak{W}}}}_{{\mathfrak{L}}_{\tau }}}}}}}}^{l}\right)\right)}^{\Lambda }\right)}^{\frac{1}{\Lambda }}},{e}^{-{\left(\sum_{\tau =1}^{2}\overline{\overline{{\Xi }_{\tau }}}{\left(-\mathrm{log}\left({\overline{\overline{{\eta }_{\overline{\overline{{\mathord{\buildrel{\lower1pt\hbox{$\scriptscriptstyle\smile$}}\over{\mathfrak{W}}}}_{{\mathfrak{L}}_{\tau }}}}}}}}^{u}\right)\right)}^{\Lambda }\right)}^{\frac{1}{\Lambda }}}\right]\end{array}\right)$$

For ${\rm Z}=2,$ (12) is kept.for ${\rm Z}=k$, we assume that (12) is right, such as

$$IVA-IFAAPA\left(\overline{\overline{{\mathord{\buildrel{\lower1pt\hbox{$\scriptscriptstyle\smile$}}\over{\mathfrak{W}}}}_{{\mathfrak{L}}_{1}}}},\overline{\overline{{\mathord{\buildrel{\lower1pt\hbox{$\scriptscriptstyle\smile$}}\over{\mathfrak{W}}}}_{{\mathfrak{L}}_{2}}}},\dots ,\overline{\overline{{\mathord{\buildrel{\lower1pt\hbox{$\scriptscriptstyle\smile$}}\over{\mathfrak{W}}}}_{{\mathfrak{L}}_{k}}}}\right)=\left(\begin{array}{c}\left[1-{e}^{-{\left(\sum_{\tau =1}^{k}\overline{\overline{{\Xi }_{\tau }}}{\left(-\mathrm{log}\left(1-{\overline{\overline{{\zeta }_{\overline{\overline{{\mathord{\buildrel{\lower1pt\hbox{$\scriptscriptstyle\smile$}}\over{\mathfrak{W}}}}_{{\mathfrak{L}}_{\tau }}}}}}}}^{l}\right)\right)}^{\Lambda }\right)}^{\frac{1}{\Lambda }}},1-{e}^{-{\left(\sum_{\tau =1}^{k}\overline{\overline{{\Xi }_{\tau }}}{\left(-\mathrm{log}\left(1-{\overline{\overline{{\zeta }_{\overline{\overline{{\mathord{\buildrel{\lower1pt\hbox{$\scriptscriptstyle\smile$}}\over{\mathfrak{W}}}}_{{\mathfrak{L}}_{\tau }}}}}}}}^{u}\right)\right)}^{\Lambda }\right)}^{\frac{1}{\Lambda }}}\right],\\ \left[{e}^{-{\left(\sum_{\tau =1}^{k}\overline{\overline{{\Xi }_{\tau }}}{\left(-\mathrm{log}\left({\overline{\overline{{\eta }_{\overline{\overline{{\mathord{\buildrel{\lower1pt\hbox{$\scriptscriptstyle\smile$}}\over{\mathfrak{W}}}}_{{\mathfrak{L}}_{\tau }}}}}}}}^{l}\right)\right)}^{\Lambda }\right)}^{\frac{1}{\Lambda }}},{e}^{-{\left(\sum_{\tau =1}^{k}\overline{\overline{{\Xi }_{\tau }}}{\left(-\mathrm{log}\left({\overline{\overline{{\eta }_{\overline{\overline{{\mathord{\buildrel{\lower1pt\hbox{$\scriptscriptstyle\smile$}}\over{\mathfrak{W}}}}_{{\mathfrak{L}}_{\tau }}}}}}}}^{u}\right)\right)}^{\Lambda }\right)}^{\frac{1}{\Lambda }}}\right]\end{array}\right)$$

Then, for ${\rm Z}=k+1$, we aim to derive our required result such as

$$IVA-IFAAPA\left(\overline{\overline{{\mathord{\buildrel{\lower1pt\hbox{$\scriptscriptstyle\smile$}}\over{\mathfrak{W}}}}_{{\mathfrak{L}}_{1}}}},\overline{\overline{{\mathord{\buildrel{\lower1pt\hbox{$\scriptscriptstyle\smile$}}\over{\mathfrak{W}}}}_{{\mathfrak{L}}_{2}}}},\dots ,\overline{\overline{{\mathord{\buildrel{\lower1pt\hbox{$\scriptscriptstyle\smile$}}\over{\mathfrak{W}}}}_{{\mathfrak{L}}_{k+1}}}}\right)={\oplus }_{\tau =1}^{k+1}\left(\overline{\overline{{\Xi }_{\tau }}}\overline{\overline{{\mathord{\buildrel{\lower1pt\hbox{$\scriptscriptstyle\smile$}}\over{\mathfrak{W}}}}_{{\mathfrak{L}}_{\tau }}}}\right)={\oplus }_{\tau =1}^{k}\left(\overline{\overline{{\Xi }_{\tau }}}\overline{\overline{{\mathord{\buildrel{\lower1pt\hbox{$\scriptscriptstyle\smile$}}\over{\mathfrak{W}}}}_{{\mathfrak{L}}_{\tau }}}}\right)\oplus \overline{\overline{{\Xi }_{k+1}}}\overline{\overline{{\mathord{\buildrel{\lower1pt\hbox{$\scriptscriptstyle\smile$}}\over{\mathfrak{W}}}}_{{\mathfrak{L}}_{k+1}}}}$$

$$=\left(\begin{array}{c}\left[1-{e}^{-{\left(\sum_{\tau =1}^{k}\overline{\overline{{\Xi }_{\tau }}}{\left(-\mathrm{log}\left(1-{\overline{\overline{{\zeta }_{\overline{\overline{{\mathord{\buildrel{\lower1pt\hbox{$\scriptscriptstyle\smile$}}\over{\mathfrak{W}}}}_{{\mathfrak{L}}_{\tau }}}}}}}}^{l}\right)\right)}^{\Lambda }\right)}^{\frac{1}{\Lambda }}},1-{e}^{-{\left(\sum_{\tau =1}^{k}\overline{\overline{{\Xi }_{\tau }}}{\left(-\mathrm{log}\left(1-{\overline{\overline{{\zeta }_{\overline{\overline{{\mathord{\buildrel{\lower1pt\hbox{$\scriptscriptstyle\smile$}}\over{\mathfrak{W}}}}_{{\mathfrak{L}}_{\tau }}}}}}}}^{u}\right)\right)}^{\Lambda }\right)}^{\frac{1}{\Lambda }}}\right],\\ \left[{e}^{-{\left(\sum_{\tau =1}^{k}\overline{\overline{{\Xi }_{\tau }}}{\left(-\mathrm{log}\left({\overline{\overline{{\eta }_{\overline{\overline{{\mathord{\buildrel{\lower1pt\hbox{$\scriptscriptstyle\smile$}}\over{\mathfrak{W}}}}_{{\mathfrak{L}}_{\tau }}}}}}}}^{l}\right)\right)}^{\Lambda }\right)}^{\frac{1}{\Lambda }}},{e}^{-{\left(\sum_{\tau =1}^{k}\overline{\overline{{\Xi }_{\tau }}}{\left(-\mathrm{log}\left({\overline{\overline{{\eta }_{\overline{\overline{{\mathord{\buildrel{\lower1pt\hbox{$\scriptscriptstyle\smile$}}\over{\mathfrak{W}}}}_{{\mathfrak{L}}_{\tau }}}}}}}}^{u}\right)\right)}^{\Lambda }\right)}^{\frac{1}{\Lambda }}}\right]\end{array}\right)\oplus \left(\begin{array}{c}\left[1-{e}^{-{\left(\overline{\overline{{\Xi }_{k+1}}}{\left(-\mathrm{log}\left(1-{\overline{\overline{{\zeta }_{\overline{\overline{{\mathord{\buildrel{\lower1pt\hbox{$\scriptscriptstyle\smile$}}\over{\mathfrak{W}}}}_{{\mathfrak{L}}_{k+1}}}}}}}}^{l}\right)\right)}^{\Lambda }\right)}^{\frac{1}{\Lambda }}},1-{e}^{-{\left(\overline{\overline{{\Xi }_{k+1}}}{\left(-\mathrm{log}\left(1-{\overline{\overline{{\zeta }_{\overline{\overline{{\mathord{\buildrel{\lower1pt\hbox{$\scriptscriptstyle\smile$}}\over{\mathfrak{W}}}}_{{\mathfrak{L}}_{k+1}}}}}}}}^{u}\right)\right)}^{\Lambda }\right)}^{\frac{1}{\Lambda }}}\right],\\ \left[{e}^{-{\left(\overline{\overline{{\Xi }_{k+1}}}{\left(-\mathrm{log}\left({\overline{\overline{{\eta }_{\overline{\overline{{\mathord{\buildrel{\lower1pt\hbox{$\scriptscriptstyle\smile$}}\over{\mathfrak{W}}}}_{{\mathfrak{L}}_{k+1}}}}}}}}^{l}\right)\right)}^{\Lambda }\right)}^{\frac{1}{\Lambda }}},{e}^{-{\left(\overline{\overline{{\Xi }_{k+1}}}{\left(-\mathrm{log}\left({\overline{\overline{{\eta }_{\overline{\overline{{\mathord{\buildrel{\lower1pt\hbox{$\scriptscriptstyle\smile$}}\over{\mathfrak{W}}}}_{{\mathfrak{L}}_{k+1}}}}}}}}^{u}\right)\right)}^{\Lambda }\right)}^{\frac{1}{\Lambda }}}\right]\end{array}\right)$$

$$=\left(\begin{array}{c}\left[1-{e}^{-{\left(\sum_{\tau =1}^{k+1}\overline{\overline{{\Xi }_{\tau }}}{\left(-\mathrm{log}\left(1-{\overline{\overline{{\zeta }_{\overline{\overline{{\mathord{\buildrel{\lower1pt\hbox{$\scriptscriptstyle\smile$}}\over{\mathfrak{W}}}}_{{\mathfrak{L}}_{\tau }}}}}}}}^{l}\right)\right)}^{\Lambda }\right)}^{\frac{1}{\Lambda }}},1-{e}^{-{\left(\sum_{\tau =1}^{k+1}\overline{\overline{{\Xi }_{\tau }}}{\left(-\mathrm{log}\left(1-{\overline{\overline{{\zeta }_{\overline{\overline{{\mathord{\buildrel{\lower1pt\hbox{$\scriptscriptstyle\smile$}}\over{\mathfrak{W}}}}_{{\mathfrak{L}}_{\tau }}}}}}}}^{u}\right)\right)}^{\Lambda }\right)}^{\frac{1}{\Lambda }}}\right],\\ \left[{e}^{-{\left(\sum_{\tau =1}^{k+1}\overline{\overline{{\Xi }_{\tau }}}{\left(-\mathrm{log}\left({\overline{\overline{{\eta }_{\overline{\overline{{\mathord{\buildrel{\lower1pt\hbox{$\scriptscriptstyle\smile$}}\over{\mathfrak{W}}}}_{{\mathfrak{L}}_{\tau }}}}}}}}^{l}\right)\right)}^{\Lambda }\right)}^{\frac{1}{\Lambda }}},{e}^{-{\left(\sum_{\tau =1}^{k+1}\overline{\overline{{\Xi }_{\tau }}}{\left(-\mathrm{log}\left({\overline{\overline{{\eta }_{\overline{\overline{{\mathord{\buildrel{\lower1pt\hbox{$\scriptscriptstyle\smile$}}\over{\mathfrak{W}}}}_{{\mathfrak{L}}_{\tau }}}}}}}}^{u}\right)\right)}^{\Lambda }\right)}^{\frac{1}{\Lambda }}}\right]\end{array}\right)$$

Therefore, when ${\rm Z}=k+1$, (12) is also kept.

In a word, (12) is right.

### Property 1 

(Idempotency) For a group of IVA-IFNs $$\overline{\overline{{\mathord{\buildrel{\lower1pt\hbox{$\scriptscriptstyle\smile$}}\over{\mathfrak{W}}}}_{{\mathfrak{L}}_{\tau }}}}=\left(\left[{\overline{\overline{{\zeta }_{\overline{\overline{{\mathord{\buildrel{\lower1pt\hbox{$\scriptscriptstyle\smile$}}\over{\mathfrak{W}}}}_{{\mathfrak{L}}_{\tau }}}}}}}}^{l},{\overline{\overline{{\zeta }_{\overline{\overline{{\mathord{\buildrel{\lower1pt\hbox{$\scriptscriptstyle\smile$}}\over{\mathfrak{W}}}}_{{\mathfrak{L}}_{\tau }}}}}}}}^{u}\right],\left[{\overline{\overline{{\eta }_{\overline{\overline{{\mathord{\buildrel{\lower1pt\hbox{$\scriptscriptstyle\smile$}}\over{\mathfrak{W}}}}_{{\mathfrak{L}}_{\tau }}}}}}}}^{l},{\overline{\overline{{\eta }_{\overline{\overline{{\mathord{\buildrel{\lower1pt\hbox{$\scriptscriptstyle\smile$}}\over{\mathfrak{W}}}}_{{\mathfrak{L}}_{\tau }}}}}}}}^{u}\right]\right),\tau =\mathrm{1,2},\dots ,{\rm Z}$$, If $$\overline{\overline{{\mathord{\buildrel{\lower1pt\hbox{$\scriptscriptstyle\smile$}}\over{\mathfrak{W}}}}_{{\mathfrak{L}}_{\tau }}}}=\overline{\overline{{\mathord{\buildrel{\lower1pt\hbox{$\scriptscriptstyle\smile$}}\over{\mathfrak{W}}}}_{\mathfrak{L}}}}=\left(\left[{\overline{\overline{{\zeta }_{\overline{\overline{{\mathord{\buildrel{\lower1pt\hbox{$\scriptscriptstyle\smile$}}\over{\mathfrak{W}}}}_{\mathfrak{L}}}}}}}}^{l},{\overline{\overline{{\zeta }_{\overline{\overline{{\mathord{\buildrel{\lower1pt\hbox{$\scriptscriptstyle\smile$}}\over{\mathfrak{W}}}}_{\mathfrak{L}}}}}}}}^{u}\right],\left[{\overline{\overline{{\eta }_{\overline{\overline{{\mathord{\buildrel{\lower1pt\hbox{$\scriptscriptstyle\smile$}}\over{\mathfrak{W}}}}_{\mathfrak{L}}}}}}}}^{l},{\overline{\overline{{\eta }_{\overline{\overline{{\mathord{\buildrel{\lower1pt\hbox{$\scriptscriptstyle\smile$}}\over{\mathfrak{W}}}}_{\mathfrak{L}}}}}}}}^{u}\right]\right)$$, then

13 $$IVA-IFAAPA\left(\overline{\overline{{\mathord{\buildrel{\lower1pt\hbox{$\scriptscriptstyle\smile$}}\over{\mathfrak{W}}}}_{{\mathfrak{L}}_{1}}}},\overline{\overline{{\mathord{\buildrel{\lower1pt\hbox{$\scriptscriptstyle\smile$}}\over{\mathfrak{W}}}}_{{\mathfrak{L}}_{2}}}},\dots ,\overline{\overline{{\mathord{\buildrel{\lower1pt\hbox{$\scriptscriptstyle\smile$}}\over{\mathfrak{W}}}}_{{\mathfrak{L}}_{\rm Z}}}}\right)=\overline{\overline{{\mathord{\buildrel{\lower1pt\hbox{$\scriptscriptstyle\smile$}}\over{\mathfrak{W}}}}_{\mathfrak{L}}}}$$

### Proof

For a group of IVA-IFNs $$\overline{\overline{{\mathord{\buildrel{\lower1pt\hbox{$\scriptscriptstyle\smile$}}\over{\mathfrak{W}}}}_{{\mathfrak{L}}_{\tau }}}}=\left(\left[{\overline{\overline{{\zeta }_{\overline{\overline{{\mathord{\buildrel{\lower1pt\hbox{$\scriptscriptstyle\smile$}}\over{\mathfrak{W}}}}_{{\mathfrak{L}}_{\tau }}}}}}}}^{l},{\overline{\overline{{\zeta }_{\overline{\overline{{\mathord{\buildrel{\lower1pt\hbox{$\scriptscriptstyle\smile$}}\over{\mathfrak{W}}}}_{{\mathfrak{L}}_{\tau }}}}}}}}^{u}\right],\left[{\overline{\overline{{\eta }_{\overline{\overline{{\mathord{\buildrel{\lower1pt\hbox{$\scriptscriptstyle\smile$}}\over{\mathfrak{W}}}}_{{\mathfrak{L}}_{\tau }}}}}}}}^{l},{\overline{\overline{{\eta }_{\overline{\overline{{\mathord{\buildrel{\lower1pt\hbox{$\scriptscriptstyle\smile$}}\over{\mathfrak{W}}}}_{{\mathfrak{L}}_{\tau }}}}}}}}^{u}\right]\right),\tau =\mathrm{1,2},\dots ,{\rm Z},$$

If $$\overline{\overline{{\mathord{\buildrel{\lower1pt\hbox{$\scriptscriptstyle\smile$}}\over{\mathfrak{W}}}}_{{\mathfrak{L}}_{\tau }}}}=\overline{\overline{{\mathord{\buildrel{\lower1pt\hbox{$\scriptscriptstyle\smile$}}\over{\mathfrak{W}}}}_{\mathfrak{L}}}}=\left(\left[{\overline{\overline{{\zeta }_{\overline{\overline{{\mathord{\buildrel{\lower1pt\hbox{$\scriptscriptstyle\smile$}}\over{\mathfrak{W}}}}_{\mathfrak{L}}}}}}}}^{l},{\overline{\overline{{\zeta }_{\overline{\overline{{\mathord{\buildrel{\lower1pt\hbox{$\scriptscriptstyle\smile$}}\over{\mathfrak{W}}}}_{\mathfrak{L}}}}}}}}^{u}\right],\left[{\overline{\overline{{\eta }_{\overline{\overline{{\mathord{\buildrel{\lower1pt\hbox{$\scriptscriptstyle\smile$}}\over{\mathfrak{W}}}}_{\mathfrak{L}}}}}}}}^{l},{\overline{\overline{{\eta }_{\overline{\overline{{\mathord{\buildrel{\lower1pt\hbox{$\scriptscriptstyle\smile$}}\over{\mathfrak{W}}}}_{\mathfrak{L}}}}}}}}^{u}\right]\right),$$ then

$$IVA-IFAAPA\left(\overline{\overline{{\mathord{\buildrel{\lower1pt\hbox{$\scriptscriptstyle\smile$}}\over{\mathfrak{W}}}}_{{\mathfrak{L}}_{1}}}},\overline{\overline{{\mathord{\buildrel{\lower1pt\hbox{$\scriptscriptstyle\smile$}}\over{\mathfrak{W}}}}_{{\mathfrak{L}}_{2}}}},\dots ,\overline{\overline{{\mathord{\buildrel{\lower1pt\hbox{$\scriptscriptstyle\smile$}}\over{\mathfrak{W}}}}_{{\mathfrak{L}}_{\rm Z}}}}\right)=\left(\begin{array}{c}\left[1-{e}^{-{\left(\sum_{\tau =1}^{\rm Z}\overline{\overline{{\Xi }_{\tau }}}{\left(-\mathrm{log}\left(1-{\overline{\overline{{\zeta }_{\overline{\overline{{\mathord{\buildrel{\lower1pt\hbox{$\scriptscriptstyle\smile$}}\over{\mathfrak{W}}}}_{{\mathfrak{L}}_{\tau }}}}}}}}^{l}\right)\right)}^{\Lambda }\right)}^{\frac{1}{\Lambda }}},1-{e}^{-{\left(\sum_{\tau =1}^{\rm Z}\overline{\overline{{\Xi }_{\tau }}}{\left(-\mathrm{log}\left(1-{\overline{\overline{{\zeta }_{\overline{\overline{{\mathord{\buildrel{\lower1pt\hbox{$\scriptscriptstyle\smile$}}\over{\mathfrak{W}}}}_{{\mathfrak{L}}_{\tau }}}}}}}}^{u}\right)\right)}^{\Lambda }\right)}^{\frac{1}{\Lambda }}}\right],\\ \left[{e}^{-{\left(\sum_{\tau =1}^{\rm Z}\overline{\overline{{\Xi }_{\tau }}}{\left(-\mathrm{log}\left({\overline{\overline{{\eta }_{\overline{\overline{{\mathord{\buildrel{\lower1pt\hbox{$\scriptscriptstyle\smile$}}\over{\mathfrak{W}}}}_{{\mathfrak{L}}_{\tau }}}}}}}}^{l}\right)\right)}^{\Lambda }\right)}^{\frac{1}{\Lambda }}},{e}^{-{\left(\sum_{\tau =1}^{\rm Z}\overline{\overline{{\Xi }_{\tau }}}{\left(-\mathrm{log}\left({\overline{\overline{{\eta }_{\overline{\overline{{\mathord{\buildrel{\lower1pt\hbox{$\scriptscriptstyle\smile$}}\over{\mathfrak{W}}}}_{{\mathfrak{L}}_{\tau }}}}}}}}^{u}\right)\right)}^{\Lambda }\right)}^{\frac{1}{\Lambda }}}\right]\end{array}\right)$$

$$=\left(\begin{array}{c}\left[1-{e}^{-{\left(\sum_{\tau =1}^{\rm Z}\overline{\overline{{\Xi }_{\tau }}}{\left(-\mathrm{log}\left(1-{\overline{\overline{{\zeta }_{\overline{\overline{{\mathord{\buildrel{\lower1pt\hbox{$\scriptscriptstyle\smile$}}\over{\mathfrak{W}}}}_{\mathfrak{L}}}}}}}}^{l}\right)\right)}^{\Lambda }\right)}^{\frac{1}{\Lambda }}},1-{e}^{-{\left(\sum_{\tau =1}^{\rm Z}\overline{\overline{{\Xi }_{\tau }}}{\left(-\mathrm{log}\left(1-{\overline{\overline{{\zeta }_{\overline{\overline{{\mathord{\buildrel{\lower1pt\hbox{$\scriptscriptstyle\smile$}}\over{\mathfrak{W}}}}_{\mathfrak{L}}}}}}}}^{u}\right)\right)}^{\Lambda }\right)}^{\frac{1}{\Lambda }}}\right],\\ \left[{e}^{-{\left(\sum_{\tau =1}^{\rm Z}\overline{\overline{{\Xi }_{\tau }}}{\left(-\mathrm{log}\left({\overline{\overline{{\eta }_{\overline{\overline{{\mathord{\buildrel{\lower1pt\hbox{$\scriptscriptstyle\smile$}}\over{\mathfrak{W}}}}_{\mathfrak{L}}}}}}}}^{l}\right)\right)}^{\Lambda }\right)}^{\frac{1}{\Lambda }}},{e}^{-{\left(\sum_{\tau =1}^{\rm Z}\overline{\overline{{\Xi }_{\tau }}}{\left(-\mathrm{log}\left({\overline{\overline{{\eta }_{\overline{\overline{{\mathord{\buildrel{\lower1pt\hbox{$\scriptscriptstyle\smile$}}\over{\mathfrak{W}}}}_{\mathfrak{L}}}}}}}}^{u}\right)\right)}^{\Lambda }\right)}^{\frac{1}{\Lambda }}}\right]\end{array}\right)$$

$$=\left(\begin{array}{c}\left[1-{e}^{-{\left({\left(-\mathrm{log}\left(1-{\overline{\overline{{\zeta }_{\overline{\overline{{\mathord{\buildrel{\lower1pt\hbox{$\scriptscriptstyle\smile$}}\over{\mathfrak{W}}}}_{\mathfrak{L}}}}}}}}^{l}\right)\right)}^{\Lambda }\right)}^{\frac{1}{\Lambda }}},1-{e}^{-{\left({\left(-\mathrm{log}\left(1-{\overline{\overline{{\zeta }_{\overline{\overline{{\mathord{\buildrel{\lower1pt\hbox{$\scriptscriptstyle\smile$}}\over{\mathfrak{W}}}}_{\mathfrak{L}}}}}}}}^{u}\right)\right)}^{\Lambda }\right)}^{\frac{1}{\Lambda }}}\right],\\ \left[{e}^{-{\left({\left(-\mathrm{log}\left({\overline{\overline{{\eta }_{\overline{\overline{{\mathord{\buildrel{\lower1pt\hbox{$\scriptscriptstyle\smile$}}\over{\mathfrak{W}}}}_{\mathfrak{L}}}}}}}}^{l}\right)\right)}^{\Lambda }\right)}^{\frac{1}{\Lambda }}},{e}^{-{\left({\left(-\mathrm{log}\left({\overline{\overline{{\eta }_{\overline{\overline{{\mathord{\buildrel{\lower1pt\hbox{$\scriptscriptstyle\smile$}}\over{\mathfrak{W}}}}_{\mathfrak{L}}}}}}}}^{u}\right)\right)}^{\Lambda }\right)}^{\frac{1}{\Lambda }}}\right]\end{array}\right),\sum_{\tau =1}^{\rm Z}\overline{\overline{{\Xi }_{\tau }}}=1$$

$$=\left(\left[1-{e}^{\mathrm{log}\left(1-{\overline{\overline{{\zeta }_{\overline{\overline{{\mathord{\buildrel{\lower1pt\hbox{$\scriptscriptstyle\smile$}}\over{\mathfrak{W}}}}_{\mathfrak{L}}}}}}}}^{l}\right)},1-{e}^{\mathrm{log}\left(1-{\overline{\overline{{\zeta }_{\overline{\overline{{\mathord{\buildrel{\lower1pt\hbox{$\scriptscriptstyle\smile$}}\over{\mathfrak{W}}}}_{\mathfrak{L}}}}}}}}^{u}\right)}\right],\left[{e}^{\mathrm{log}\left({\overline{\overline{{\eta }_{\overline{\overline{{\mathord{\buildrel{\lower1pt\hbox{$\scriptscriptstyle\smile$}}\over{\mathfrak{W}}}}_{\mathfrak{L}}}}}}}}^{l}\right)},{e}^{\mathrm{log}\left({\overline{\overline{{\eta }_{\overline{\overline{{\mathord{\buildrel{\lower1pt\hbox{$\scriptscriptstyle\smile$}}\over{\mathfrak{W}}}}_{\mathfrak{L}}}}}}}}^{u}\right)}\right]\right)=\left(\left[{\overline{\overline{{\zeta }_{\overline{\overline{{\mathord{\buildrel{\lower1pt\hbox{$\scriptscriptstyle\smile$}}\over{\mathfrak{W}}}}_{\mathfrak{L}}}}}}}}^{l},{\overline{\overline{{\zeta }_{\overline{\overline{{\mathord{\buildrel{\lower1pt\hbox{$\scriptscriptstyle\smile$}}\over{\mathfrak{W}}}}_{\mathfrak{L}}}}}}}}^{u}\right],\left[{\overline{\overline{{\eta }_{\overline{\overline{{\mathord{\buildrel{\lower1pt\hbox{$\scriptscriptstyle\smile$}}\over{\mathfrak{W}}}}_{\mathfrak{L}}}}}}}}^{l},{\overline{\overline{{\eta }_{\overline{\overline{{\mathord{\buildrel{\lower1pt\hbox{$\scriptscriptstyle\smile$}}\over{\mathfrak{W}}}}_{\mathfrak{L}}}}}}}}^{u}\right]\right)=\overline{\overline{{\mathord{\buildrel{\lower1pt\hbox{$\scriptscriptstyle\smile$}}\over{\mathfrak{W}}}}_{\mathfrak{L}}}}.$$

### Property 2 

(Non-monotonicity) For a group of IVA-IFNs $$\overline{\overline{{\mathord{\buildrel{\lower1pt\hbox{$\scriptscriptstyle\smile$}}\over{\mathfrak{W}}}}_{{\mathfrak{L}}_{\tau }}}}=\left(\left[{\overline{\overline{{\zeta }_{\overline{\overline{{\mathord{\buildrel{\lower1pt\hbox{$\scriptscriptstyle\smile$}}\over{\mathfrak{W}}}}_{{\mathfrak{L}}_{\tau }}}}}}}}^{l},{\overline{\overline{{\zeta }_{\overline{\overline{{\mathord{\buildrel{\lower1pt\hbox{$\scriptscriptstyle\smile$}}\over{\mathfrak{W}}}}_{{\mathfrak{L}}_{\tau }}}}}}}}^{u}\right],\left[{\overline{\overline{{\eta }_{\overline{\overline{{\mathord{\buildrel{\lower1pt\hbox{$\scriptscriptstyle\smile$}}\over{\mathfrak{W}}}}_{{\mathfrak{L}}_{\tau }}}}}}}}^{l},{\overline{\overline{{\eta }_{\overline{\overline{{\mathord{\buildrel{\lower1pt\hbox{$\scriptscriptstyle\smile$}}\over{\mathfrak{W}}}}_{{\mathfrak{L}}_{\tau }}}}}}}}^{u}\right]\right),\tau =\mathrm{1,2},\dots ,{\rm Z},$$ If $$\overline{\overline{{\mathord{\buildrel{\lower1pt\hbox{$\scriptscriptstyle\smile$}}\over{\mathfrak{W}}}}_{{\mathfrak{L}}_{\tau }}}}\le {\overline{\overline{{\mathord{\buildrel{\lower1pt\hbox{$\scriptscriptstyle\smile$}}\over{\mathfrak{W}}}}_{{\mathfrak{L}}_{\tau }}}}}^{^{\prime}}=\left(\left[{{\overline{\overline{{\zeta }_{\overline{\overline{{\mathord{\buildrel{\lower1pt\hbox{$\scriptscriptstyle\smile$}}\over{\mathfrak{W}}}}_{{\mathfrak{L}}_{\tau }}}}}}}}^{^{\prime}}}^{l},{{\overline{\overline{{\zeta }_{\overline{\overline{{\mathord{\buildrel{\lower1pt\hbox{$\scriptscriptstyle\smile$}}\over{\mathfrak{W}}}}_{{\mathfrak{L}}_{\tau }}}}}}}}^{^{\prime}}}^{u}\right],\left[{{\overline{\overline{{\eta }_{\overline{\overline{{\mathord{\buildrel{\lower1pt\hbox{$\scriptscriptstyle\smile$}}\over{\mathfrak{W}}}}_{{\mathfrak{L}}_{\tau }}}}}}}}^{^{\prime}}}^{l},{{\overline{\overline{{\eta }_{\overline{\overline{{\mathord{\buildrel{\lower1pt\hbox{$\scriptscriptstyle\smile$}}\over{\mathfrak{W}}}}_{{\mathfrak{L}}_{\tau }}}}}}}}^{^{\prime}}}^{u}\right]\right),$$ then

14 $$IVA-IFAAPA\left(\overline{\overline{{\mathord{\buildrel{\lower1pt\hbox{$\scriptscriptstyle\smile$}}\over{\mathfrak{W}}}}_{{\mathfrak{L}}_{1}}}},\overline{\overline{{\mathord{\buildrel{\lower1pt\hbox{$\scriptscriptstyle\smile$}}\over{\mathfrak{W}}}}_{{\mathfrak{L}}_{2}}}},\dots ,\overline{\overline{{\mathord{\buildrel{\lower1pt\hbox{$\scriptscriptstyle\smile$}}\over{\mathfrak{W}}}}_{{\mathfrak{L}}_{\rm Z}}}}\right)\nleqq IVA-IFAAPA\left({\overline{\overline{{\mathord{\buildrel{\lower1pt\hbox{$\scriptscriptstyle\smile$}}\over{\mathfrak{W}}}}_{{\mathfrak{L}}_{1}}}}}^{^{\prime}},{\overline{\overline{{\mathord{\buildrel{\lower1pt\hbox{$\scriptscriptstyle\smile$}}\over{\mathfrak{W}}}}_{{\mathfrak{L}}_{2}}}}}^{^{\prime}},\dots ,{\overline{\overline{{\mathord{\buildrel{\lower1pt\hbox{$\scriptscriptstyle\smile$}}\over{\mathfrak{W}}}}_{{\mathfrak{L}}_{\rm Z}}}}}^{^{\prime}}\right)$$

Here, we give only some counterexamples to show that Eq. (14) is held correctly. For this, we use a few IVA-IFNs such as $$\overline{\overline{{\mathord{\buildrel{\lower1pt\hbox{$\scriptscriptstyle\smile$}}\over{\mathfrak{W}}}}_{{\mathfrak{L}}_{1}}}}=\left(\left[\mathrm{0.2,0.21}\right],\left[\mathrm{0.2,0.21}\right]\right),\overline{\overline{{\mathord{\buildrel{\lower1pt\hbox{$\scriptscriptstyle\smile$}}\over{\mathfrak{W}}}}_{{\mathfrak{L}}_{2}}}}=\left(\left[\mathrm{0.4,0.41}\right],\left[\mathrm{0.4,0.4}1\right]\right),\overline{\overline{{\mathord{\buildrel{\lower1pt\hbox{$\scriptscriptstyle\smile$}}\over{\mathfrak{W}}}}_{{\mathfrak{L}}_{3}}}}=\left(\left[\mathrm{0.1,0.11}\right],\left[\mathrm{0.88,0.89}\right]\right)$$ and $${\overline{\overline{{\mathord{\buildrel{\lower1pt\hbox{$\scriptscriptstyle\smile$}}\over{\mathfrak{W}}}}_{{\mathfrak{L}}_{1}}}}}^{^{\prime}}=\left(\left[\mathrm{0.2,0.21}\right],\left[\mathrm{0.2,0.21}\right]\right),{\overline{\overline{{\mathord{\buildrel{\lower1pt\hbox{$\scriptscriptstyle\smile$}}\over{\mathfrak{W}}}}_{{\mathfrak{L}}_{2}}}}}^{^{\prime}}=\left(\left[\mathrm{0.4,0.41}\right],\left[\mathrm{0.4,0.41}\right]\right),{\overline{\overline{{\mathord{\buildrel{\lower1pt\hbox{$\scriptscriptstyle\smile$}}\over{\mathfrak{W}}}}_{{\mathfrak{L}}_{3}}}}}^{^{\prime}}=\left(\left[\mathrm{0.11,0.12}\right],\left[\mathrm{0.11,0.12}\right]\right)$$ with $$\Lambda =2$$. We get the following results:

$$Dis\left(\overline{\overline{{\mathord{\buildrel{\lower1pt\hbox{$\scriptscriptstyle\smile$}}\over{\mathfrak{W}}}}_{{\mathfrak{L}}_{1}}}},\overline{\overline{{\mathord{\buildrel{\lower1pt\hbox{$\scriptscriptstyle\smile$}}\over{\mathfrak{W}}}}_{{\mathfrak{L}}_{2}}}}\right)=0.2$$

$$Dis\left(\overline{\overline{{\mathord{\buildrel{\lower1pt\hbox{$\scriptscriptstyle\smile$}}\over{\mathfrak{W}}}}_{{\mathfrak{L}}_{1}}}},\overline{\overline{{\mathord{\buildrel{\lower1pt\hbox{$\scriptscriptstyle\smile$}}\over{\mathfrak{W}}}}_{{\mathfrak{L}}_{3}}}}\right)=0.39;Dis\left(\overline{\overline{{\mathord{\buildrel{\lower1pt\hbox{$\scriptscriptstyle\smile$}}\over{\mathfrak{W}}}}_{{\mathfrak{L}}_{2}}}},\overline{\overline{{\mathord{\buildrel{\lower1pt\hbox{$\scriptscriptstyle\smile$}}\over{\mathfrak{W}}}}_{{\mathfrak{L}}_{3}}}}\right)=0.39$$

$$Sup\left(\overline{\overline{{\mathord{\buildrel{\lower1pt\hbox{$\scriptscriptstyle\smile$}}\over{\mathfrak{W}}}}_{{\mathfrak{L}}_{1}}}},\overline{\overline{{\mathord{\buildrel{\lower1pt\hbox{$\scriptscriptstyle\smile$}}\over{\mathfrak{W}}}}_{{\mathfrak{L}}_{2}}}}\right)=1-Dis\left(\overline{\overline{{\mathord{\buildrel{\lower1pt\hbox{$\scriptscriptstyle\smile$}}\over{\mathfrak{W}}}}_{{\mathfrak{L}}_{1}}}},\overline{\overline{{\mathord{\buildrel{\lower1pt\hbox{$\scriptscriptstyle\smile$}}\over{\mathfrak{W}}}}_{{\mathfrak{L}}_{2}}}}\right)=1-0.2=0.8$$

$$Sup\left(\overline{\overline{{\mathord{\buildrel{\lower1pt\hbox{$\scriptscriptstyle\smile$}}\over{\mathfrak{W}}}}_{{\mathfrak{L}}_{1}}}},\overline{\overline{{\mathord{\buildrel{\lower1pt\hbox{$\scriptscriptstyle\smile$}}\over{\mathfrak{W}}}}_{{\mathfrak{L}}_{3}}}}\right)=0.61;Sup\left(\overline{\overline{{\mathord{\buildrel{\lower1pt\hbox{$\scriptscriptstyle\smile$}}\over{\mathfrak{W}}}}_{{\mathfrak{L}}_{2}}}},\overline{\overline{{\mathord{\buildrel{\lower1pt\hbox{$\scriptscriptstyle\smile$}}\over{\mathfrak{W}}}}_{{\mathfrak{L}}_{3}}}}\right)=0.61$$

Thus,

$$\overline{\overline{\mathfrak{I}}}\left(\overline{\overline{{\mathord{\buildrel{\lower1pt\hbox{$\scriptscriptstyle\smile$}}\over{\mathfrak{W}}}}_{{\mathfrak{L}}_{1}}}}\right)=\sum_{\begin{array}{c}k=1,\\ k\ne 1\end{array}}^{\rm Z}Sup\left(\overline{\overline{{\mathord{\buildrel{\lower1pt\hbox{$\scriptscriptstyle\smile$}}\over{\mathfrak{W}}}}_{{\mathfrak{L}}_{1}}}},\overline{\overline{{\mathord{\buildrel{\lower1pt\hbox{$\scriptscriptstyle\smile$}}\over{\mathfrak{W}}}}_{{\mathfrak{L}}_{k}}}}\right)=Sup\left(\overline{\overline{{\mathord{\buildrel{\lower1pt\hbox{$\scriptscriptstyle\smile$}}\over{\mathfrak{W}}}}_{{\mathfrak{L}}_{1}}}},\overline{\overline{{\mathord{\buildrel{\lower1pt\hbox{$\scriptscriptstyle\smile$}}\over{\mathfrak{W}}}}_{{\mathfrak{L}}_{2}}}}\right)+Sup\left(\overline{\overline{{\mathord{\buildrel{\lower1pt\hbox{$\scriptscriptstyle\smile$}}\over{\mathfrak{W}}}}_{{\mathfrak{L}}_{1}}}},\overline{\overline{{\mathord{\buildrel{\lower1pt\hbox{$\scriptscriptstyle\smile$}}\over{\mathfrak{W}}}}_{{\mathfrak{L}}_{3}}}}\right)=1.41$$

$$\overline{\overline{\mathfrak{I}}}\left(\overline{\overline{{\mathord{\buildrel{\lower1pt\hbox{$\scriptscriptstyle\smile$}}\over{\mathfrak{W}}}}_{{\mathfrak{L}}_{2}}}}\right)=1.41; \overline{\overline{\mathfrak{I}}}\left(\overline{\overline{{\mathord{\buildrel{\lower1pt\hbox{$\scriptscriptstyle\smile$}}\over{\mathfrak{W}}}}_{{\mathfrak{L}}_{3}}}}\right)=1.22$$

$$\sum_{\tau =1}^{\rm Z}\left(1+\overline{\overline{\mathfrak{I}}}\left(\overline{\overline{{\mathord{\buildrel{\lower1pt\hbox{$\scriptscriptstyle\smile$}}\over{\mathfrak{W}}}}_{{\mathfrak{L}}_{\tau }}}}\right)\right)=7.04$$

Thus,

$$\overline{\overline{{\Xi }_{1}}}=\frac{\left(1+\overline{\overline{\mathfrak{I}}}\left(\overline{\overline{{\mathord{\buildrel{\lower1pt\hbox{$\scriptscriptstyle\smile$}}\over{\mathfrak{W}}}}_{{\mathfrak{L}}_{1}}}}\right)\right)}{\sum_{\tau =1}^{3}\left(1+\overline{\overline{\mathfrak{I}}}\left(\overline{\overline{{\mathord{\buildrel{\lower1pt\hbox{$\scriptscriptstyle\smile$}}\over{\mathfrak{W}}}}_{{\mathfrak{L}}_{\tau }}}}\right)\right)}=\frac{1+1.41}{7.04}=0.34233$$

$$\overline{\overline{{\Xi }_{2}}}=0.34233;\overline{\overline{{\Xi }_{3}}}=0.31534.$$

Hence, by Eq. (12), we have

$$IVA-IFAAPA\left(\overline{\overline{{\mathord{\buildrel{\lower1pt\hbox{$\scriptscriptstyle\smile$}}\over{\mathfrak{W}}}}_{{\mathfrak{L}}_{1}}}},\overline{\overline{{\mathord{\buildrel{\lower1pt\hbox{$\scriptscriptstyle\smile$}}\over{\mathfrak{W}}}}_{{\mathfrak{L}}_{2}}}},\overline{\overline{{\mathord{\buildrel{\lower1pt\hbox{$\scriptscriptstyle\smile$}}\over{\mathfrak{W}}}}_{{\mathfrak{L}}_{3}}}}\right)=\left(\left[\mathrm{0.13408,0.13892}\right],\left[\mathrm{0.62399,0.6328}\right]\right)$$

In the same way, we get

$$IVA-IFAAPA\left({\overline{\overline{{\mathord{\buildrel{\lower1pt\hbox{$\scriptscriptstyle\smile$}}\over{\mathfrak{W}}}}_{{\mathfrak{L}}_{1}}}}}^{^{\prime}},{\overline{\overline{{\mathord{\buildrel{\lower1pt\hbox{$\scriptscriptstyle\smile$}}\over{\mathfrak{W}}}}_{{\mathfrak{L}}_{2}}}}}^{^{\prime}},{\overline{\overline{{\mathord{\buildrel{\lower1pt\hbox{$\scriptscriptstyle\smile$}}\over{\mathfrak{W}}}}_{{\mathfrak{L}}_{3}}}}}^{^{\prime}}\right)=\left(\left[\mathrm{0.13144,0.13628}\right],\left[\mathrm{0.48398,0.49653}\right]\right)$$

From the above two information, we get

$$IVA-IFAAPA\left(\overline{\overline{{\mathord{\buildrel{\lower1pt\hbox{$\scriptscriptstyle\smile$}}\over{\mathfrak{W}}}}_{{\mathfrak{L}}_{1}}}},\overline{\overline{{\mathord{\buildrel{\lower1pt\hbox{$\scriptscriptstyle\smile$}}\over{\mathfrak{W}}}}_{{\mathfrak{L}}_{2}}}},\dots ,\overline{\overline{{\mathord{\buildrel{\lower1pt\hbox{$\scriptscriptstyle\smile$}}\over{\mathfrak{W}}}}_{{\mathfrak{L}}_{\rm Z}}}}\right)\nleqq IVA-IFAAPA\left({\overline{\overline{{\mathord{\buildrel{\lower1pt\hbox{$\scriptscriptstyle\smile$}}\over{\mathfrak{W}}}}_{{\mathfrak{L}}_{1}}}}}^{^{\prime}},{\overline{\overline{{\mathord{\buildrel{\lower1pt\hbox{$\scriptscriptstyle\smile$}}\over{\mathfrak{W}}}}_{{\mathfrak{L}}_{2}}}}}^{^{\prime}},\dots ,{\overline{\overline{{\mathord{\buildrel{\lower1pt\hbox{$\scriptscriptstyle\smile$}}\over{\mathfrak{W}}}}_{{\mathfrak{L}}_{\rm Z}}}}}^{^{\prime}}\right)$$

Noticing that the monotonicity has been neglected. Further, we give the boundedness.

### Property 3 

(Boundedness) For a group of IVA-IFNs $$\overline{\overline{{\mathord{\buildrel{\lower1pt\hbox{$\scriptscriptstyle\smile$}}\over{\mathfrak{W}}}}_{{\mathfrak{L}}_{\tau }}}}=\left(\left[{\overline{\overline{{\zeta }_{\overline{\overline{{\mathord{\buildrel{\lower1pt\hbox{$\scriptscriptstyle\smile$}}\over{\mathfrak{W}}}}_{{\mathfrak{L}}_{\tau }}}}}}}}^{l},{\overline{\overline{{\zeta }_{\overline{\overline{{\mathord{\buildrel{\lower1pt\hbox{$\scriptscriptstyle\smile$}}\over{\mathfrak{W}}}}_{{\mathfrak{L}}_{\tau }}}}}}}}^{u}\right],\left[{\overline{\overline{{\eta }_{\overline{\overline{{\mathord{\buildrel{\lower1pt\hbox{$\scriptscriptstyle\smile$}}\over{\mathfrak{W}}}}_{{\mathfrak{L}}_{\tau }}}}}}}}^{l},{\overline{\overline{{\eta }_{\overline{\overline{{\mathord{\buildrel{\lower1pt\hbox{$\scriptscriptstyle\smile$}}\over{\mathfrak{W}}}}_{{\mathfrak{L}}_{\tau }}}}}}}}^{u}\right]\right),\tau =\mathrm{1,2},\dots ,{\rm Z},$$ If $${\overline{\overline{{\mathord{\buildrel{\lower1pt\hbox{$\scriptscriptstyle\smile$}}\over{\mathfrak{W}}}}_{{\mathfrak{L}}_{\tau }}}}}^{-}=\left(\left[\underset{\tau }{\mathrm{min}}{\overline{\overline{{\zeta }_{\overline{\overline{{\mathord{\buildrel{\lower1pt\hbox{$\scriptscriptstyle\smile$}}\over{\mathfrak{W}}}}_{{\mathfrak{L}}_{\tau }}}}}}}}^{l},\underset{\tau }{\mathrm{min}}{\overline{\overline{{\zeta }_{\overline{\overline{{\mathord{\buildrel{\lower1pt\hbox{$\scriptscriptstyle\smile$}}\over{\mathfrak{W}}}}_{{\mathfrak{L}}_{\tau }}}}}}}}^{u}\right],\left[\underset{\tau }{\mathrm{max}}{\overline{\overline{{\eta }_{\overline{\overline{{\mathord{\buildrel{\lower1pt\hbox{$\scriptscriptstyle\smile$}}\over{\mathfrak{W}}}}_{{\mathfrak{L}}_{\tau }}}}}}}}^{l},\underset{\tau }{\mathrm{max}}{\overline{\overline{{\eta }_{\overline{\overline{{\mathord{\buildrel{\lower1pt\hbox{$\scriptscriptstyle\smile$}}\over{\mathfrak{W}}}}_{{\mathfrak{L}}_{\tau }}}}}}}}^{u}\right]\right)$$ and $${\overline{\overline{{\mathord{\buildrel{\lower1pt\hbox{$\scriptscriptstyle\smile$}}\over{\mathfrak{W}}}}_{{\mathfrak{L}}_{\tau }}}}}^{+}=\left(\left[\underset{\tau }{\mathrm{max}}{\overline{\overline{{\zeta }_{\overline{\overline{{\mathord{\buildrel{\lower1pt\hbox{$\scriptscriptstyle\smile$}}\over{\mathfrak{W}}}}_{{\mathfrak{L}}_{\tau }}}}}}}}^{l},\underset{\tau }{\mathrm{max}}{\overline{\overline{{\zeta }_{\overline{\overline{{\mathord{\buildrel{\lower1pt\hbox{$\scriptscriptstyle\smile$}}\over{\mathfrak{W}}}}_{{\mathfrak{L}}_{\tau }}}}}}}}^{u}\right],\left[\underset{\tau }{\mathrm{min}}{\overline{\overline{{\eta }_{\overline{\overline{{\mathord{\buildrel{\lower1pt\hbox{$\scriptscriptstyle\smile$}}\over{\mathfrak{W}}}}_{{\mathfrak{L}}_{\tau }}}}}}}}^{l},\underset{\tau }{\mathrm{min}}{\overline{\overline{{\eta }_{\overline{\overline{{\mathord{\buildrel{\lower1pt\hbox{$\scriptscriptstyle\smile$}}\over{\mathfrak{W}}}}_{{\mathfrak{L}}_{\tau }}}}}}}}^{u}\right]\right),$$ then

$${\overline{\overline{{\mathord{\buildrel{\lower1pt\hbox{$\scriptscriptstyle\smile$}}\over{\mathfrak{W}}}}_{{\mathfrak{L}}_{\tau }}}}}^{-}\le IVA-IFAAPA\left(\overline{\overline{{\mathord{\buildrel{\lower1pt\hbox{$\scriptscriptstyle\smile$}}\over{\mathfrak{W}}}}_{{\mathfrak{L}}_{1}}}},\overline{\overline{{\mathord{\buildrel{\lower1pt\hbox{$\scriptscriptstyle\smile$}}\over{\mathfrak{W}}}}_{{\mathfrak{L}}_{2}}}},\dots ,\overline{\overline{{\mathord{\buildrel{\lower1pt\hbox{$\scriptscriptstyle\smile$}}\over{\mathfrak{W}}}}_{{\mathfrak{L}}_{\rm Z}}}}\right)\le {\overline{\overline{{\mathord{\buildrel{\lower1pt\hbox{$\scriptscriptstyle\smile$}}\over{\mathfrak{W}}}}_{{\mathfrak{L}}_{\tau }}}}}^{+}$$

### Definition 6

For a group of IVA-IFNs $$\overline{\overline{{\mathord{\buildrel{\lower1pt\hbox{$\scriptscriptstyle\smile$}}\over{\mathfrak{W}}}}_{{\mathfrak{L}}_{\tau }}}}=\left(\left[{\overline{\overline{{\zeta }_{\overline{\overline{{\mathord{\buildrel{\lower1pt\hbox{$\scriptscriptstyle\smile$}}\over{\mathfrak{W}}}}_{{\mathfrak{L}}_{\tau }}}}}}}}^{l},{\overline{\overline{{\zeta }_{\overline{\overline{{\mathord{\buildrel{\lower1pt\hbox{$\scriptscriptstyle\smile$}}\over{\mathfrak{W}}}}_{{\mathfrak{L}}_{\tau }}}}}}}}^{u}\right],\left[{\overline{\overline{{\eta }_{\overline{\overline{{\mathord{\buildrel{\lower1pt\hbox{$\scriptscriptstyle\smile$}}\over{\mathfrak{W}}}}_{{\mathfrak{L}}_{\tau }}}}}}}}^{l},{\overline{\overline{{\eta }_{\overline{\overline{{\mathord{\buildrel{\lower1pt\hbox{$\scriptscriptstyle\smile$}}\over{\mathfrak{W}}}}_{{\mathfrak{L}}_{\tau }}}}}}}}^{u}\right]\right),\tau =\mathrm{1,2},\dots ,{\rm Z},$$ then an IVA-IFAAPOA operator is described as

15 $$IVA-IFAAPOA\left(\overline{\overline{{\mathord{\buildrel{\lower1pt\hbox{$\scriptscriptstyle\smile$}}\over{\mathfrak{W}}}}_{{\mathfrak{L}}_{1}}}},\overline{\overline{{\mathord{\buildrel{\lower1pt\hbox{$\scriptscriptstyle\smile$}}\over{\mathfrak{W}}}}_{{\mathfrak{L}}_{2}}}},\dots ,\overline{\overline{{\mathord{\buildrel{\lower1pt\hbox{$\scriptscriptstyle\smile$}}\over{\mathfrak{W}}}}_{{\mathfrak{L}}_{\rm Z}}}}\right)=\overline{\overline{{\Xi }_{1}}}\overline{\overline{{\mathord{\buildrel{\lower1pt\hbox{$\scriptscriptstyle\smile$}}\over{\mathfrak{W}}}}_{{\mathfrak{L}}_{0\left(1\right)}}}}\oplus \overline{\overline{{\Xi }_{2}}}\overline{\overline{{\mathord{\buildrel{\lower1pt\hbox{$\scriptscriptstyle\smile$}}\over{\mathfrak{W}}}}_{{\mathfrak{L}}_{0\left(2\right)}}}}\oplus \dots \oplus \overline{\overline{{\Xi }_{\rm Z}}}\overline{\overline{{\mathord{\buildrel{\lower1pt\hbox{$\scriptscriptstyle\smile$}}\over{\mathfrak{W}}}}_{{\mathfrak{L}}_{0\left({\rm Z}\right)}}}}={\oplus }_{\tau =1}^{\rm Z}\left(\overline{\overline{{\Xi }_{\tau }}}\overline{\overline{{\mathord{\buildrel{\lower1pt\hbox{$\scriptscriptstyle\smile$}}\over{\mathfrak{W}}}}_{{\mathfrak{L}}_{0\left(\tau \right)}}}}\right)$$

where $$0\left(\tau \right)\le 0\left(\tau -1\right)$$ represents the permutations and$$\overline{\overline{{\Xi }_{\tau }}}=\frac{\left(1+\overline{\overline{\mathfrak{I}}}\left(\overline{\overline{{\mathord{\buildrel{\lower1pt\hbox{$\scriptscriptstyle\smile$}}\over{\mathfrak{W}}}}_{{\mathfrak{L}}_{\tau }}}}\right)\right)}{\sum_{\tau =1}^{\rm Z}\left(1+\overline{\overline{\mathfrak{I}}}\left(\overline{\overline{{\mathord{\buildrel{\lower1pt\hbox{$\scriptscriptstyle\smile$}}\over{\mathfrak{W}}}}_{{\mathfrak{L}}_{\tau }}}}\right)\right)}$$. Further, the $$\overline{\overline{\mathfrak{I}}}\left(\overline{\overline{{\mathord{\buildrel{\lower1pt\hbox{$\scriptscriptstyle\smile$}}\over{\mathfrak{W}}}}_{{\mathfrak{L}}_{\tau }}}}\right)=\sum_{\begin{array}{c}s=1,\\ s\ne \tau \end{array}}^{\rm Z}Sup\left(\overline{\overline{{\mathord{\buildrel{\lower1pt\hbox{$\scriptscriptstyle\smile$}}\over{\mathfrak{W}}}}_{{\mathfrak{L}}_{\tau }}}},\overline{\overline{{\mathord{\buildrel{\lower1pt\hbox{$\scriptscriptstyle\smile$}}\over{\mathfrak{W}}}}_{{\mathfrak{L}}_{k}}}}\right),$$ which expresses the relationship between $$\overline{\overline{{\mathord{\buildrel{\lower1pt\hbox{$\scriptscriptstyle\smile$}}\over{\mathfrak{W}}}}_{{\mathfrak{L}}_{\tau }}}}$$ and $$\overline{\overline{{\mathord{\buildrel{\lower1pt\hbox{$\scriptscriptstyle\smile$}}\over{\mathfrak{W}}}}_{{\mathfrak{L}}_{k}}}},$$ where $$Sup\left(\overline{\overline{{\mathord{\buildrel{\lower1pt\hbox{$\scriptscriptstyle\smile$}}\over{\mathfrak{W}}}}_{{\mathfrak{L}}_{\tau }}}},\overline{\overline{{\mathord{\buildrel{\lower1pt\hbox{$\scriptscriptstyle\smile$}}\over{\mathfrak{W}}}}_{{\mathfrak{L}}_{k}}}}\right)=1-Dis\left(\overline{\overline{{\mathord{\buildrel{\lower1pt\hbox{$\scriptscriptstyle\smile$}}\over{\mathfrak{W}}}}_{{\mathfrak{L}}_{\tau }}}},\overline{\overline{{\mathord{\buildrel{\lower1pt\hbox{$\scriptscriptstyle\smile$}}\over{\mathfrak{W}}}}_{{\mathfrak{L}}_{k}}}}\right)$$ and $$Dis\left(\overline{\overline{{\mathord{\buildrel{\lower1pt\hbox{$\scriptscriptstyle\smile$}}\over{\mathfrak{W}}}}_{{\mathfrak{L}}_{\tau }}}},\overline{\overline{{\mathord{\buildrel{\lower1pt\hbox{$\scriptscriptstyle\smile$}}\over{\mathfrak{W}}}}_{{\mathfrak{L}}_{k}}}}\right)=\frac{1}{4}\left(\left|{\overline{\overline{{\zeta }_{\overline{\overline{{\mathord{\buildrel{\lower1pt\hbox{$\scriptscriptstyle\smile$}}\over{\mathfrak{W}}}}_{{\mathfrak{L}}_{\tau }}}}}}}}^{l}-{\overline{\overline{{\zeta }_{\overline{\overline{{\mathord{\buildrel{\lower1pt\hbox{$\scriptscriptstyle\smile$}}\over{\mathfrak{W}}}}_{{\mathfrak{L}}_{k}}}}}}}}^{l}\right|+\left|{\overline{\overline{{\eta }_{\overline{\overline{{\mathord{\buildrel{\lower1pt\hbox{$\scriptscriptstyle\smile$}}\over{\mathfrak{W}}}}_{{\mathfrak{L}}_{\tau }}}}}}}}^{l}-{\overline{\overline{{\eta }_{\overline{\overline{{\mathord{\buildrel{\lower1pt\hbox{$\scriptscriptstyle\smile$}}\over{\mathfrak{W}}}}_{{\mathfrak{L}}_{k}}}}}}}}^{l}\right|+\left|{\overline{\overline{{\zeta }_{\overline{\overline{{\mathord{\buildrel{\lower1pt\hbox{$\scriptscriptstyle\smile$}}\over{\mathfrak{W}}}}_{{\mathfrak{L}}_{\tau }}}}}}}}^{u}-{\overline{\overline{{\zeta }_{\overline{\overline{{\mathord{\buildrel{\lower1pt\hbox{$\scriptscriptstyle\smile$}}\over{\mathfrak{W}}}}_{{\mathfrak{L}}_{k}}}}}}}}^{u}\right|+\left|{\overline{\overline{{\eta }_{\overline{\overline{{\mathord{\buildrel{\lower1pt\hbox{$\scriptscriptstyle\smile$}}\over{\mathfrak{W}}}}_{{\mathfrak{L}}_{\tau }}}}}}}}^{u}-{\overline{\overline{{\eta }_{\overline{\overline{{\mathord{\buildrel{\lower1pt\hbox{$\scriptscriptstyle\smile$}}\over{\mathfrak{W}}}}_{{\mathfrak{L}}_{k}}}}}}}}^{u}\right|\right),$$ which is the distance information between $$\overline{\overline{{\mathord{\buildrel{\lower1pt\hbox{$\scriptscriptstyle\smile$}}\over{\mathfrak{W}}}}_{{\mathfrak{L}}_{\tau }}}} and \overline{\overline{{\mathord{\buildrel{\lower1pt\hbox{$\scriptscriptstyle\smile$}}\over{\mathfrak{W}}}}_{{\mathfrak{L}}_{k}}}}.$$

### Theorem 2

For a group of IVA-IFNs $$\overline{\overline{{\mathord{\buildrel{\lower1pt\hbox{$\scriptscriptstyle\smile$}}\over{\mathfrak{W}}}}_{{\mathfrak{L}}_{\tau }}}}=\left(\left[{\overline{\overline{{\zeta }_{\overline{\overline{{\mathord{\buildrel{\lower1pt\hbox{$\scriptscriptstyle\smile$}}\over{\mathfrak{W}}}}_{{\mathfrak{L}}_{\tau }}}}}}}}^{l},{\overline{\overline{{\zeta }_{\overline{\overline{{\mathord{\buildrel{\lower1pt\hbox{$\scriptscriptstyle\smile$}}\over{\mathfrak{W}}}}_{{\mathfrak{L}}_{\tau }}}}}}}}^{u}\right],\left[{\overline{\overline{{\eta }_{\overline{\overline{{\mathord{\buildrel{\lower1pt\hbox{$\scriptscriptstyle\smile$}}\over{\mathfrak{W}}}}_{{\mathfrak{L}}_{\tau }}}}}}}}^{l},{\overline{\overline{{\eta }_{\overline{\overline{{\mathord{\buildrel{\lower1pt\hbox{$\scriptscriptstyle\smile$}}\over{\mathfrak{W}}}}_{{\mathfrak{L}}_{\tau }}}}}}}}^{u}\right]\right),\tau =\mathrm{1,2},\dots ,{\rm Z},$$ the aggregation result from Eq. (15) is still an IVA-IFN, such as

16 $$IVA-IFAAPOA\left(\overline{\overline{{\mathord{\buildrel{\lower1pt\hbox{$\scriptscriptstyle\smile$}}\over{\mathfrak{W}}}}_{{\mathfrak{L}}_{1}}}},\overline{\overline{{\mathord{\buildrel{\lower1pt\hbox{$\scriptscriptstyle\smile$}}\over{\mathfrak{W}}}}_{{\mathfrak{L}}_{2}}}},\dots ,\overline{\overline{{\mathord{\buildrel{\lower1pt\hbox{$\scriptscriptstyle\smile$}}\over{\mathfrak{W}}}}_{{\mathfrak{L}}_{\rm Z}}}}\right)=\left(\begin{array}{c}\left[1-{e}^{-{\left(\sum_{\tau =1}^{\rm Z}\overline{\overline{{\Xi }_{\tau }}}{\left(-\mathrm{log}\left(1-{\overline{\overline{{\zeta }_{\overline{\overline{{\mathord{\buildrel{\lower1pt\hbox{$\scriptscriptstyle\smile$}}\over{\mathfrak{W}}}}_{{\mathfrak{L}}_{0\left(\tau \right)}}}}}}}}^{l}\right)\right)}^{\Lambda }\right)}^{\frac{1}{\Lambda }}},1-{e}^{-{\left(\sum_{\tau =1}^{\rm Z}\overline{\overline{{\Xi }_{\tau }}}{\left(-\mathrm{log}\left(1-{\overline{\overline{{\zeta }_{\overline{\overline{{\mathord{\buildrel{\lower1pt\hbox{$\scriptscriptstyle\smile$}}\over{\mathfrak{W}}}}_{{\mathfrak{L}}_{0\left(\tau \right)}}}}}}}}^{u}\right)\right)}^{\Lambda }\right)}^{\frac{1}{\Lambda }}}\right],\\ \left[{e}^{-{\left(\sum_{\tau =1}^{\rm Z}\overline{\overline{{\Xi }_{\tau }}}{\left(-\mathrm{log}\left({\overline{\overline{{\eta }_{\overline{\overline{{\mathord{\buildrel{\lower1pt\hbox{$\scriptscriptstyle\smile$}}\over{\mathfrak{W}}}}_{{\mathfrak{L}}_{0\left(\tau \right)}}}}}}}}^{l}\right)\right)}^{\Lambda }\right)}^{\frac{1}{\Lambda }}},{e}^{-{\left(\sum_{\tau =1}^{\rm Z}\overline{\overline{{\Xi }_{\tau }}}{\left(-\mathrm{log}\left({\overline{\overline{{\eta }_{\overline{\overline{{\mathord{\buildrel{\lower1pt\hbox{$\scriptscriptstyle\smile$}}\over{\mathfrak{W}}}}_{{\mathfrak{L}}_{0\left(\tau \right)}}}}}}}}^{u}\right)\right)}^{\Lambda }\right)}^{\frac{1}{\Lambda }}}\right]\end{array}\right)$$

### Property 4 

(Idempotency) For a group of IVA-IFNs $$\overline{\overline{{\mathord{\buildrel{\lower1pt\hbox{$\scriptscriptstyle\smile$}}\over{\mathfrak{W}}}}_{{\mathfrak{L}}_{\tau }}}}=\left(\left[{\overline{\overline{{\zeta }_{\overline{\overline{{\mathord{\buildrel{\lower1pt\hbox{$\scriptscriptstyle\smile$}}\over{\mathfrak{W}}}}_{{\mathfrak{L}}_{\tau }}}}}}}}^{l},{\overline{\overline{{\zeta }_{\overline{\overline{{\mathord{\buildrel{\lower1pt\hbox{$\scriptscriptstyle\smile$}}\over{\mathfrak{W}}}}_{{\mathfrak{L}}_{\tau }}}}}}}}^{u}\right],\left[{\overline{\overline{{\eta }_{\overline{\overline{{\mathord{\buildrel{\lower1pt\hbox{$\scriptscriptstyle\smile$}}\over{\mathfrak{W}}}}_{{\mathfrak{L}}_{\tau }}}}}}}}^{l},{\overline{\overline{{\eta }_{\overline{\overline{{\mathord{\buildrel{\lower1pt\hbox{$\scriptscriptstyle\smile$}}\over{\mathfrak{W}}}}_{{\mathfrak{L}}_{\tau }}}}}}}}^{u}\right]\right),\tau =\mathrm{1,2},\dots ,{\rm Z},$$ If $$\overline{\overline{{\mathord{\buildrel{\lower1pt\hbox{$\scriptscriptstyle\smile$}}\over{\mathfrak{W}}}}_{{\mathfrak{L}}_{\tau }}}}=\overline{\overline{{\mathord{\buildrel{\lower1pt\hbox{$\scriptscriptstyle\smile$}}\over{\mathfrak{W}}}}_{\mathfrak{L}}}}=\left(\left[{\overline{\overline{{\zeta }_{\overline{\overline{{\mathord{\buildrel{\lower1pt\hbox{$\scriptscriptstyle\smile$}}\over{\mathfrak{W}}}}_{\mathfrak{L}}}}}}}}^{l},{\overline{\overline{{\zeta }_{\overline{\overline{{\mathord{\buildrel{\lower1pt\hbox{$\scriptscriptstyle\smile$}}\over{\mathfrak{W}}}}_{\mathfrak{L}}}}}}}}^{u}\right],\left[{\overline{\overline{{\eta }_{\overline{\overline{{\mathord{\buildrel{\lower1pt\hbox{$\scriptscriptstyle\smile$}}\over{\mathfrak{W}}}}_{\mathfrak{L}}}}}}}}^{l},{\overline{\overline{{\eta }_{\overline{\overline{{\mathord{\buildrel{\lower1pt\hbox{$\scriptscriptstyle\smile$}}\over{\mathfrak{W}}}}_{\mathfrak{L}}}}}}}}^{u}\right]\right),$$ then

17 $$IVA-IFAAPOA\left(\overline{\overline{{\mathord{\buildrel{\lower1pt\hbox{$\scriptscriptstyle\smile$}}\over{\mathfrak{W}}}}_{{\mathfrak{L}}_{1}}}},\overline{\overline{{\mathord{\buildrel{\lower1pt\hbox{$\scriptscriptstyle\smile$}}\over{\mathfrak{W}}}}_{{\mathfrak{L}}_{2}}}},\dots ,\overline{\overline{{\mathord{\buildrel{\lower1pt\hbox{$\scriptscriptstyle\smile$}}\over{\mathfrak{W}}}}_{{\mathfrak{L}}_{\rm Z}}}}\right)=\overline{\overline{{\mathord{\buildrel{\lower1pt\hbox{$\scriptscriptstyle\smile$}}\over{\mathfrak{W}}}}_{\mathfrak{L}}}}$$

Further, the monotonicity is not kept. Further, we give the boundedness.

### Property 5 

(Boundedness) For a group of IVA-IFNs $$\overline{\overline{{\mathord{\buildrel{\lower1pt\hbox{$\scriptscriptstyle\smile$}}\over{\mathfrak{W}}}}_{{\mathfrak{L}}_{\tau }}}}=\left(\left[{\overline{\overline{{\zeta }_{\overline{\overline{{\mathord{\buildrel{\lower1pt\hbox{$\scriptscriptstyle\smile$}}\over{\mathfrak{W}}}}_{{\mathfrak{L}}_{\tau }}}}}}}}^{l},{\overline{\overline{{\zeta }_{\overline{\overline{{\mathord{\buildrel{\lower1pt\hbox{$\scriptscriptstyle\smile$}}\over{\mathfrak{W}}}}_{{\mathfrak{L}}_{\tau }}}}}}}}^{u}\right],\left[{\overline{\overline{{\eta }_{\overline{\overline{{\mathord{\buildrel{\lower1pt\hbox{$\scriptscriptstyle\smile$}}\over{\mathfrak{W}}}}_{{\mathfrak{L}}_{\tau }}}}}}}}^{l},{\overline{\overline{{\eta }_{\overline{\overline{{\mathord{\buildrel{\lower1pt\hbox{$\scriptscriptstyle\smile$}}\over{\mathfrak{W}}}}_{{\mathfrak{L}}_{\tau }}}}}}}}^{u}\right]\right),\tau =\mathrm{1,2},\dots ,{\rm Z},$$ If $${\overline{\overline{{\mathord{\buildrel{\lower1pt\hbox{$\scriptscriptstyle\smile$}}\over{\mathfrak{W}}}}_{{\mathfrak{L}}_{\tau }}}}}^{-}=\left(\left[\underset{\tau }{\mathrm{min}}{\overline{\overline{{\zeta }_{\overline{\overline{{\mathord{\buildrel{\lower1pt\hbox{$\scriptscriptstyle\smile$}}\over{\mathfrak{W}}}}_{{\mathfrak{L}}_{\tau }}}}}}}}^{l},\underset{\tau }{\mathrm{min}}{\overline{\overline{{\zeta }_{\overline{\overline{{\mathord{\buildrel{\lower1pt\hbox{$\scriptscriptstyle\smile$}}\over{\mathfrak{W}}}}_{{\mathfrak{L}}_{\tau }}}}}}}}^{u}\right],\left[\underset{\tau }{\mathrm{max}}{\overline{\overline{{\eta }_{\overline{\overline{{\mathord{\buildrel{\lower1pt\hbox{$\scriptscriptstyle\smile$}}\over{\mathfrak{W}}}}_{{\mathfrak{L}}_{\tau }}}}}}}}^{l},\underset{\tau }{\mathrm{max}}{\overline{\overline{{\eta }_{\overline{\overline{{\mathord{\buildrel{\lower1pt\hbox{$\scriptscriptstyle\smile$}}\over{\mathfrak{W}}}}_{{\mathfrak{L}}_{\tau }}}}}}}}^{u}\right]\right)$$ and $${\overline{\overline{{\mathord{\buildrel{\lower1pt\hbox{$\scriptscriptstyle\smile$}}\over{\mathfrak{W}}}}_{{\mathfrak{L}}_{\tau }}}}}^{+}=\left(\left[\underset{\tau }{\mathrm{max}}{\overline{\overline{{\zeta }_{\overline{\overline{{\mathord{\buildrel{\lower1pt\hbox{$\scriptscriptstyle\smile$}}\over{\mathfrak{W}}}}_{{\mathfrak{L}}_{\tau }}}}}}}}^{l},\underset{\tau }{\mathrm{max}}{\overline{\overline{{\zeta }_{\overline{\overline{{\mathord{\buildrel{\lower1pt\hbox{$\scriptscriptstyle\smile$}}\over{\mathfrak{W}}}}_{{\mathfrak{L}}_{\tau }}}}}}}}^{u}\right],\left[\underset{\tau }{\mathrm{min}}{\overline{\overline{{\eta }_{\overline{\overline{{\mathord{\buildrel{\lower1pt\hbox{$\scriptscriptstyle\smile$}}\over{\mathfrak{W}}}}_{{\mathfrak{L}}_{\tau }}}}}}}}^{l},\underset{\tau }{\mathrm{min}}{\overline{\overline{{\eta }_{\overline{\overline{{\mathord{\buildrel{\lower1pt\hbox{$\scriptscriptstyle\smile$}}\over{\mathfrak{W}}}}_{{\mathfrak{L}}_{\tau }}}}}}}}^{u}\right]\right),$$ then

$${\overline{\overline{{\mathord{\buildrel{\lower1pt\hbox{$\scriptscriptstyle\smile$}}\over{\mathfrak{W}}}}_{{\mathfrak{L}}_{\tau }}}}}^{-}\le IVA-IFAAPOA\left(\overline{\overline{{\mathord{\buildrel{\lower1pt\hbox{$\scriptscriptstyle\smile$}}\over{\mathfrak{W}}}}_{{\mathfrak{L}}_{1}}}},\overline{\overline{{\mathord{\buildrel{\lower1pt\hbox{$\scriptscriptstyle\smile$}}\over{\mathfrak{W}}}}_{{\mathfrak{L}}_{2}}}},\dots ,\overline{\overline{{\mathord{\buildrel{\lower1pt\hbox{$\scriptscriptstyle\smile$}}\over{\mathfrak{W}}}}_{{\mathfrak{L}}_{\rm Z}}}}\right)\le {\overline{\overline{{\mathord{\buildrel{\lower1pt\hbox{$\scriptscriptstyle\smile$}}\over{\mathfrak{W}}}}_{{\mathfrak{L}}_{\tau }}}}}^{+}$$

### Definition 7

For a group of IVA-IFNs $$\overline{\overline{{\mathord{\buildrel{\lower1pt\hbox{$\scriptscriptstyle\smile$}}\over{\mathfrak{W}}}}_{{\mathfrak{L}}_{\tau }}}}=\left(\left[{\overline{\overline{{\zeta }_{\overline{\overline{{\mathord{\buildrel{\lower1pt\hbox{$\scriptscriptstyle\smile$}}\over{\mathfrak{W}}}}_{{\mathfrak{L}}_{\tau }}}}}}}}^{l},{\overline{\overline{{\zeta }_{\overline{\overline{{\mathord{\buildrel{\lower1pt\hbox{$\scriptscriptstyle\smile$}}\over{\mathfrak{W}}}}_{{\mathfrak{L}}_{\tau }}}}}}}}^{u}\right],\left[{\overline{\overline{{\eta }_{\overline{\overline{{\mathord{\buildrel{\lower1pt\hbox{$\scriptscriptstyle\smile$}}\over{\mathfrak{W}}}}_{{\mathfrak{L}}_{\tau }}}}}}}}^{l},{\overline{\overline{{\eta }_{\overline{\overline{{\mathord{\buildrel{\lower1pt\hbox{$\scriptscriptstyle\smile$}}\over{\mathfrak{W}}}}_{{\mathfrak{L}}_{\tau }}}}}}}}^{u}\right]\right),\tau =\mathrm{1,2},\dots ,{\rm Z},$$ then an IVA-IFAAPG operator is described as

18 $$IVA-IFAAPG\left(\overline{\overline{{\mathord{\buildrel{\lower1pt\hbox{$\scriptscriptstyle\smile$}}\over{\mathfrak{W}}}}_{{\mathfrak{L}}_{1}}}},\overline{\overline{{\mathord{\buildrel{\lower1pt\hbox{$\scriptscriptstyle\smile$}}\over{\mathfrak{W}}}}_{{\mathfrak{L}}_{2}}}},\dots ,\overline{\overline{{\mathord{\buildrel{\lower1pt\hbox{$\scriptscriptstyle\smile$}}\over{\mathfrak{W}}}}_{{\mathfrak{L}}_{\rm Z}}}}\right)={\overline{\overline{{\mathord{\buildrel{\lower1pt\hbox{$\scriptscriptstyle\smile$}}\over{\mathfrak{W}}}}_{{\mathfrak{L}}_{1}}}}}^{\overline{\overline{{\Xi }_{1}}}}\otimes {\overline{\overline{{\mathord{\buildrel{\lower1pt\hbox{$\scriptscriptstyle\smile$}}\over{\mathfrak{W}}}}_{{\mathfrak{L}}_{2}}}}}^{\overline{\overline{{\Xi }_{2}}}}\otimes \dots \otimes {\overline{\overline{{\mathord{\buildrel{\lower1pt\hbox{$\scriptscriptstyle\smile$}}\over{\mathfrak{W}}}}_{{\mathfrak{L}}_{\rm Z}}}}}^{\overline{\overline{{\Xi }_{\rm Z}}}}={\otimes }_{\tau =1}^{\rm Z}\left({\overline{\overline{{\mathord{\buildrel{\lower1pt\hbox{$\scriptscriptstyle\smile$}}\over{\mathfrak{W}}}}_{{\mathfrak{L}}_{\tau }}}}}^{\overline{\overline{{\Xi }_{\tau }}}}\right)$$

Noticed that $$\overline{\overline{{\Xi }_{\tau }}}=\frac{\left(1+\overline{\overline{\mathfrak{I}}}\left(\overline{\overline{{\mathord{\buildrel{\lower1pt\hbox{$\scriptscriptstyle\smile$}}\over{\mathfrak{W}}}}_{{\mathfrak{L}}_{\tau }}}}\right)\right)}{\sum_{\tau =1}^{\rm Z}\left(1+\overline{\overline{\mathfrak{I}}}\left(\overline{\overline{{\mathord{\buildrel{\lower1pt\hbox{$\scriptscriptstyle\smile$}}\over{\mathfrak{W}}}}_{{\mathfrak{L}}_{\tau }}}}\right)\right)}$$. Further,$$\overline{\overline{\mathfrak{I}}}\left(\overline{\overline{{\mathord{\buildrel{\lower1pt\hbox{$\scriptscriptstyle\smile$}}\over{\mathfrak{W}}}}_{{\mathfrak{L}}_{\tau }}}}\right)=\sum_{\begin{array}{c}s=1,\\ s\ne \tau \end{array}}^{\rm Z}Sup\left(\overline{\overline{{\mathord{\buildrel{\lower1pt\hbox{$\scriptscriptstyle\smile$}}\over{\mathfrak{W}}}}_{{\mathfrak{L}}_{\tau }}}},\overline{\overline{{\mathord{\buildrel{\lower1pt\hbox{$\scriptscriptstyle\smile$}}\over{\mathfrak{W}}}}_{{\mathfrak{L}}_{k}}}}\right),$$ which expresses the relationship between $$\overline{\overline{{\mathord{\buildrel{\lower1pt\hbox{$\scriptscriptstyle\smile$}}\over{\mathfrak{W}}}}_{{\mathfrak{L}}_{\tau }}}}$$ and $$\overline{\overline{{\mathord{\buildrel{\lower1pt\hbox{$\scriptscriptstyle\smile$}}\over{\mathfrak{W}}}}_{{\mathfrak{L}}_{k}}}},$$ where $$Sup\left(\overline{\overline{{\mathord{\buildrel{\lower1pt\hbox{$\scriptscriptstyle\smile$}}\over{\mathfrak{W}}}}_{{\mathfrak{L}}_{\tau }}}},\overline{\overline{{\mathord{\buildrel{\lower1pt\hbox{$\scriptscriptstyle\smile$}}\over{\mathfrak{W}}}}_{{\mathfrak{L}}_{k}}}}\right)=1-Dis\left(\overline{\overline{{\mathord{\buildrel{\lower1pt\hbox{$\scriptscriptstyle\smile$}}\over{\mathfrak{W}}}}_{{\mathfrak{L}}_{\tau }}}},\overline{\overline{{\mathord{\buildrel{\lower1pt\hbox{$\scriptscriptstyle\smile$}}\over{\mathfrak{W}}}}_{{\mathfrak{L}}_{k}}}}\right)$$ and$$Dis\left(\overline{\overline{{\mathord{\buildrel{\lower1pt\hbox{$\scriptscriptstyle\smile$}}\over{\mathfrak{W}}}}_{{\mathfrak{L}}_{\tau }}}},\overline{\overline{{\mathord{\buildrel{\lower1pt\hbox{$\scriptscriptstyle\smile$}}\over{\mathfrak{W}}}}_{{\mathfrak{L}}_{k}}}}\right)=\frac{1}{4}\left(\left|{\overline{\overline{{\zeta }_{\overline{\overline{{\mathord{\buildrel{\lower1pt\hbox{$\scriptscriptstyle\smile$}}\over{\mathfrak{W}}}}_{{\mathfrak{L}}_{\tau }}}}}}}}^{l}-{\overline{\overline{{\zeta }_{\overline{\overline{{\mathord{\buildrel{\lower1pt\hbox{$\scriptscriptstyle\smile$}}\over{\mathfrak{W}}}}_{{\mathfrak{L}}_{k}}}}}}}}^{l}\right|+\left|{\overline{\overline{{\eta }_{\overline{\overline{{\mathord{\buildrel{\lower1pt\hbox{$\scriptscriptstyle\smile$}}\over{\mathfrak{W}}}}_{{\mathfrak{L}}_{\tau }}}}}}}}^{l}-{\overline{\overline{{\eta }_{\overline{\overline{{\mathord{\buildrel{\lower1pt\hbox{$\scriptscriptstyle\smile$}}\over{\mathfrak{W}}}}_{{\mathfrak{L}}_{k}}}}}}}}^{l}\right|+\left|{\overline{\overline{{\zeta }_{\overline{\overline{{\mathord{\buildrel{\lower1pt\hbox{$\scriptscriptstyle\smile$}}\over{\mathfrak{W}}}}_{{\mathfrak{L}}_{\tau }}}}}}}}^{u}-{\overline{\overline{{\zeta }_{\overline{\overline{{\mathord{\buildrel{\lower1pt\hbox{$\scriptscriptstyle\smile$}}\over{\mathfrak{W}}}}_{{\mathfrak{L}}_{k}}}}}}}}^{u}\right|+\left|{\overline{\overline{{\eta }_{\overline{\overline{{\mathord{\buildrel{\lower1pt\hbox{$\scriptscriptstyle\smile$}}\over{\mathfrak{W}}}}_{{\mathfrak{L}}_{\tau }}}}}}}}^{u}-{\overline{\overline{{\eta }_{\overline{\overline{{\mathord{\buildrel{\lower1pt\hbox{$\scriptscriptstyle\smile$}}\over{\mathfrak{W}}}}_{{\mathfrak{L}}_{k}}}}}}}}^{u}\right|\right).$$

### Theorem 3

For a group of IVA-IFNs $$\overline{\overline{{\mathord{\buildrel{\lower1pt\hbox{$\scriptscriptstyle\smile$}}\over{\mathfrak{W}}}}_{{\mathfrak{L}}_{\tau }}}}=\left(\left[{\overline{\overline{{\zeta }_{\overline{\overline{{\mathord{\buildrel{\lower1pt\hbox{$\scriptscriptstyle\smile$}}\over{\mathfrak{W}}}}_{{\mathfrak{L}}_{\tau }}}}}}}}^{l},{\overline{\overline{{\zeta }_{\overline{\overline{{\mathord{\buildrel{\lower1pt\hbox{$\scriptscriptstyle\smile$}}\over{\mathfrak{W}}}}_{{\mathfrak{L}}_{\tau }}}}}}}}^{u}\right],\left[{\overline{\overline{{\eta }_{\overline{\overline{{\mathord{\buildrel{\lower1pt\hbox{$\scriptscriptstyle\smile$}}\over{\mathfrak{W}}}}_{{\mathfrak{L}}_{\tau }}}}}}}}^{l},{\overline{\overline{{\eta }_{\overline{\overline{{\mathord{\buildrel{\lower1pt\hbox{$\scriptscriptstyle\smile$}}\over{\mathfrak{W}}}}_{{\mathfrak{L}}_{\tau }}}}}}}}^{u}\right]\right),\tau =\mathrm{1,2},\dots ,{\rm Z},$$ the aggregation result from Eq. (18) is still an IVA-IFN, such as

19 $$IVA-IFAAPG\left(\overline{\overline{{\mathord{\buildrel{\lower1pt\hbox{$\scriptscriptstyle\smile$}}\over{\mathfrak{W}}}}_{{\mathfrak{L}}_{1}}}},\overline{\overline{{\mathord{\buildrel{\lower1pt\hbox{$\scriptscriptstyle\smile$}}\over{\mathfrak{W}}}}_{{\mathfrak{L}}_{2}}}},\dots ,\overline{\overline{{\mathord{\buildrel{\lower1pt\hbox{$\scriptscriptstyle\smile$}}\over{\mathfrak{W}}}}_{{\mathfrak{L}}_{\rm Z}}}}\right)=\left(\begin{array}{c}\left[{e}^{-{\left(\sum_{\tau =1}^{\rm Z}\overline{\overline{{\Xi }_{\tau }}}{\left(-\mathrm{log}\left({\overline{\overline{{\zeta }_{\overline{\overline{{\mathord{\buildrel{\lower1pt\hbox{$\scriptscriptstyle\smile$}}\over{\mathfrak{W}}}}_{{\mathfrak{L}}_{\tau }}}}}}}}^{l}\right)\right)}^{\Lambda }\right)}^{\frac{1}{\Lambda }}},{e}^{-{\left(\sum_{\tau =1}^{\rm Z}\overline{\overline{{\Xi }_{\tau }}}{\left(-\mathrm{log}\left({\overline{\overline{{\zeta }_{\overline{\overline{{\mathord{\buildrel{\lower1pt\hbox{$\scriptscriptstyle\smile$}}\over{\mathfrak{W}}}}_{{\mathfrak{L}}_{\tau }}}}}}}}^{u}\right)\right)}^{\Lambda }\right)}^{\frac{1}{\Lambda }}}\right],\\ \left[1-{e}^{-{\left(\sum_{\tau =1}^{\rm Z}\overline{\overline{{\Xi }_{\tau }}}{\left(-\mathrm{log}\left(1-{\overline{\overline{{\eta }_{\overline{\overline{{\mathord{\buildrel{\lower1pt\hbox{$\scriptscriptstyle\smile$}}\over{\mathfrak{W}}}}_{{\mathfrak{L}}_{\tau }}}}}}}}^{l}\right)\right)}^{\Lambda }\right)}^{\frac{1}{\Lambda }}},1-{e}^{-{\left(\sum_{\tau =1}^{\rm Z}\overline{\overline{{\Xi }_{\tau }}}{\left(-\mathrm{log}\left(1-{\overline{\overline{{\eta }_{\overline{\overline{{\mathord{\buildrel{\lower1pt\hbox{$\scriptscriptstyle\smile$}}\over{\mathfrak{W}}}}_{{\mathfrak{L}}_{\tau }}}}}}}}^{u}\right)\right)}^{\Lambda }\right)}^{\frac{1}{\Lambda }}}\right]\end{array}\right)$$

### Property 6 

(Idempotency) For a group of IVA-IFNs $$\overline{\overline{{\mathord{\buildrel{\lower1pt\hbox{$\scriptscriptstyle\smile$}}\over{\mathfrak{W}}}}_{{\mathfrak{L}}_{\tau }}}}=\left(\left[{\overline{\overline{{\zeta }_{\overline{\overline{{\mathord{\buildrel{\lower1pt\hbox{$\scriptscriptstyle\smile$}}\over{\mathfrak{W}}}}_{{\mathfrak{L}}_{\tau }}}}}}}}^{l},{\overline{\overline{{\zeta }_{\overline{\overline{{\mathord{\buildrel{\lower1pt\hbox{$\scriptscriptstyle\smile$}}\over{\mathfrak{W}}}}_{{\mathfrak{L}}_{\tau }}}}}}}}^{u}\right],\left[{\overline{\overline{{\eta }_{\overline{\overline{{\mathord{\buildrel{\lower1pt\hbox{$\scriptscriptstyle\smile$}}\over{\mathfrak{W}}}}_{{\mathfrak{L}}_{\tau }}}}}}}}^{l},{\overline{\overline{{\eta }_{\overline{\overline{{\mathord{\buildrel{\lower1pt\hbox{$\scriptscriptstyle\smile$}}\over{\mathfrak{W}}}}_{{\mathfrak{L}}_{\tau }}}}}}}}^{u}\right]\right),\tau =\mathrm{1,2},\dots ,{\rm Z},$$ If $$\overline{\overline{{\mathord{\buildrel{\lower1pt\hbox{$\scriptscriptstyle\smile$}}\over{\mathfrak{W}}}}_{{\mathfrak{L}}_{\tau }}}}=\overline{\overline{{\mathord{\buildrel{\lower1pt\hbox{$\scriptscriptstyle\smile$}}\over{\mathfrak{W}}}}_{\mathfrak{L}}}}=\left(\left[{\overline{\overline{{\zeta }_{\overline{\overline{{\mathord{\buildrel{\lower1pt\hbox{$\scriptscriptstyle\smile$}}\over{\mathfrak{W}}}}_{\mathfrak{L}}}}}}}}^{l},{\overline{\overline{{\zeta }_{\overline{\overline{{\mathord{\buildrel{\lower1pt\hbox{$\scriptscriptstyle\smile$}}\over{\mathfrak{W}}}}_{\mathfrak{L}}}}}}}}^{u}\right],\left[{\overline{\overline{{\eta }_{\overline{\overline{{\mathord{\buildrel{\lower1pt\hbox{$\scriptscriptstyle\smile$}}\over{\mathfrak{W}}}}_{\mathfrak{L}}}}}}}}^{l},{\overline{\overline{{\eta }_{\overline{\overline{{\mathord{\buildrel{\lower1pt\hbox{$\scriptscriptstyle\smile$}}\over{\mathfrak{W}}}}_{\mathfrak{L}}}}}}}}^{u}\right]\right),$$ then

20 $$IVA-IFAAPG\left(\overline{\overline{{\mathord{\buildrel{\lower1pt\hbox{$\scriptscriptstyle\smile$}}\over{\mathfrak{W}}}}_{{\mathfrak{L}}_{1}}}},\overline{\overline{{\mathord{\buildrel{\lower1pt\hbox{$\scriptscriptstyle\smile$}}\over{\mathfrak{W}}}}_{{\mathfrak{L}}_{2}}}},\dots ,\overline{\overline{{\mathord{\buildrel{\lower1pt\hbox{$\scriptscriptstyle\smile$}}\over{\mathfrak{W}}}}_{{\mathfrak{L}}_{\rm Z}}}}\right)=\overline{\overline{{\mathord{\buildrel{\lower1pt\hbox{$\scriptscriptstyle\smile$}}\over{\mathfrak{W}}}}_{\mathfrak{L}}}}$$

Further, the monotonicity is not kept.

### Property 7 

 (Boundedness) For a group of IVA-IFNs $$\overline{\overline{{\mathord{\buildrel{\lower1pt\hbox{$\scriptscriptstyle\smile$}}\over{\mathfrak{W}}}}_{{\mathfrak{L}}_{\tau }}}}=\left(\left[{\overline{\overline{{\zeta }_{\overline{\overline{{\mathord{\buildrel{\lower1pt\hbox{$\scriptscriptstyle\smile$}}\over{\mathfrak{W}}}}_{{\mathfrak{L}}_{\tau }}}}}}}}^{l},{\overline{\overline{{\zeta }_{\overline{\overline{{\mathord{\buildrel{\lower1pt\hbox{$\scriptscriptstyle\smile$}}\over{\mathfrak{W}}}}_{{\mathfrak{L}}_{\tau }}}}}}}}^{u}\right],\left[{\overline{\overline{{\eta }_{\overline{\overline{{\mathord{\buildrel{\lower1pt\hbox{$\scriptscriptstyle\smile$}}\over{\mathfrak{W}}}}_{{\mathfrak{L}}_{\tau }}}}}}}}^{l},{\overline{\overline{{\eta }_{\overline{\overline{{\mathord{\buildrel{\lower1pt\hbox{$\scriptscriptstyle\smile$}}\over{\mathfrak{W}}}}_{{\mathfrak{L}}_{\tau }}}}}}}}^{u}\right]\right),\tau =\mathrm{1,2},\dots ,{\rm Z},$$ If $${\overline{\overline{{\mathord{\buildrel{\lower1pt\hbox{$\scriptscriptstyle\smile$}}\over{\mathfrak{W}}}}_{{\mathfrak{L}}_{\tau }}}}}^{-}=\left(\left[\underset{\tau }{\mathrm{min}}{\overline{\overline{{\zeta }_{\overline{\overline{{\mathord{\buildrel{\lower1pt\hbox{$\scriptscriptstyle\smile$}}\over{\mathfrak{W}}}}_{{\mathfrak{L}}_{\tau }}}}}}}}^{l},\underset{\tau }{\mathrm{min}}{\overline{\overline{{\zeta }_{\overline{\overline{{\mathord{\buildrel{\lower1pt\hbox{$\scriptscriptstyle\smile$}}\over{\mathfrak{W}}}}_{{\mathfrak{L}}_{\tau }}}}}}}}^{u}\right],\left[\underset{\tau }{\mathrm{max}}{\overline{\overline{{\eta }_{\overline{\overline{{\mathord{\buildrel{\lower1pt\hbox{$\scriptscriptstyle\smile$}}\over{\mathfrak{W}}}}_{{\mathfrak{L}}_{\tau }}}}}}}}^{l},\underset{\tau }{\mathrm{max}}{\overline{\overline{{\eta }_{\overline{\overline{{\mathord{\buildrel{\lower1pt\hbox{$\scriptscriptstyle\smile$}}\over{\mathfrak{W}}}}_{{\mathfrak{L}}_{\tau }}}}}}}}^{u}\right]\right)$$ and $${\overline{\overline{{\mathord{\buildrel{\lower1pt\hbox{$\scriptscriptstyle\smile$}}\over{\mathfrak{W}}}}_{{\mathfrak{L}}_{\tau }}}}}^{+}=\left(\left[\underset{\tau }{\mathrm{max}}{\overline{\overline{{\zeta }_{\overline{\overline{{\mathord{\buildrel{\lower1pt\hbox{$\scriptscriptstyle\smile$}}\over{\mathfrak{W}}}}_{{\mathfrak{L}}_{\tau }}}}}}}}^{l},\underset{\tau }{\mathrm{max}}{\overline{\overline{{\zeta }_{\overline{\overline{{\mathord{\buildrel{\lower1pt\hbox{$\scriptscriptstyle\smile$}}\over{\mathfrak{W}}}}_{{\mathfrak{L}}_{\tau }}}}}}}}^{u}\right],\left[\underset{\tau }{\mathrm{min}}{\overline{\overline{{\eta }_{\overline{\overline{{\mathord{\buildrel{\lower1pt\hbox{$\scriptscriptstyle\smile$}}\over{\mathfrak{W}}}}_{{\mathfrak{L}}_{\tau }}}}}}}}^{l},\underset{\tau }{\mathrm{min}}{\overline{\overline{{\eta }_{\overline{\overline{{\mathord{\buildrel{\lower1pt\hbox{$\scriptscriptstyle\smile$}}\over{\mathfrak{W}}}}_{{\mathfrak{L}}_{\tau }}}}}}}}^{u}\right]\right),$$ then

$${\overline{\overline{{\mathord{\buildrel{\lower1pt\hbox{$\scriptscriptstyle\smile$}}\over{\mathfrak{W}}}}_{{\mathfrak{L}}_{\tau }}}}}^{-}\le IVA-IFAAPG\left(\overline{\overline{{\mathord{\buildrel{\lower1pt\hbox{$\scriptscriptstyle\smile$}}\over{\mathfrak{W}}}}_{{\mathfrak{L}}_{1}}}},\overline{\overline{{\mathord{\buildrel{\lower1pt\hbox{$\scriptscriptstyle\smile$}}\over{\mathfrak{W}}}}_{{\mathfrak{L}}_{2}}}},\dots ,\overline{\overline{{\mathord{\buildrel{\lower1pt\hbox{$\scriptscriptstyle\smile$}}\over{\mathfrak{W}}}}_{{\mathfrak{L}}_{\rm Z}}}}\right)\le {\overline{\overline{{\mathord{\buildrel{\lower1pt\hbox{$\scriptscriptstyle\smile$}}\over{\mathfrak{W}}}}_{{\mathfrak{L}}_{\tau }}}}}^{+}$$

### Definition 8

For a group of IVA-IFNs $$\overline{\overline{{\mathord{\buildrel{\lower1pt\hbox{$\scriptscriptstyle\smile$}}\over{\mathfrak{W}}}}_{{\mathfrak{L}}_{\tau }}}}=\left(\left[{\overline{\overline{{\zeta }_{\overline{\overline{{\mathord{\buildrel{\lower1pt\hbox{$\scriptscriptstyle\smile$}}\over{\mathfrak{W}}}}_{{\mathfrak{L}}_{\tau }}}}}}}}^{l},{\overline{\overline{{\zeta }_{\overline{\overline{{\mathord{\buildrel{\lower1pt\hbox{$\scriptscriptstyle\smile$}}\over{\mathfrak{W}}}}_{{\mathfrak{L}}_{\tau }}}}}}}}^{u}\right],\left[{\overline{\overline{{\eta }_{\overline{\overline{{\mathord{\buildrel{\lower1pt\hbox{$\scriptscriptstyle\smile$}}\over{\mathfrak{W}}}}_{{\mathfrak{L}}_{\tau }}}}}}}}^{l},{\overline{\overline{{\eta }_{\overline{\overline{{\mathord{\buildrel{\lower1pt\hbox{$\scriptscriptstyle\smile$}}\over{\mathfrak{W}}}}_{{\mathfrak{L}}_{\tau }}}}}}}}^{u}\right]\right),\tau =\mathrm{1,2},\dots ,{\rm Z},$$ then an IVA-IFAAPOG operator is described as

21 $$IVA-IFAAPOG\left(\overline{\overline{{\mathord{\buildrel{\lower1pt\hbox{$\scriptscriptstyle\smile$}}\over{\mathfrak{W}}}}_{{\mathfrak{L}}_{1}}}},\overline{\overline{{\mathord{\buildrel{\lower1pt\hbox{$\scriptscriptstyle\smile$}}\over{\mathfrak{W}}}}_{{\mathfrak{L}}_{2}}}},\dots ,\overline{\overline{{\mathord{\buildrel{\lower1pt\hbox{$\scriptscriptstyle\smile$}}\over{\mathfrak{W}}}}_{{\mathfrak{L}}_{\rm Z}}}}\right)={\overline{\overline{{\mathord{\buildrel{\lower1pt\hbox{$\scriptscriptstyle\smile$}}\over{\mathfrak{W}}}}_{{\mathfrak{L}}_{0\left(1\right)}}}}}^{\overline{\overline{{\Xi }_{1}}}}\otimes {\overline{\overline{{\mathord{\buildrel{\lower1pt\hbox{$\scriptscriptstyle\smile$}}\over{\mathfrak{W}}}}_{{\mathfrak{L}}_{0\left(2\right)}}}}}^{\overline{\overline{{\Xi }_{2}}}}\otimes \dots \otimes {\overline{\overline{{\mathord{\buildrel{\lower1pt\hbox{$\scriptscriptstyle\smile$}}\over{\mathfrak{W}}}}_{{\mathfrak{L}}_{0\left({\rm Z}\right)}}}}}^{\overline{\overline{{\Xi }_{\rm Z}}}}={\otimes }_{\tau =1}^{\rm Z}\left({\overline{\overline{{\mathord{\buildrel{\lower1pt\hbox{$\scriptscriptstyle\smile$}}\over{\mathfrak{W}}}}_{{\mathfrak{L}}_{0\left(\tau \right)}}}}}^{\overline{\overline{{\Xi }_{\tau }}}}\right)$$

where $$0\left(\tau \right)\le 0\left(\tau -1\right)$$ represents the permutations and $$\overline{\overline{{\Xi }_{\tau }}}=\frac{\left(1+\overline{\overline{\mathfrak{I}}}\left(\overline{\overline{{\mathord{\buildrel{\lower1pt\hbox{$\scriptscriptstyle\smile$}}\over{\mathfrak{W}}}}_{{\mathfrak{L}}_{\tau }}}}\right)\right)}{\sum_{\tau =1}^{\rm Z}\left(1+\overline{\overline{\mathfrak{I}}}\left(\overline{\overline{{\mathord{\buildrel{\lower1pt\hbox{$\scriptscriptstyle\smile$}}\over{\mathfrak{W}}}}_{{\mathfrak{L}}_{\tau }}}}\right)\right)}$$. Further,$$\overline{\overline{\mathfrak{I}}}\left(\overline{\overline{{\mathord{\buildrel{\lower1pt\hbox{$\scriptscriptstyle\smile$}}\over{\mathfrak{W}}}}_{{\mathfrak{L}}_{\tau }}}}\right)=\sum_{\begin{array}{c}s=1,\\ s\ne \tau \end{array}}^{\rm Z}Sup\left(\overline{\overline{{\mathord{\buildrel{\lower1pt\hbox{$\scriptscriptstyle\smile$}}\over{\mathfrak{W}}}}_{{\mathfrak{L}}_{\tau }}}},\overline{\overline{{\mathord{\buildrel{\lower1pt\hbox{$\scriptscriptstyle\smile$}}\over{\mathfrak{W}}}}_{{\mathfrak{L}}_{k}}}}\right),$$ which expresses the relationship between $$\overline{\overline{{\mathord{\buildrel{\lower1pt\hbox{$\scriptscriptstyle\smile$}}\over{\mathfrak{W}}}}_{{\mathfrak{L}}_{\tau }}}}$$ and $$\overline{\overline{{\mathord{\buildrel{\lower1pt\hbox{$\scriptscriptstyle\smile$}}\over{\mathfrak{W}}}}_{{\mathfrak{L}}_{k}}}},$$ where $$Sup\left(\overline{\overline{{\mathord{\buildrel{\lower1pt\hbox{$\scriptscriptstyle\smile$}}\over{\mathfrak{W}}}}_{{\mathfrak{L}}_{\tau }}}},\overline{\overline{{\mathord{\buildrel{\lower1pt\hbox{$\scriptscriptstyle\smile$}}\over{\mathfrak{W}}}}_{{\mathfrak{L}}_{k}}}}\right)=1-Dis\left(\overline{\overline{{\mathord{\buildrel{\lower1pt\hbox{$\scriptscriptstyle\smile$}}\over{\mathfrak{W}}}}_{{\mathfrak{L}}_{\tau }}}},\overline{\overline{{\mathord{\buildrel{\lower1pt\hbox{$\scriptscriptstyle\smile$}}\over{\mathfrak{W}}}}_{{\mathfrak{L}}_{k}}}}\right)$$ and$$Dis\left(\overline{\overline{{\mathord{\buildrel{\lower1pt\hbox{$\scriptscriptstyle\smile$}}\over{\mathfrak{W}}}}_{{\mathfrak{L}}_{\tau }}}},\overline{\overline{{\mathord{\buildrel{\lower1pt\hbox{$\scriptscriptstyle\smile$}}\over{\mathfrak{W}}}}_{{\mathfrak{L}}_{k}}}}\right)=\frac{1}{4}\left(\left|{\overline{\overline{{\zeta }_{\overline{\overline{{\mathord{\buildrel{\lower1pt\hbox{$\scriptscriptstyle\smile$}}\over{\mathfrak{W}}}}_{{\mathfrak{L}}_{\tau }}}}}}}}^{l}-{\overline{\overline{{\zeta }_{\overline{\overline{{\mathord{\buildrel{\lower1pt\hbox{$\scriptscriptstyle\smile$}}\over{\mathfrak{W}}}}_{{\mathfrak{L}}_{k}}}}}}}}^{l}\right|+\left|{\overline{\overline{{\eta }_{\overline{\overline{{\mathord{\buildrel{\lower1pt\hbox{$\scriptscriptstyle\smile$}}\over{\mathfrak{W}}}}_{{\mathfrak{L}}_{\tau }}}}}}}}^{l}-{\overline{\overline{{\eta }_{\overline{\overline{{\mathord{\buildrel{\lower1pt\hbox{$\scriptscriptstyle\smile$}}\over{\mathfrak{W}}}}_{{\mathfrak{L}}_{k}}}}}}}}^{l}\right|+\left|{\overline{\overline{{\zeta }_{\overline{\overline{{\mathord{\buildrel{\lower1pt\hbox{$\scriptscriptstyle\smile$}}\over{\mathfrak{W}}}}_{{\mathfrak{L}}_{\tau }}}}}}}}^{u}-{\overline{\overline{{\zeta }_{\overline{\overline{{\mathord{\buildrel{\lower1pt\hbox{$\scriptscriptstyle\smile$}}\over{\mathfrak{W}}}}_{{\mathfrak{L}}_{k}}}}}}}}^{u}\right|+\left|{\overline{\overline{{\eta }_{\overline{\overline{{\mathord{\buildrel{\lower1pt\hbox{$\scriptscriptstyle\smile$}}\over{\mathfrak{W}}}}_{{\mathfrak{L}}_{\tau }}}}}}}}^{u}-{\overline{\overline{{\eta }_{\overline{\overline{{\mathord{\buildrel{\lower1pt\hbox{$\scriptscriptstyle\smile$}}\over{\mathfrak{W}}}}_{{\mathfrak{L}}_{k}}}}}}}}^{u}\right|\right),$$ stated the value of distance information.

### Theorem 4

For a group of IVA-IFNs $$\overline{\overline{{\mathord{\buildrel{\lower1pt\hbox{$\scriptscriptstyle\smile$}}\over{\mathfrak{W}}}}_{{\mathfrak{L}}_{\tau }}}}=\left(\left[{\overline{\overline{{\zeta }_{\overline{\overline{{\mathord{\buildrel{\lower1pt\hbox{$\scriptscriptstyle\smile$}}\over{\mathfrak{W}}}}_{{\mathfrak{L}}_{\tau }}}}}}}}^{l},{\overline{\overline{{\zeta }_{\overline{\overline{{\mathord{\buildrel{\lower1pt\hbox{$\scriptscriptstyle\smile$}}\over{\mathfrak{W}}}}_{{\mathfrak{L}}_{\tau }}}}}}}}^{u}\right],\left[{\overline{\overline{{\eta }_{\overline{\overline{{\mathord{\buildrel{\lower1pt\hbox{$\scriptscriptstyle\smile$}}\over{\mathfrak{W}}}}_{{\mathfrak{L}}_{\tau }}}}}}}}^{l},{\overline{\overline{{\eta }_{\overline{\overline{{\mathord{\buildrel{\lower1pt\hbox{$\scriptscriptstyle\smile$}}\over{\mathfrak{W}}}}_{{\mathfrak{L}}_{\tau }}}}}}}}^{u}\right]\right),\tau =\mathrm{1,2},\dots ,{\rm Z},$$ the aggregation result from Eq. (21) is still an IVA-IFN, such as

22 $$IVA-IFAAPOG\left(\overline{\overline{{\mathord{\buildrel{\lower1pt\hbox{$\scriptscriptstyle\smile$}}\over{\mathfrak{W}}}}_{{\mathfrak{L}}_{1}}}},\overline{\overline{{\mathord{\buildrel{\lower1pt\hbox{$\scriptscriptstyle\smile$}}\over{\mathfrak{W}}}}_{{\mathfrak{L}}_{2}}}},\dots ,\overline{\overline{{\mathord{\buildrel{\lower1pt\hbox{$\scriptscriptstyle\smile$}}\over{\mathfrak{W}}}}_{{\mathfrak{L}}_{\rm Z}}}}\right)=\left(\begin{array}{c}\left[{e}^{-{\left(\sum_{\tau =1}^{\rm Z}\overline{\overline{{\Xi }_{\tau }}}{\left(-\mathrm{log}\left({\overline{\overline{{\zeta }_{\overline{\overline{{\mathord{\buildrel{\lower1pt\hbox{$\scriptscriptstyle\smile$}}\over{\mathfrak{W}}}}_{{\mathfrak{L}}_{0\left(\tau \right)}}}}}}}}^{l}\right)\right)}^{\Lambda }\right)}^{\frac{1}{\Lambda }}},{e}^{-{\left(\sum_{\tau =1}^{\rm Z}\overline{\overline{{\Xi }_{\tau }}}{\left(-\mathrm{log}\left({\overline{\overline{{\zeta }_{\overline{\overline{{\mathord{\buildrel{\lower1pt\hbox{$\scriptscriptstyle\smile$}}\over{\mathfrak{W}}}}_{{\mathfrak{L}}_{0\left(\tau \right)}}}}}}}}^{u}\right)\right)}^{\Lambda }\right)}^{\frac{1}{\Lambda }}}\right],\\ \left[1-{e}^{-{\left(\sum_{\tau =1}^{\rm Z}\overline{\overline{{\Xi }_{\tau }}}{\left(-\mathrm{log}\left(1-{\overline{\overline{{\eta }_{\overline{\overline{{\mathord{\buildrel{\lower1pt\hbox{$\scriptscriptstyle\smile$}}\over{\mathfrak{W}}}}_{{\mathfrak{L}}_{0\left(\tau \right)}}}}}}}}^{l}\right)\right)}^{\Lambda }\right)}^{\frac{1}{\Lambda }}},1-{e}^{-{\left(\sum_{\tau =1}^{\rm Z}\overline{\overline{{\Xi }_{\tau }}}{\left(-\mathrm{log}\left(1-{\overline{\overline{{\eta }_{\overline{\overline{{\mathord{\buildrel{\lower1pt\hbox{$\scriptscriptstyle\smile$}}\over{\mathfrak{W}}}}_{{\mathfrak{L}}_{0\left(\tau \right)}}}}}}}}^{u}\right)\right)}^{\Lambda }\right)}^{\frac{1}{\Lambda }}}\right]\end{array}\right)$$

### Property 8 

(Idempotency) For a group of IVA-IFNs $$\overline{\overline{{\mathord{\buildrel{\lower1pt\hbox{$\scriptscriptstyle\smile$}}\over{\mathfrak{W}}}}_{{\mathfrak{L}}_{\tau }}}}=\left(\left[{\overline{\overline{{\zeta }_{\overline{\overline{{\mathord{\buildrel{\lower1pt\hbox{$\scriptscriptstyle\smile$}}\over{\mathfrak{W}}}}_{{\mathfrak{L}}_{\tau }}}}}}}}^{l},{\overline{\overline{{\zeta }_{\overline{\overline{{\mathord{\buildrel{\lower1pt\hbox{$\scriptscriptstyle\smile$}}\over{\mathfrak{W}}}}_{{\mathfrak{L}}_{\tau }}}}}}}}^{u}\right],\left[{\overline{\overline{{\eta }_{\overline{\overline{{\mathord{\buildrel{\lower1pt\hbox{$\scriptscriptstyle\smile$}}\over{\mathfrak{W}}}}_{{\mathfrak{L}}_{\tau }}}}}}}}^{l},{\overline{\overline{{\eta }_{\overline{\overline{{\mathord{\buildrel{\lower1pt\hbox{$\scriptscriptstyle\smile$}}\over{\mathfrak{W}}}}_{{\mathfrak{L}}_{\tau }}}}}}}}^{u}\right]\right),\tau =\mathrm{1,2},\dots ,{\rm Z},$$ If $$\overline{\overline{{\mathord{\buildrel{\lower1pt\hbox{$\scriptscriptstyle\smile$}}\over{\mathfrak{W}}}}_{{\mathfrak{L}}_{\tau }}}}=\overline{\overline{{\mathord{\buildrel{\lower1pt\hbox{$\scriptscriptstyle\smile$}}\over{\mathfrak{W}}}}_{\mathfrak{L}}}}=\left(\left[{\overline{\overline{{\zeta }_{\overline{\overline{{\mathord{\buildrel{\lower1pt\hbox{$\scriptscriptstyle\smile$}}\over{\mathfrak{W}}}}_{\mathfrak{L}}}}}}}}^{l},{\overline{\overline{{\zeta }_{\overline{\overline{{\mathord{\buildrel{\lower1pt\hbox{$\scriptscriptstyle\smile$}}\over{\mathfrak{W}}}}_{\mathfrak{L}}}}}}}}^{u}\right],\left[{\overline{\overline{{\eta }_{\overline{\overline{{\mathord{\buildrel{\lower1pt\hbox{$\scriptscriptstyle\smile$}}\over{\mathfrak{W}}}}_{\mathfrak{L}}}}}}}}^{l},{\overline{\overline{{\eta }_{\overline{\overline{{\mathord{\buildrel{\lower1pt\hbox{$\scriptscriptstyle\smile$}}\over{\mathfrak{W}}}}_{\mathfrak{L}}}}}}}}^{u}\right]\right),$$ then

23 $$IVA-IFAAPOG\left(\overline{\overline{{\mathord{\buildrel{\lower1pt\hbox{$\scriptscriptstyle\smile$}}\over{\mathfrak{W}}}}_{{\mathfrak{L}}_{1}}}},\overline{\overline{{\mathord{\buildrel{\lower1pt\hbox{$\scriptscriptstyle\smile$}}\over{\mathfrak{W}}}}_{{\mathfrak{L}}_{2}}}},\dots ,\overline{\overline{{\mathord{\buildrel{\lower1pt\hbox{$\scriptscriptstyle\smile$}}\over{\mathfrak{W}}}}_{{\mathfrak{L}}_{\rm Z}}}}\right)=\overline{\overline{{\mathord{\buildrel{\lower1pt\hbox{$\scriptscriptstyle\smile$}}\over{\mathfrak{W}}}}_{\mathfrak{L}}}}$$

Further, the monotonicity is not kept. Further, we give the boundedness.

### Property 9 

(Boundedness) For a group of IVA-IFNs $$\overline{\overline{{\mathord{\buildrel{\lower1pt\hbox{$\scriptscriptstyle\smile$}}\over{\mathfrak{W}}}}_{{\mathfrak{L}}_{\tau }}}}=\left(\overline{\overline{{\zeta }_{\overline{\overline{{\mathord{\buildrel{\lower1pt\hbox{$\scriptscriptstyle\smile$}}\over{\mathfrak{W}}}}_{{\mathfrak{L}}_{\tau }}}}}}},\overline{\overline{{\eta }_{\overline{\overline{{\mathord{\buildrel{\lower1pt\hbox{$\scriptscriptstyle\smile$}}\over{\mathfrak{W}}}}_{{\mathfrak{L}}_{\tau }}}}}}}\right),\tau =\mathrm{1,2},\dots ,{\rm Z}$$. If $${\overline{\overline{{\mathord{\buildrel{\lower1pt\hbox{$\scriptscriptstyle\smile$}}\over{\mathfrak{W}}}}_{{\mathfrak{L}}_{\tau }}}}}^{-}=\left(\left[\underset{\tau }{\mathrm{min}}{\overline{\overline{{\zeta }_{\overline{\overline{{\mathord{\buildrel{\lower1pt\hbox{$\scriptscriptstyle\smile$}}\over{\mathfrak{W}}}}_{{\mathfrak{L}}_{\tau }}}}}}}}^{l},\underset{\tau }{\mathrm{min}}{\overline{\overline{{\zeta }_{\overline{\overline{{\mathord{\buildrel{\lower1pt\hbox{$\scriptscriptstyle\smile$}}\over{\mathfrak{W}}}}_{{\mathfrak{L}}_{\tau }}}}}}}}^{u}\right],\left[\underset{\tau }{\mathrm{max}}{\overline{\overline{{\eta }_{\overline{\overline{{\mathord{\buildrel{\lower1pt\hbox{$\scriptscriptstyle\smile$}}\over{\mathfrak{W}}}}_{{\mathfrak{L}}_{\tau }}}}}}}}^{l},\underset{\tau }{\mathrm{max}}{\overline{\overline{{\eta }_{\overline{\overline{{\mathord{\buildrel{\lower1pt\hbox{$\scriptscriptstyle\smile$}}\over{\mathfrak{W}}}}_{{\mathfrak{L}}_{\tau }}}}}}}}^{u}\right]\right)$$ and $${\overline{\overline{{\mathord{\buildrel{\lower1pt\hbox{$\scriptscriptstyle\smile$}}\over{\mathfrak{W}}}}_{{\mathfrak{L}}_{\tau }}}}}^{+}=\left(\left[\underset{\tau }{\mathrm{max}}{\overline{\overline{{\zeta }_{\overline{\overline{{\mathord{\buildrel{\lower1pt\hbox{$\scriptscriptstyle\smile$}}\over{\mathfrak{W}}}}_{{\mathfrak{L}}_{\tau }}}}}}}}^{l},\underset{\tau }{\mathrm{max}}{\overline{\overline{{\zeta }_{\overline{\overline{{\mathord{\buildrel{\lower1pt\hbox{$\scriptscriptstyle\smile$}}\over{\mathfrak{W}}}}_{{\mathfrak{L}}_{\tau }}}}}}}}^{u}\right],\left[\underset{\tau }{\mathrm{min}}{\overline{\overline{{\eta }_{\overline{\overline{{\mathord{\buildrel{\lower1pt\hbox{$\scriptscriptstyle\smile$}}\over{\mathfrak{W}}}}_{{\mathfrak{L}}_{\tau }}}}}}}}^{l},\underset{\tau }{\mathrm{min}}{\overline{\overline{{\eta }_{\overline{\overline{{\mathord{\buildrel{\lower1pt\hbox{$\scriptscriptstyle\smile$}}\over{\mathfrak{W}}}}_{{\mathfrak{L}}_{\tau }}}}}}}}^{u}\right]\right),$$ then

$${\overline{\overline{{\mathord{\buildrel{\lower1pt\hbox{$\scriptscriptstyle\smile$}}\over{\mathfrak{W}}}}_{{\mathfrak{L}}_{\tau }}}}}^{-}\le IVA-IFAAPOG\left(\overline{\overline{{\mathord{\buildrel{\lower1pt\hbox{$\scriptscriptstyle\smile$}}\over{\mathfrak{W}}}}_{{\mathfrak{L}}_{1}}}},\overline{\overline{{\mathord{\buildrel{\lower1pt\hbox{$\scriptscriptstyle\smile$}}\over{\mathfrak{W}}}}_{{\mathfrak{L}}_{2}}}},\dots ,\overline{\overline{{\mathord{\buildrel{\lower1pt\hbox{$\scriptscriptstyle\smile$}}\over{\mathfrak{W}}}}_{{\mathfrak{L}}_{\rm Z}}}}\right)\le {\overline{\overline{{\mathord{\buildrel{\lower1pt\hbox{$\scriptscriptstyle\smile$}}\over{\mathfrak{W}}}}_{{\mathfrak{L}}_{\tau }}}}}^{+}$$

## A Decision-Making Procedure Based on the Proposed Operators

The MADM is the subpart of the decision-making process, which is used to determine the best optimal form of the collection of preferences. Based on the derived operators, we develop the MADM technique with the IVA-IF information.

For a MADM problem, there are a finite number of alternatives $$\overline{\overline{{\mathord{\buildrel{\lower1pt\hbox{$\scriptscriptstyle\smile$}}\over{\mathfrak{W}}}}_{{\mathfrak{L}}_{1}}}},\overline{\overline{{\mathord{\buildrel{\lower1pt\hbox{$\scriptscriptstyle\smile$}}\over{\mathfrak{W}}}}_{{\mathfrak{L}}_{2}}}},\dots ,\overline{\overline{{\mathord{\buildrel{\lower1pt\hbox{$\scriptscriptstyle\smile$}}\over{\mathfrak{W}}}}_{{\mathfrak{L}}_{\rm Z}}}}$$ and every alternative is evaluated by a collection of a finite number of attributes $${\overline{\overline{{\mathord{\buildrel{\lower1pt\hbox{$\scriptscriptstyle\smile$}}\over{\mathfrak{W}}}}_{{\mathfrak{L}}_{1}}}}}^{/},{\overline{\overline{{\mathord{\buildrel{\lower1pt\hbox{$\scriptscriptstyle\smile$}}\over{\mathfrak{W}}}}_{{\mathfrak{L}}_{2}}}}}^{/},\dots ,{\overline{\overline{{\mathord{\buildrel{\lower1pt\hbox{$\scriptscriptstyle\smile$}}\over{\mathfrak{W}}}}_{{\mathfrak{L}}_{m}}}}}^{/}$$. To proceed with the above problem, we need to construct a decision matrix $$N={[\overline{\overline{{\mathord{\buildrel{\lower1pt\hbox{$\scriptscriptstyle\smile$}}\over{\mathfrak{W}}}}_{{\mathfrak{L}}_{\tau j}}}}]}_{Z\times m}$$ where $$\overline{\overline{{\mathord{\buildrel{\lower1pt\hbox{$\scriptscriptstyle\smile$}}\over{\mathfrak{W}}}}_{{\mathfrak{L}}_{\tau j}}}}$$ is an evaluation value for the alternative $$\overline{\overline{{\mathord{\buildrel{\lower1pt\hbox{$\scriptscriptstyle\smile$}}\over{\mathfrak{W}}}}_{{\mathfrak{L}}_{\tau }}}}$$ under the attribute $${\overline{\overline{{\mathord{\buildrel{\lower1pt\hbox{$\scriptscriptstyle\smile$}}\over{\mathfrak{W}}}}_{{\mathfrak{L}}_{j}}}}}^{/}$$, which is expressed by an IVA-IF number such as $$\overline{\overline{{\mathord{\buildrel{\lower1pt\hbox{$\scriptscriptstyle\smile$}}\over{\mathfrak{W}}}}_{{\mathfrak{L}}_{\tau j}}}}=\left(\left[{\overline{\overline{{\zeta }_{\overline{\overline{{\mathord{\buildrel{\lower1pt\hbox{$\scriptscriptstyle\smile$}}\over{\mathfrak{W}}}}_{{\mathfrak{L}}_{\tau j}}}}}}}}^{l},{\overline{\overline{{\zeta }_{\overline{\overline{{\mathord{\buildrel{\lower1pt\hbox{$\scriptscriptstyle\smile$}}\over{\mathfrak{W}}}}_{{\mathfrak{L}}_{\tau j}}}}}}}}^{u}\right],\left[{\overline{\overline{{\eta }_{\overline{\overline{{\mathord{\buildrel{\lower1pt\hbox{$\scriptscriptstyle\smile$}}\over{\mathfrak{W}}}}_{{\mathfrak{L}}_{\tau j}}}}}}}}^{l},{\overline{\overline{{\eta }_{\overline{\overline{{\mathord{\buildrel{\lower1pt\hbox{$\scriptscriptstyle\smile$}}\over{\mathfrak{W}}}}_{{\mathfrak{L}}_{\tau j}}}}}}}}^{u}\right]\right),\tau =\mathrm{1,2},\dots ,{\rm Z};j=\mathrm{1,2},\dots ,m$$, where $$\overline{\overline{{\zeta }_{\overline{\overline{{\mathord{\buildrel{\lower1pt\hbox{$\scriptscriptstyle\smile$}}\over{\mathfrak{W}}}}_{{\mathfrak{L}}_{\tau j}}}}}}}\,\, {\text{and}} \,\,\overline{\overline{{\eta }_{\overline{\overline{{\mathord{\buildrel{\lower1pt\hbox{$\scriptscriptstyle\smile$}}\over{\mathfrak{W}}}}_{{\mathfrak{L}}_{\tau j}}}}}}}$$ represent the true value and falsity value, and meet $$0\le {\overline{\overline{{\zeta }_{\overline{\overline{{\mathord{\buildrel{\lower1pt\hbox{$\scriptscriptstyle\smile$}}\over{\mathfrak{W}}}}_{{\mathfrak{L}}_{\tau j}}}}}}}}^{u}+{\overline{\overline{{\eta }_{\overline{\overline{{\mathord{\buildrel{\lower1pt\hbox{$\scriptscriptstyle\smile$}}\over{\mathfrak{W}}}}_{{\mathfrak{L}}_{\tau j}}}}}}}}^{u}\le 1$$. Furthermore, to solve the above problem, we develop the procedure of decision-making based on the proposed operators.

***Step 1:*** Normalize the decision matrix.

Since the evaluation value $$\overline{\overline{{\mathord{\buildrel{\lower1pt\hbox{$\scriptscriptstyle\smile$}}\over{\mathfrak{W}}}}_{{\mathfrak{L}}_{\tau j}}}}$$ for the alternative $$\overline{\overline{{\mathord{\buildrel{\lower1pt\hbox{$\scriptscriptstyle\smile$}}\over{\mathfrak{W}}}}_{{\mathfrak{L}}_{\tau }}}}$$ under the attribute $${\overline{\overline{{\mathord{\buildrel{\lower1pt\hbox{$\scriptscriptstyle\smile$}}\over{\mathfrak{W}}}}_{{\mathfrak{L}}_{j}}}}}^{/}$$ maybe the cost or benefit type should be converted to the same type. If it is cost-type information, then we need to normalize it by

$$N=\left\{\begin{array}{cc}\left(\left[{\overline{\overline{{\zeta }_{\overline{\overline{{\mathord{\buildrel{\lower1pt\hbox{$\scriptscriptstyle\smile$}}\over{\mathfrak{W}}}}_{{\mathfrak{L}}_{\tau j}}}}}}}}^{l},{\overline{\overline{{\zeta }_{\overline{\overline{{\mathord{\buildrel{\lower1pt\hbox{$\scriptscriptstyle\smile$}}\over{\mathfrak{W}}}}_{{\mathfrak{L}}_{\tau j}}}}}}}}^{u}\right],\left[{\overline{\overline{{\eta }_{\overline{\overline{{\mathord{\buildrel{\lower1pt\hbox{$\scriptscriptstyle\smile$}}\over{\mathfrak{W}}}}_{{\mathfrak{L}}_{\tau j}}}}}}}}^{l},{\overline{\overline{{\eta }_{\overline{\overline{{\mathord{\buildrel{\lower1pt\hbox{$\scriptscriptstyle\smile$}}\over{\mathfrak{W}}}}_{{\mathfrak{L}}_{\tau j}}}}}}}}^{u}\right]\right)& for\, benefit\, types\\ \left(\left[{\overline{\overline{{\eta }_{\overline{\overline{{\mathord{\buildrel{\lower1pt\hbox{$\scriptscriptstyle\smile$}}\over{\mathfrak{W}}}}_{{\mathfrak{L}}_{\tau j}}}}}}}}^{l},{\overline{\overline{{\eta }_{\overline{\overline{{\mathord{\buildrel{\lower1pt\hbox{$\scriptscriptstyle\smile$}}\over{\mathfrak{W}}}}_{{\mathfrak{L}}_{\tau j}}}}}}}}^{u}\right],\left[{\overline{\overline{{\zeta }_{\overline{\overline{{\mathord{\buildrel{\lower1pt\hbox{$\scriptscriptstyle\smile$}}\over{\mathfrak{W}}}}_{{\mathfrak{L}}_{\tau j}}}}}}}}^{l},{\overline{\overline{{\zeta }_{\overline{\overline{{\mathord{\buildrel{\lower1pt\hbox{$\scriptscriptstyle\smile$}}\over{\mathfrak{W}}}}_{{\mathfrak{L}}_{\tau j}}}}}}}}^{u}\right]\right)& for\, cost \,types\end{array}\right.$$

***Step 2:*** Aggregate all attribute values of each alternative by the IVA-IFAAPA operator and IVA-IFAAPG operator:

$$\overline{\overline{{\mathord{\buildrel{\lower1pt\hbox{$\scriptscriptstyle\smile$}}\over{\mathfrak{W}}}}_{{\mathfrak{L}}_{\tau }}}}=IVA-IFAAPA\left(\overline{\overline{{\mathord{\buildrel{\lower1pt\hbox{$\scriptscriptstyle\smile$}}\over{\mathfrak{W}}}}_{{\mathfrak{L}}_{\tau 1}}}},\overline{\overline{{\mathord{\buildrel{\lower1pt\hbox{$\scriptscriptstyle\smile$}}\over{\mathfrak{W}}}}_{{\mathfrak{L}}_{\tau 2}}}},\dots ,\overline{\overline{{\mathord{\buildrel{\lower1pt\hbox{$\scriptscriptstyle\smile$}}\over{\mathfrak{W}}}}_{{\mathfrak{L}}_{\tau m}}}}\right)=\left(\begin{array}{c}\left[1-{e}^{-{\left(\sum_{j=1}^{m}\overline{\overline{{\Xi }_{j}}}{\left(-\mathrm{log}\left(1-{\overline{\overline{{\zeta }_{\overline{\overline{{\mathord{\buildrel{\lower1pt\hbox{$\scriptscriptstyle\smile$}}\over{\mathfrak{W}}}}_{{\mathfrak{L}}_{\tau j}}}}}}}}^{l}\right)\right)}^{\Lambda }\right)}^{\frac{1}{\Lambda }}},1-{e}^{-{\left(\sum_{j=1}^{m}\overline{\overline{{\Xi }_{j}}}{\left(-\mathrm{log}\left(1-{\overline{\overline{{\zeta }_{\overline{\overline{{\mathord{\buildrel{\lower1pt\hbox{$\scriptscriptstyle\smile$}}\over{\mathfrak{W}}}}_{{\mathfrak{L}}_{\tau j}}}}}}}}^{u}\right)\right)}^{\Lambda }\right)}^{\frac{1}{\Lambda }}}\right],\\ \left[{e}^{-{\left(\sum_{j=1}^{m}\overline{\overline{{\Xi }_{j}}}{\left(-\mathrm{log}\left({\overline{\overline{{\eta }_{\overline{\overline{{\mathord{\buildrel{\lower1pt\hbox{$\scriptscriptstyle\smile$}}\over{\mathfrak{W}}}}_{{\mathfrak{L}}_{\tau j}}}}}}}}^{l}\right)\right)}^{\Lambda }\right)}^{\frac{1}{\Lambda }}},{e}^{-{\left(\sum_{j=1}^{m}\overline{\overline{{\Xi }_{j}}}{\left(-\mathrm{log}\left({\overline{\overline{{\eta }_{\overline{\overline{{\mathord{\buildrel{\lower1pt\hbox{$\scriptscriptstyle\smile$}}\over{\mathfrak{W}}}}_{{\mathfrak{L}}_{\tau j}}}}}}}}^{u}\right)\right)}^{\Lambda }\right)}^{\frac{1}{\Lambda }}}\right]\end{array}\right)$$

$$\overline{\overline{{\mathord{\buildrel{\lower1pt\hbox{$\scriptscriptstyle\smile$}}\over{\mathfrak{W}}}}_{{\mathfrak{L}}_{\tau }}}}=IVA-IFAAPG\left(\overline{\overline{{\mathord{\buildrel{\lower1pt\hbox{$\scriptscriptstyle\smile$}}\over{\mathfrak{W}}}}_{{\mathfrak{L}}_{\tau 1}}}},\overline{\overline{{\mathord{\buildrel{\lower1pt\hbox{$\scriptscriptstyle\smile$}}\over{\mathfrak{W}}}}_{{\mathfrak{L}}_{\tau 2}}}},\dots ,\overline{\overline{{\mathord{\buildrel{\lower1pt\hbox{$\scriptscriptstyle\smile$}}\over{\mathfrak{W}}}}_{{\mathfrak{L}}_{\tau m}}}}\right)=\left(\begin{array}{c}\left[{e}^{-{\left(\sum_{j=1}^{\rm Z}\overline{\overline{{\Xi }_{j}}}{\left(-\mathrm{log}\left({\overline{\overline{{\zeta }_{\overline{\overline{{\mathord{\buildrel{\lower1pt\hbox{$\scriptscriptstyle\smile$}}\over{\mathfrak{W}}}}_{{\mathfrak{L}}_{\tau j}}}}}}}}^{l}\right)\right)}^{\Lambda }\right)}^{\frac{1}{\Lambda }}},{e}^{-{\left(\sum_{j=1}^{\rm Z}\overline{\overline{{\Xi }_{j}}}{\left(-\mathrm{log}\left({\overline{\overline{{\zeta }_{\overline{\overline{{\mathord{\buildrel{\lower1pt\hbox{$\scriptscriptstyle\smile$}}\over{\mathfrak{W}}}}_{{\mathfrak{L}}_{\tau j}}}}}}}}^{u}\right)\right)}^{\Lambda }\right)}^{\frac{1}{\Lambda }}}\right],\\ \left[1-{e}^{-{\left(\sum_{j=1}^{\rm Z}\overline{\overline{{\Xi }_{j}}}{\left(-\mathrm{log}\left(1-{\overline{\overline{{\eta }_{\overline{\overline{{\mathord{\buildrel{\lower1pt\hbox{$\scriptscriptstyle\smile$}}\over{\mathfrak{W}}}}_{{\mathfrak{L}}_{\tau j}}}}}}}}^{l}\right)\right)}^{\Lambda }\right)}^{\frac{1}{\Lambda }}},1-{e}^{-{\left(\sum_{j=1}^{\rm Z}\overline{\overline{{\Xi }_{j}}}{\left(-\mathrm{log}\left(1-{\overline{\overline{{\eta }_{\overline{\overline{{\mathord{\buildrel{\lower1pt\hbox{$\scriptscriptstyle\smile$}}\over{\mathfrak{W}}}}_{{\mathfrak{L}}_{\tau j}}}}}}}}^{u}\right)\right)}^{\Lambda }\right)}^{\frac{1}{\Lambda }}}\right]\end{array}\right)$$

***Step 3:*** Calculate the score and accuracy functions based on Eqs. (7) and (8).

***Step 4:*** Obtain the ranking results based on the Score value information and also get the best preference from the collection of alternatives.

In addition, we give a counterexample and try to verify the stability and effectiveness of the proposed approaches under the IVA-IF information.

### Illustrative Example

An enterprise wants to invest its money in some valuable business. For this, five different businesses for investment in 2022 to 2030 are explained as $$\overline{\overline{{\mathord{\buildrel{\lower1pt\hbox{$\scriptscriptstyle\smile$}}\over{\mathfrak{W}}}}_{{\mathfrak{L}}_{1}}}}$$: Computer business, $$\overline{\overline{{\mathord{\buildrel{\lower1pt\hbox{$\scriptscriptstyle\smile$}}\over{\mathfrak{W}}}}_{{\mathfrak{L}}_{2}}}}$$: Laptop business, $$\overline{\overline{{\mathord{\buildrel{\lower1pt\hbox{$\scriptscriptstyle\smile$}}\over{\mathfrak{W}}}}_{{\mathfrak{L}}_{3}}}}$$: Mobile business, $$\overline{\overline{{\mathord{\buildrel{\lower1pt\hbox{$\scriptscriptstyle\smile$}}\over{\mathfrak{W}}}}_{{\mathfrak{L}}_{4}}}}$$: Software business, and$$\overline{\overline{{\mathord{\buildrel{\lower1pt\hbox{$\scriptscriptstyle\smile$}}\over{\mathfrak{W}}}}_{{\mathfrak{L}}_{5}}}}$$: Hardware business. To select the best one from the above five businesses, four different features are used to evaluate these alternatives, which are explained as $${\overline{\overline{{\mathord{\buildrel{\lower1pt\hbox{$\scriptscriptstyle\smile$}}\over{\mathfrak{W}}}}_{{\mathfrak{L}}_{1}}}}}^{/}$$: the price, $${\overline{\overline{{\mathord{\buildrel{\lower1pt\hbox{$\scriptscriptstyle\smile$}}\over{\mathfrak{W}}}}_{{\mathfrak{L}}_{2}}}}}^{/}$$: warranty, $${\overline{\overline{{\mathord{\buildrel{\lower1pt\hbox{$\scriptscriptstyle\smile$}}\over{\mathfrak{W}}}}_{{\mathfrak{L}}_{3}}}}}^{/}$$: quality, and $${\overline{\overline{{\mathord{\buildrel{\lower1pt\hbox{$\scriptscriptstyle\smile$}}\over{\mathfrak{W}}}}_{{\mathfrak{L}}_{4}}}}}^{/}$$: social and political impact. To solve this problem, we construct a decision matrix $$N={[\overline{\overline{{\mathord{\buildrel{\lower1pt\hbox{$\scriptscriptstyle\smile$}}\over{\mathfrak{W}}}}_{{\mathfrak{L}}_{\tau j}}}}]}_{Z\times m}$$ (shown in Table 1) where $$\overline{\overline{{\mathord{\buildrel{\lower1pt\hbox{$\scriptscriptstyle\smile$}}\over{\mathfrak{W}}}}_{{\mathfrak{L}}_{\tau j}}}}$$ is an evaluation value for the business $$\overline{\overline{{\mathord{\buildrel{\lower1pt\hbox{$\scriptscriptstyle\smile$}}\over{\mathfrak{W}}}}_{{\mathfrak{L}}_{\tau }}}}$$ under the attribute $${\overline{\overline{{\mathord{\buildrel{\lower1pt\hbox{$\scriptscriptstyle\smile$}}\over{\mathfrak{W}}}}_{{\mathfrak{L}}_{j}}}}}^{/}$$, which is expressed by an IVA-IF number such as $$\overline{\overline{{\mathord{\buildrel{\lower1pt\hbox{$\scriptscriptstyle\smile$}}\over{\mathfrak{W}}}}_{{\mathfrak{L}}_{\tau j}}}}=\left(\left[{\overline{\overline{{\zeta }_{\overline{\overline{{\mathord{\buildrel{\lower1pt\hbox{$\scriptscriptstyle\smile$}}\over{\mathfrak{W}}}}_{{\mathfrak{L}}_{\tau j}}}}}}}}^{l},{\overline{\overline{{\zeta }_{\overline{\overline{{\mathord{\buildrel{\lower1pt\hbox{$\scriptscriptstyle\smile$}}\over{\mathfrak{W}}}}_{{\mathfrak{L}}_{\tau j}}}}}}}}^{u}\right],\left[{\overline{\overline{{\eta }_{\overline{\overline{{\mathord{\buildrel{\lower1pt\hbox{$\scriptscriptstyle\smile$}}\over{\mathfrak{W}}}}_{{\mathfrak{L}}_{\tau j}}}}}}}}^{l},{\overline{\overline{{\eta }_{\overline{\overline{{\mathord{\buildrel{\lower1pt\hbox{$\scriptscriptstyle\smile$}}\over{\mathfrak{W}}}}_{{\mathfrak{L}}_{\tau j}}}}}}}}^{u}\right]\right),\tau =\mathrm{1,2},\dots ,{\rm Z};j=\mathrm{1,2},\dots ,m$$, based on the proposed method, we can solve this problem by the following steps.

***Step 1:*** Normalize the decision matrix.

Since all evaluation values are benefit types of information, it is not needed to normalize them.

Further, based on the data in Table 1, we get their distance measures by $$Dis\left(\overline{\overline{{\mathord{\buildrel{\lower1pt\hbox{$\scriptscriptstyle\smile$}}\over{\mathfrak{W}}}}_{{\mathfrak{L}}_{\tau j}}}},\overline{\overline{{\mathord{\buildrel{\lower1pt\hbox{$\scriptscriptstyle\smile$}}\over{\mathfrak{W}}}}_{{\mathfrak{L}}_{\tau k}}}}\right)=\frac{1}{4}\left(\left|{\overline{\overline{{\zeta }_{\overline{\overline{{\mathord{\buildrel{\lower1pt\hbox{$\scriptscriptstyle\smile$}}\over{\mathfrak{W}}}}_{{\mathfrak{L}}_{\tau j}}}}}}}}^{l}-{\overline{\overline{{\zeta }_{\overline{\overline{{\mathord{\buildrel{\lower1pt\hbox{$\scriptscriptstyle\smile$}}\over{\mathfrak{W}}}}_{{\mathfrak{L}}_{\tau k}}}}}}}}^{l}\right|+\left|{\overline{\overline{{\eta }_{\overline{\overline{{\mathord{\buildrel{\lower1pt\hbox{$\scriptscriptstyle\smile$}}\over{\mathfrak{W}}}}_{{\mathfrak{L}}_{\tau j}}}}}}}}^{l}-{\overline{\overline{{\eta }_{\overline{\overline{{\mathord{\buildrel{\lower1pt\hbox{$\scriptscriptstyle\smile$}}\over{\mathfrak{W}}}}_{{\mathfrak{L}}_{\tau k}}}}}}}}^{l}\right|+\left|{\overline{\overline{{\zeta }_{\overline{\overline{{\mathord{\buildrel{\lower1pt\hbox{$\scriptscriptstyle\smile$}}\over{\mathfrak{W}}}}_{{\mathfrak{L}}_{\tau j}}}}}}}}^{u}-{\overline{\overline{{\zeta }_{\overline{\overline{{\mathord{\buildrel{\lower1pt\hbox{$\scriptscriptstyle\smile$}}\over{\mathfrak{W}}}}_{{\mathfrak{L}}_{\tau k}}}}}}}}^{u}\right|+\left|{\overline{\overline{{\eta }_{\overline{\overline{{\mathord{\buildrel{\lower1pt\hbox{$\scriptscriptstyle\smile$}}\over{\mathfrak{W}}}}_{{\mathfrak{L}}_{\tau j}}}}}}}}^{u}-{\overline{\overline{{\eta }_{\overline{\overline{{\mathord{\buildrel{\lower1pt\hbox{$\scriptscriptstyle\smile$}}\over{\mathfrak{W}}}}_{{\mathfrak{L}}_{\tau k}}}}}}}}^{u}\right|\right)$$, and shown in Table 2.

**Table 1:** Initial IVA-IF decision matrix

	$${\overline{\overline{{\mathord{\buildrel{\lower1pt\hbox{$\scriptscriptstyle\smile$}}\over{\mathfrak{W}}}}_{{\mathfrak{L}}_{1}}}}}^{/}$$	$${\overline{\overline{{\mathord{\buildrel{\lower1pt\hbox{$\scriptscriptstyle\smile$}}\over{\mathfrak{W}}}}_{{\mathfrak{L}}_{2}}}}}^{/}$$	$${\overline{\overline{{\mathord{\buildrel{\lower1pt\hbox{$\scriptscriptstyle\smile$}}\over{\mathfrak{W}}}}_{{\mathfrak{L}}_{3}}}}}^{/}$$	$${\overline{\overline{{\mathord{\buildrel{\lower1pt\hbox{$\scriptscriptstyle\smile$}}\over{\mathfrak{W}}}}_{{\mathfrak{L}}_{4}}}}}^{/}$$
$$\overline{\overline{{\mathord{\buildrel{\lower1pt\hbox{$\scriptscriptstyle\smile$}}\over{\mathfrak{W}}}}_{{\mathfrak{L}}_{1}}}}$$	$$\left(\left[\mathrm{0.3,0.4}\right],\left[\mathrm{0.1,0.2}\right]\right)$$	$$\left(\left[\mathrm{0.31,0.41}\right],\left[\mathrm{0.11,0.21}\right]\right)$$	$$\left(\left[\mathrm{0.32,0.42}\right],\left[\mathrm{0.12,0.22}\right]\right)$$	$$\left(\left[\mathrm{0.33,0.43}\right],\left[\mathrm{0.13,0.23}\right]\right)$$
$$\overline{\overline{{\mathord{\buildrel{\lower1pt\hbox{$\scriptscriptstyle\smile$}}\over{\mathfrak{W}}}}_{{\mathfrak{L}}_{2}}}}$$	$$\left(\left[\mathrm{0.6,0.7}\right],\left[\mathrm{0.1,0.2}\right]\right)$$	$$\left(\left[\mathrm{0.61,0.71}\right],\left[\mathrm{0.11,0.21}\right]\right)$$	$$\left(\left[\mathrm{0.62,0.72}\right],\left[\mathrm{0.12,0.22}\right]\right)$$	$$\left(\left[\mathrm{0.63,0.73}\right],\left[\mathrm{0.13,0.23}\right]\right)$$
$$\overline{\overline{{\mathord{\buildrel{\lower1pt\hbox{$\scriptscriptstyle\smile$}}\over{\mathfrak{W}}}}_{{\mathfrak{L}}_{3}}}}$$	$$\left(\left[\mathrm{0.4,0.5}\right],\left[\mathrm{0.3,0.4}\right]\right)$$	$$\left(\left[\mathrm{0.41,0.51}\right],\left[\mathrm{0.31,0.41}\right]\right)$$	$$\left(\left[\mathrm{0.42,0.52}\right],\left[\mathrm{0.32,0.42}\right]\right)$$	$$\left(\left[\mathrm{0.43,0.53}\right],\left[\mathrm{0.33,0.43}\right]\right)$$
$$\overline{\overline{{\mathord{\buildrel{\lower1pt\hbox{$\scriptscriptstyle\smile$}}\over{\mathfrak{W}}}}_{{\mathfrak{L}}_{4}}}}$$	$$\left(\left[\mathrm{0.5,0.6}\right],\left[\mathrm{0.2,0.3}\right]\right)$$	$$\left(\left[\mathrm{0.51,0.61}\right],\left[\mathrm{0.21,0.31}\right]\right)$$	$$\left(\left[\mathrm{0.52,0.62}\right],\left[\mathrm{0.22,0.32}\right]\right)$$	$$\left(\left[\mathrm{0.53,0.63}\right],\left[\mathrm{0.23,0.33}\right]\right)$$
$$\overline{\overline{{\mathord{\buildrel{\lower1pt\hbox{$\scriptscriptstyle\smile$}}\over{\mathfrak{W}}}}_{{\mathfrak{L}}_{5}}}}$$	$$\left(\left[\mathrm{0.2,0.4}\right],\left[\mathrm{0.3,0.4}\right]\right)$$	$$\left(\left[\mathrm{0.21,0.41}\right],\left[\mathrm{0.31,0.41}\right]\right)$$	$$\left(\left[\mathrm{0.22,0.42}\right],\left[\mathrm{0.32,0.42}\right]\right)$$	$$\left(\left[\mathrm{0.23,0.43}\right],\left[\mathrm{0.33,0.43}\right]\right)$$

**Table 2:** The distance measures

$$Dis\left(\overline{\overline{{\mathord{\buildrel{\lower1pt\hbox{$\scriptscriptstyle\smile$}}\over{\mathfrak{W}}}}_{{\mathfrak{L}}_{11}}}},\overline{\overline{{\mathord{\buildrel{\lower1pt\hbox{$\scriptscriptstyle\smile$}}\over{\mathfrak{W}}}}_{{\mathfrak{L}}_{12}}}}\right)=0.01$$	$$Dis\left(\overline{\overline{{\mathord{\buildrel{\lower1pt\hbox{$\scriptscriptstyle\smile$}}\over{\mathfrak{W}}}}_{{\mathfrak{L}}_{11}}}},\overline{\overline{{\mathord{\buildrel{\lower1pt\hbox{$\scriptscriptstyle\smile$}}\over{\mathfrak{W}}}}_{{\mathfrak{L}}_{12}}}}\right)=0.01$$	$$Dis\left(\overline{\overline{{\mathord{\buildrel{\lower1pt\hbox{$\scriptscriptstyle\smile$}}\over{\mathfrak{W}}}}_{{\mathfrak{L}}_{11}}}},\overline{\overline{{\mathord{\buildrel{\lower1pt\hbox{$\scriptscriptstyle\smile$}}\over{\mathfrak{W}}}}_{{\mathfrak{L}}_{12}}}}\right)=0.01$$	$$Dis\left(\overline{\overline{{\mathord{\buildrel{\lower1pt\hbox{$\scriptscriptstyle\smile$}}\over{\mathfrak{W}}}}_{{\mathfrak{L}}_{11}}}},\overline{\overline{{\mathord{\buildrel{\lower1pt\hbox{$\scriptscriptstyle\smile$}}\over{\mathfrak{W}}}}_{{\mathfrak{L}}_{12}}}}\right)=0.01$$	$$Dis\left(\overline{\overline{{\mathord{\buildrel{\lower1pt\hbox{$\scriptscriptstyle\smile$}}\over{\mathfrak{W}}}}_{{\mathfrak{L}}_{11}}}},\overline{\overline{{\mathord{\buildrel{\lower1pt\hbox{$\scriptscriptstyle\smile$}}\over{\mathfrak{W}}}}_{{\mathfrak{L}}_{12}}}}\right)=0.01$$
$$Dis\left(\overline{\overline{{\mathord{\buildrel{\lower1pt\hbox{$\scriptscriptstyle\smile$}}\over{\mathfrak{W}}}}_{{\mathfrak{L}}_{11}}}},\overline{\overline{{\mathord{\buildrel{\lower1pt\hbox{$\scriptscriptstyle\smile$}}\over{\mathfrak{W}}}}_{{\mathfrak{L}}_{13}}}}\right)=0.04$$	$$Dis\left(\overline{\overline{{\mathord{\buildrel{\lower1pt\hbox{$\scriptscriptstyle\smile$}}\over{\mathfrak{W}}}}_{{\mathfrak{L}}_{11}}}},\overline{\overline{{\mathord{\buildrel{\lower1pt\hbox{$\scriptscriptstyle\smile$}}\over{\mathfrak{W}}}}_{{\mathfrak{L}}_{13}}}}\right)=0.04$$	$$Dis\left(\overline{\overline{{\mathord{\buildrel{\lower1pt\hbox{$\scriptscriptstyle\smile$}}\over{\mathfrak{W}}}}_{{\mathfrak{L}}_{11}}}},\overline{\overline{{\mathord{\buildrel{\lower1pt\hbox{$\scriptscriptstyle\smile$}}\over{\mathfrak{W}}}}_{{\mathfrak{L}}_{13}}}}\right)=0.04$$	$$Dis\left(\overline{\overline{{\mathord{\buildrel{\lower1pt\hbox{$\scriptscriptstyle\smile$}}\over{\mathfrak{W}}}}_{{\mathfrak{L}}_{11}}}},\overline{\overline{{\mathord{\buildrel{\lower1pt\hbox{$\scriptscriptstyle\smile$}}\over{\mathfrak{W}}}}_{{\mathfrak{L}}_{13}}}}\right)=0.04$$	$$Dis\left(\overline{\overline{{\mathord{\buildrel{\lower1pt\hbox{$\scriptscriptstyle\smile$}}\over{\mathfrak{W}}}}_{{\mathfrak{L}}_{11}}}},\overline{\overline{{\mathord{\buildrel{\lower1pt\hbox{$\scriptscriptstyle\smile$}}\over{\mathfrak{W}}}}_{{\mathfrak{L}}_{13}}}}\right)=0.04$$
$$Dis\left(\overline{\overline{{\mathord{\buildrel{\lower1pt\hbox{$\scriptscriptstyle\smile$}}\over{\mathfrak{W}}}}_{{\mathfrak{L}}_{11}}}},\overline{\overline{{\mathord{\buildrel{\lower1pt\hbox{$\scriptscriptstyle\smile$}}\over{\mathfrak{W}}}}_{{\mathfrak{L}}_{14}}}}\right)=0.06$$	$$Dis\left(\overline{\overline{{\mathord{\buildrel{\lower1pt\hbox{$\scriptscriptstyle\smile$}}\over{\mathfrak{W}}}}_{{\mathfrak{L}}_{11}}}},\overline{\overline{{\mathord{\buildrel{\lower1pt\hbox{$\scriptscriptstyle\smile$}}\over{\mathfrak{W}}}}_{{\mathfrak{L}}_{14}}}}\right)=0.06$$	$$Dis\left(\overline{\overline{{\mathord{\buildrel{\lower1pt\hbox{$\scriptscriptstyle\smile$}}\over{\mathfrak{W}}}}_{{\mathfrak{L}}_{11}}}},\overline{\overline{{\mathord{\buildrel{\lower1pt\hbox{$\scriptscriptstyle\smile$}}\over{\mathfrak{W}}}}_{{\mathfrak{L}}_{14}}}}\right)=0.06$$	$$Dis\left(\overline{\overline{{\mathord{\buildrel{\lower1pt\hbox{$\scriptscriptstyle\smile$}}\over{\mathfrak{W}}}}_{{\mathfrak{L}}_{11}}}},\overline{\overline{{\mathord{\buildrel{\lower1pt\hbox{$\scriptscriptstyle\smile$}}\over{\mathfrak{W}}}}_{{\mathfrak{L}}_{14}}}}\right)=0.06$$	$$Dis\left(\overline{\overline{{\mathord{\buildrel{\lower1pt\hbox{$\scriptscriptstyle\smile$}}\over{\mathfrak{W}}}}_{{\mathfrak{L}}_{11}}}},\overline{\overline{{\mathord{\buildrel{\lower1pt\hbox{$\scriptscriptstyle\smile$}}\over{\mathfrak{W}}}}_{{\mathfrak{L}}_{14}}}}\right)=0.06$$
$$Dis\left(\overline{\overline{{\mathord{\buildrel{\lower1pt\hbox{$\scriptscriptstyle\smile$}}\over{\mathfrak{W}}}}_{{\mathfrak{L}}_{12}}}},\overline{\overline{{\mathord{\buildrel{\lower1pt\hbox{$\scriptscriptstyle\smile$}}\over{\mathfrak{W}}}}_{{\mathfrak{L}}_{13}}}}\right)=0.02$$	$$Dis\left(\overline{\overline{{\mathord{\buildrel{\lower1pt\hbox{$\scriptscriptstyle\smile$}}\over{\mathfrak{W}}}}_{{\mathfrak{L}}_{12}}}},\overline{\overline{{\mathord{\buildrel{\lower1pt\hbox{$\scriptscriptstyle\smile$}}\over{\mathfrak{W}}}}_{{\mathfrak{L}}_{13}}}}\right)=0.02$$	$$Dis\left(\overline{\overline{{\mathord{\buildrel{\lower1pt\hbox{$\scriptscriptstyle\smile$}}\over{\mathfrak{W}}}}_{{\mathfrak{L}}_{12}}}},\overline{\overline{{\mathord{\buildrel{\lower1pt\hbox{$\scriptscriptstyle\smile$}}\over{\mathfrak{W}}}}_{{\mathfrak{L}}_{13}}}}\right)=0.02$$	$$Dis\left(\overline{\overline{{\mathord{\buildrel{\lower1pt\hbox{$\scriptscriptstyle\smile$}}\over{\mathfrak{W}}}}_{{\mathfrak{L}}_{12}}}},\overline{\overline{{\mathord{\buildrel{\lower1pt\hbox{$\scriptscriptstyle\smile$}}\over{\mathfrak{W}}}}_{{\mathfrak{L}}_{13}}}}\right)=0.02$$	$$Dis\left(\overline{\overline{{\mathord{\buildrel{\lower1pt\hbox{$\scriptscriptstyle\smile$}}\over{\mathfrak{W}}}}_{{\mathfrak{L}}_{12}}}},\overline{\overline{{\mathord{\buildrel{\lower1pt\hbox{$\scriptscriptstyle\smile$}}\over{\mathfrak{W}}}}_{{\mathfrak{L}}_{13}}}}\right)=0.02$$
$$Dis\left(\overline{\overline{{\mathord{\buildrel{\lower1pt\hbox{$\scriptscriptstyle\smile$}}\over{\mathfrak{W}}}}_{{\mathfrak{L}}_{12}}}},\overline{\overline{{\mathord{\buildrel{\lower1pt\hbox{$\scriptscriptstyle\smile$}}\over{\mathfrak{W}}}}_{{\mathfrak{L}}_{14}}}}\right)=0.04$$	$$Dis\left(\overline{\overline{{\mathord{\buildrel{\lower1pt\hbox{$\scriptscriptstyle\smile$}}\over{\mathfrak{W}}}}_{{\mathfrak{L}}_{12}}}},\overline{\overline{{\mathord{\buildrel{\lower1pt\hbox{$\scriptscriptstyle\smile$}}\over{\mathfrak{W}}}}_{{\mathfrak{L}}_{14}}}}\right)=0.04$$	$$Dis\left(\overline{\overline{{\mathord{\buildrel{\lower1pt\hbox{$\scriptscriptstyle\smile$}}\over{\mathfrak{W}}}}_{{\mathfrak{L}}_{12}}}},\overline{\overline{{\mathord{\buildrel{\lower1pt\hbox{$\scriptscriptstyle\smile$}}\over{\mathfrak{W}}}}_{{\mathfrak{L}}_{14}}}}\right)=0.04$$	$$Dis\left(\overline{\overline{{\mathord{\buildrel{\lower1pt\hbox{$\scriptscriptstyle\smile$}}\over{\mathfrak{W}}}}_{{\mathfrak{L}}_{12}}}},\overline{\overline{{\mathord{\buildrel{\lower1pt\hbox{$\scriptscriptstyle\smile$}}\over{\mathfrak{W}}}}_{{\mathfrak{L}}_{14}}}}\right)=0.04$$	$$Dis\left(\overline{\overline{{\mathord{\buildrel{\lower1pt\hbox{$\scriptscriptstyle\smile$}}\over{\mathfrak{W}}}}_{{\mathfrak{L}}_{12}}}},\overline{\overline{{\mathord{\buildrel{\lower1pt\hbox{$\scriptscriptstyle\smile$}}\over{\mathfrak{W}}}}_{{\mathfrak{L}}_{14}}}}\right)=0.04$$
$$Dis\left(\overline{\overline{{\mathord{\buildrel{\lower1pt\hbox{$\scriptscriptstyle\smile$}}\over{\mathfrak{W}}}}_{{\mathfrak{L}}_{13}}}},\overline{\overline{{\mathord{\buildrel{\lower1pt\hbox{$\scriptscriptstyle\smile$}}\over{\mathfrak{W}}}}_{{\mathfrak{L}}_{14}}}}\right)=0.02$$	$$Dis\left(\overline{\overline{{\mathord{\buildrel{\lower1pt\hbox{$\scriptscriptstyle\smile$}}\over{\mathfrak{W}}}}_{{\mathfrak{L}}_{13}}}},\overline{\overline{{\mathord{\buildrel{\lower1pt\hbox{$\scriptscriptstyle\smile$}}\over{\mathfrak{W}}}}_{{\mathfrak{L}}_{14}}}}\right)=0.02$$	$$Dis\left(\overline{\overline{{\mathord{\buildrel{\lower1pt\hbox{$\scriptscriptstyle\smile$}}\over{\mathfrak{W}}}}_{{\mathfrak{L}}_{13}}}},\overline{\overline{{\mathord{\buildrel{\lower1pt\hbox{$\scriptscriptstyle\smile$}}\over{\mathfrak{W}}}}_{{\mathfrak{L}}_{14}}}}\right)=0.02$$	$$Dis\left(\overline{\overline{{\mathord{\buildrel{\lower1pt\hbox{$\scriptscriptstyle\smile$}}\over{\mathfrak{W}}}}_{{\mathfrak{L}}_{13}}}},\overline{\overline{{\mathord{\buildrel{\lower1pt\hbox{$\scriptscriptstyle\smile$}}\over{\mathfrak{W}}}}_{{\mathfrak{L}}_{14}}}}\right)=0.02$$	$$Dis\left(\overline{\overline{{\mathord{\buildrel{\lower1pt\hbox{$\scriptscriptstyle\smile$}}\over{\mathfrak{W}}}}_{{\mathfrak{L}}_{13}}}},\overline{\overline{{\mathord{\buildrel{\lower1pt\hbox{$\scriptscriptstyle\smile$}}\over{\mathfrak{W}}}}_{{\mathfrak{L}}_{14}}}}\right)=0.02$$

Further, we calculate their support degrees using the data in Table 2 based on $$Sup\left(\overline{\overline{{\mathord{\buildrel{\lower1pt\hbox{$\scriptscriptstyle\smile$}}\over{\mathfrak{W}}}}_{{\mathfrak{L}}_{\tau j}}}},\overline{\overline{{\mathord{\buildrel{\lower1pt\hbox{$\scriptscriptstyle\smile$}}\over{\mathfrak{W}}}}_{{\mathfrak{L}}_{\tau k}}}}\right)=1-Dis\left(\overline{\overline{{\mathord{\buildrel{\lower1pt\hbox{$\scriptscriptstyle\smile$}}\over{\mathfrak{W}}}}_{{\mathfrak{L}}_{\tau j}}}},\overline{\overline{{\mathord{\buildrel{\lower1pt\hbox{$\scriptscriptstyle\smile$}}\over{\mathfrak{W}}}}_{{\mathfrak{L}}_{\tau k}}}}\right)=1-\frac{1}{4}\left(\left|{\overline{\overline{{\zeta }_{\overline{\overline{{\mathord{\buildrel{\lower1pt\hbox{$\scriptscriptstyle\smile$}}\over{\mathfrak{W}}}}_{{\mathfrak{L}}_{\tau j}}}}}}}}^{l}-{\overline{\overline{{\zeta }_{\overline{\overline{{\mathord{\buildrel{\lower1pt\hbox{$\scriptscriptstyle\smile$}}\over{\mathfrak{W}}}}_{{\mathfrak{L}}_{\tau k}}}}}}}}^{l}\right|+\left|{\overline{\overline{{\eta }_{\overline{\overline{{\mathord{\buildrel{\lower1pt\hbox{$\scriptscriptstyle\smile$}}\over{\mathfrak{W}}}}_{{\mathfrak{L}}_{\tau j}}}}}}}}^{l}-{\overline{\overline{{\eta }_{\overline{\overline{{\mathord{\buildrel{\lower1pt\hbox{$\scriptscriptstyle\smile$}}\over{\mathfrak{W}}}}_{{\mathfrak{L}}_{\tau k}}}}}}}}^{l}\right|+\left|{\overline{\overline{{\zeta }_{\overline{\overline{{\mathord{\buildrel{\lower1pt\hbox{$\scriptscriptstyle\smile$}}\over{\mathfrak{W}}}}_{{\mathfrak{L}}_{\tau j}}}}}}}}^{u}-{\overline{\overline{{\zeta }_{\overline{\overline{{\mathord{\buildrel{\lower1pt\hbox{$\scriptscriptstyle\smile$}}\over{\mathfrak{W}}}}_{{\mathfrak{L}}_{\tau k}}}}}}}}^{u}\right|+\left|{\overline{\overline{{\eta }_{\overline{\overline{{\mathord{\buildrel{\lower1pt\hbox{$\scriptscriptstyle\smile$}}\over{\mathfrak{W}}}}_{{\mathfrak{L}}_{\tau j}}}}}}}}^{u}-{\overline{\overline{{\eta }_{\overline{\overline{{\mathord{\buildrel{\lower1pt\hbox{$\scriptscriptstyle\smile$}}\over{\mathfrak{W}}}}_{{\mathfrak{L}}_{\tau k}}}}}}}}^{u}\right|\right)$$, and the results are shown in Table 3.

**Table 3:** The support degrees

$${\varvec{S}}{\varvec{u}}{\varvec{p}}\left(\overline{\overline{{\mathord{\buildrel{\lower1pt\hbox{$\scriptscriptstyle\smile$}}\over{\mathfrak{W}}}}_{{\mathfrak{L}}_{11}}}},\overline{\overline{{\mathord{\buildrel{\lower1pt\hbox{$\scriptscriptstyle\smile$}}\over{\mathfrak{W}}}}_{{\mathfrak{L}}_{12}}}}\right)=0.99$$	$${\varvec{S}}{\varvec{u}}{\varvec{p}}\left(\overline{\overline{{\mathord{\buildrel{\lower1pt\hbox{$\scriptscriptstyle\smile$}}\over{\mathfrak{W}}}}_{{\mathfrak{L}}_{11}}}},\overline{\overline{{\mathord{\buildrel{\lower1pt\hbox{$\scriptscriptstyle\smile$}}\over{\mathfrak{W}}}}_{{\mathfrak{L}}_{12}}}}\right)=0.99$$	$${\varvec{S}}{\varvec{u}}{\varvec{p}}\left(\overline{\overline{{\mathord{\buildrel{\lower1pt\hbox{$\scriptscriptstyle\smile$}}\over{\mathfrak{W}}}}_{{\mathfrak{L}}_{11}}}},\overline{\overline{{\mathord{\buildrel{\lower1pt\hbox{$\scriptscriptstyle\smile$}}\over{\mathfrak{W}}}}_{{\mathfrak{L}}_{12}}}}\right)=0.99$$	$${\varvec{S}}{\varvec{u}}{\varvec{p}}\left(\overline{\overline{{\mathord{\buildrel{\lower1pt\hbox{$\scriptscriptstyle\smile$}}\over{\mathfrak{W}}}}_{{\mathfrak{L}}_{11}}}},\overline{\overline{{\mathord{\buildrel{\lower1pt\hbox{$\scriptscriptstyle\smile$}}\over{\mathfrak{W}}}}_{{\mathfrak{L}}_{12}}}}\right)=0.99$$	$${\varvec{S}}{\varvec{u}}{\varvec{p}}\left(\overline{\overline{{\mathord{\buildrel{\lower1pt\hbox{$\scriptscriptstyle\smile$}}\over{\mathfrak{W}}}}_{{\mathfrak{L}}_{11}}}},\overline{\overline{{\mathord{\buildrel{\lower1pt\hbox{$\scriptscriptstyle\smile$}}\over{\mathfrak{W}}}}_{{\mathfrak{L}}_{12}}}}\right)=0.99$$
$${\varvec{S}}{\varvec{u}}{\varvec{p}}\left(\overline{\overline{{\mathord{\buildrel{\lower1pt\hbox{$\scriptscriptstyle\smile$}}\over{\mathfrak{W}}}}_{{\mathfrak{L}}_{11}}}},\overline{\overline{{\mathord{\buildrel{\lower1pt\hbox{$\scriptscriptstyle\smile$}}\over{\mathfrak{W}}}}_{{\mathfrak{L}}_{13}}}}\right)=0.96$$	$${\varvec{S}}{\varvec{u}}{\varvec{p}}\left(\overline{\overline{{\mathord{\buildrel{\lower1pt\hbox{$\scriptscriptstyle\smile$}}\over{\mathfrak{W}}}}_{{\mathfrak{L}}_{11}}}},\overline{\overline{{\mathord{\buildrel{\lower1pt\hbox{$\scriptscriptstyle\smile$}}\over{\mathfrak{W}}}}_{{\mathfrak{L}}_{13}}}}\right)=0.96$$	$${\varvec{S}}{\varvec{u}}{\varvec{p}}\left(\overline{\overline{{\mathord{\buildrel{\lower1pt\hbox{$\scriptscriptstyle\smile$}}\over{\mathfrak{W}}}}_{{\mathfrak{L}}_{11}}}},\overline{\overline{{\mathord{\buildrel{\lower1pt\hbox{$\scriptscriptstyle\smile$}}\over{\mathfrak{W}}}}_{{\mathfrak{L}}_{13}}}}\right)=0.96$$	$${\varvec{S}}{\varvec{u}}{\varvec{p}}\left(\overline{\overline{{\mathord{\buildrel{\lower1pt\hbox{$\scriptscriptstyle\smile$}}\over{\mathfrak{W}}}}_{{\mathfrak{L}}_{11}}}},\overline{\overline{{\mathord{\buildrel{\lower1pt\hbox{$\scriptscriptstyle\smile$}}\over{\mathfrak{W}}}}_{{\mathfrak{L}}_{13}}}}\right)=0.96$$	$${\varvec{S}}{\varvec{u}}{\varvec{p}}\left(\overline{\overline{{\mathord{\buildrel{\lower1pt\hbox{$\scriptscriptstyle\smile$}}\over{\mathfrak{W}}}}_{{\mathfrak{L}}_{11}}}},\overline{\overline{{\mathord{\buildrel{\lower1pt\hbox{$\scriptscriptstyle\smile$}}\over{\mathfrak{W}}}}_{{\mathfrak{L}}_{13}}}}\right)=0.96$$
$${\varvec{S}}{\varvec{u}}{\varvec{p}}\left(\overline{\overline{{\mathord{\buildrel{\lower1pt\hbox{$\scriptscriptstyle\smile$}}\over{\mathfrak{W}}}}_{{\mathfrak{L}}_{11}}}},\overline{\overline{{\mathord{\buildrel{\lower1pt\hbox{$\scriptscriptstyle\smile$}}\over{\mathfrak{W}}}}_{{\mathfrak{L}}_{14}}}}\right)=0.94$$	$${\varvec{S}}{\varvec{u}}{\varvec{p}}\left(\overline{\overline{{\mathord{\buildrel{\lower1pt\hbox{$\scriptscriptstyle\smile$}}\over{\mathfrak{W}}}}_{{\mathfrak{L}}_{11}}}},\overline{\overline{{\mathord{\buildrel{\lower1pt\hbox{$\scriptscriptstyle\smile$}}\over{\mathfrak{W}}}}_{{\mathfrak{L}}_{14}}}}\right)=0.94$$	$${\varvec{S}}{\varvec{u}}{\varvec{p}}\left(\overline{\overline{{\mathord{\buildrel{\lower1pt\hbox{$\scriptscriptstyle\smile$}}\over{\mathfrak{W}}}}_{{\mathfrak{L}}_{11}}}},\overline{\overline{{\mathord{\buildrel{\lower1pt\hbox{$\scriptscriptstyle\smile$}}\over{\mathfrak{W}}}}_{{\mathfrak{L}}_{14}}}}\right)=0.94$$	$${\varvec{S}}{\varvec{u}}{\varvec{p}}\left(\overline{\overline{{\mathord{\buildrel{\lower1pt\hbox{$\scriptscriptstyle\smile$}}\over{\mathfrak{W}}}}_{{\mathfrak{L}}_{11}}}},\overline{\overline{{\mathord{\buildrel{\lower1pt\hbox{$\scriptscriptstyle\smile$}}\over{\mathfrak{W}}}}_{{\mathfrak{L}}_{14}}}}\right)=0.94$$	$${\varvec{S}}{\varvec{u}}{\varvec{p}}\left(\overline{\overline{{\mathord{\buildrel{\lower1pt\hbox{$\scriptscriptstyle\smile$}}\over{\mathfrak{W}}}}_{{\mathfrak{L}}_{11}}}},\overline{\overline{{\mathord{\buildrel{\lower1pt\hbox{$\scriptscriptstyle\smile$}}\over{\mathfrak{W}}}}_{{\mathfrak{L}}_{14}}}}\right)=0.94$$
$${\varvec{S}}{\varvec{u}}{\varvec{p}}\left(\overline{\overline{{\mathord{\buildrel{\lower1pt\hbox{$\scriptscriptstyle\smile$}}\over{\mathfrak{W}}}}_{{\mathfrak{L}}_{12}}}},\overline{\overline{{\mathord{\buildrel{\lower1pt\hbox{$\scriptscriptstyle\smile$}}\over{\mathfrak{W}}}}_{{\mathfrak{L}}_{13}}}}\right)=0.98$$	$${\varvec{S}}{\varvec{u}}{\varvec{p}}\left(\overline{\overline{{\mathord{\buildrel{\lower1pt\hbox{$\scriptscriptstyle\smile$}}\over{\mathfrak{W}}}}_{{\mathfrak{L}}_{12}}}},\overline{\overline{{\mathord{\buildrel{\lower1pt\hbox{$\scriptscriptstyle\smile$}}\over{\mathfrak{W}}}}_{{\mathfrak{L}}_{13}}}}\right)=0.98$$	$${\varvec{S}}{\varvec{u}}{\varvec{p}}\left(\overline{\overline{{\mathord{\buildrel{\lower1pt\hbox{$\scriptscriptstyle\smile$}}\over{\mathfrak{W}}}}_{{\mathfrak{L}}_{12}}}},\overline{\overline{{\mathord{\buildrel{\lower1pt\hbox{$\scriptscriptstyle\smile$}}\over{\mathfrak{W}}}}_{{\mathfrak{L}}_{13}}}}\right)=0.98$$	$${\varvec{S}}{\varvec{u}}{\varvec{p}}\left(\overline{\overline{{\mathord{\buildrel{\lower1pt\hbox{$\scriptscriptstyle\smile$}}\over{\mathfrak{W}}}}_{{\mathfrak{L}}_{12}}}},\overline{\overline{{\mathord{\buildrel{\lower1pt\hbox{$\scriptscriptstyle\smile$}}\over{\mathfrak{W}}}}_{{\mathfrak{L}}_{13}}}}\right)=0.98$$	$${\varvec{S}}{\varvec{u}}{\varvec{p}}\left(\overline{\overline{{\mathord{\buildrel{\lower1pt\hbox{$\scriptscriptstyle\smile$}}\over{\mathfrak{W}}}}_{{\mathfrak{L}}_{12}}}},\overline{\overline{{\mathord{\buildrel{\lower1pt\hbox{$\scriptscriptstyle\smile$}}\over{\mathfrak{W}}}}_{{\mathfrak{L}}_{13}}}}\right)=0.98$$
$${\varvec{S}}{\varvec{u}}{\varvec{p}}\left(\overline{\overline{{\mathord{\buildrel{\lower1pt\hbox{$\scriptscriptstyle\smile$}}\over{\mathfrak{W}}}}_{{\mathfrak{L}}_{12}}}},\overline{\overline{{\mathord{\buildrel{\lower1pt\hbox{$\scriptscriptstyle\smile$}}\over{\mathfrak{W}}}}_{{\mathfrak{L}}_{14}}}}\right)=0.96$$	$${\varvec{S}}{\varvec{u}}{\varvec{p}}\left(\overline{\overline{{\mathord{\buildrel{\lower1pt\hbox{$\scriptscriptstyle\smile$}}\over{\mathfrak{W}}}}_{{\mathfrak{L}}_{12}}}},\overline{\overline{{\mathord{\buildrel{\lower1pt\hbox{$\scriptscriptstyle\smile$}}\over{\mathfrak{W}}}}_{{\mathfrak{L}}_{14}}}}\right)=0.96$$	$${\varvec{S}}{\varvec{u}}{\varvec{p}}\left(\overline{\overline{{\mathord{\buildrel{\lower1pt\hbox{$\scriptscriptstyle\smile$}}\over{\mathfrak{W}}}}_{{\mathfrak{L}}_{12}}}},\overline{\overline{{\mathord{\buildrel{\lower1pt\hbox{$\scriptscriptstyle\smile$}}\over{\mathfrak{W}}}}_{{\mathfrak{L}}_{14}}}}\right)=0.96$$	$${\varvec{S}}{\varvec{u}}{\varvec{p}}\left(\overline{\overline{{\mathord{\buildrel{\lower1pt\hbox{$\scriptscriptstyle\smile$}}\over{\mathfrak{W}}}}_{{\mathfrak{L}}_{12}}}},\overline{\overline{{\mathord{\buildrel{\lower1pt\hbox{$\scriptscriptstyle\smile$}}\over{\mathfrak{W}}}}_{{\mathfrak{L}}_{14}}}}\right)=0.96$$	$${\varvec{S}}{\varvec{u}}{\varvec{p}}\left(\overline{\overline{{\mathord{\buildrel{\lower1pt\hbox{$\scriptscriptstyle\smile$}}\over{\mathfrak{W}}}}_{{\mathfrak{L}}_{12}}}},\overline{\overline{{\mathord{\buildrel{\lower1pt\hbox{$\scriptscriptstyle\smile$}}\over{\mathfrak{W}}}}_{{\mathfrak{L}}_{14}}}}\right)=0.96$$
$${\varvec{S}}{\varvec{u}}{\varvec{p}}\left(\overline{\overline{{\mathord{\buildrel{\lower1pt\hbox{$\scriptscriptstyle\smile$}}\over{\mathfrak{W}}}}_{{\mathfrak{L}}_{13}}}},\overline{\overline{{\mathord{\buildrel{\lower1pt\hbox{$\scriptscriptstyle\smile$}}\over{\mathfrak{W}}}}_{{\mathfrak{L}}_{14}}}}\right)=0.98$$	$${\varvec{S}}{\varvec{u}}{\varvec{p}}\left(\overline{\overline{{\mathord{\buildrel{\lower1pt\hbox{$\scriptscriptstyle\smile$}}\over{\mathfrak{W}}}}_{{\mathfrak{L}}_{13}}}},\overline{\overline{{\mathord{\buildrel{\lower1pt\hbox{$\scriptscriptstyle\smile$}}\over{\mathfrak{W}}}}_{{\mathfrak{L}}_{14}}}}\right)=0.98$$	$${\varvec{S}}{\varvec{u}}{\varvec{p}}\left(\overline{\overline{{\mathord{\buildrel{\lower1pt\hbox{$\scriptscriptstyle\smile$}}\over{\mathfrak{W}}}}_{{\mathfrak{L}}_{13}}}},\overline{\overline{{\mathord{\buildrel{\lower1pt\hbox{$\scriptscriptstyle\smile$}}\over{\mathfrak{W}}}}_{{\mathfrak{L}}_{14}}}}\right)=0.98$$	$${\varvec{S}}{\varvec{u}}{\varvec{p}}\left(\overline{\overline{{\mathord{\buildrel{\lower1pt\hbox{$\scriptscriptstyle\smile$}}\over{\mathfrak{W}}}}_{{\mathfrak{L}}_{13}}}},\overline{\overline{{\mathord{\buildrel{\lower1pt\hbox{$\scriptscriptstyle\smile$}}\over{\mathfrak{W}}}}_{{\mathfrak{L}}_{14}}}}\right)=0.98$$	$${\varvec{S}}{\varvec{u}}{\varvec{p}}\left(\overline{\overline{{\mathord{\buildrel{\lower1pt\hbox{$\scriptscriptstyle\smile$}}\over{\mathfrak{W}}}}_{{\mathfrak{L}}_{13}}}},\overline{\overline{{\mathord{\buildrel{\lower1pt\hbox{$\scriptscriptstyle\smile$}}\over{\mathfrak{W}}}}_{{\mathfrak{L}}_{14}}}}\right)=0.98$$

To evaluate the values of $$\overline{\overline{{\Xi }_{\tau j}}}$$, we need to compute the values of $$\overline{\overline{\mathfrak{I}}}\left(\overline{\overline{{\mathord{\buildrel{\lower1pt\hbox{$\scriptscriptstyle\smile$}}\over{\mathfrak{W}}}}_{{\mathfrak{L}}_{\tau j}}}}\right)$$ by $$\overline{\overline{\mathfrak{I}}}\left(\overline{\overline{{\mathord{\buildrel{\lower1pt\hbox{$\scriptscriptstyle\smile$}}\over{\mathfrak{W}}}}_{{\mathfrak{L}}_{\tau j}}}}\right)=\sum_{\begin{array}{c}k=1,\\ k\ne j\end{array}}^{m}Sup\left(\overline{\overline{{\mathord{\buildrel{\lower1pt\hbox{$\scriptscriptstyle\smile$}}\over{\mathfrak{W}}}}_{{\mathfrak{L}}_{\tau j}}}},\overline{\overline{{\mathord{\buildrel{\lower1pt\hbox{$\scriptscriptstyle\smile$}}\over{\mathfrak{W}}}}_{{\mathfrak{L}}_{\tau k}}}}\right)$$, and the results as shown in Table 4.

**Table 4:** The values of $$\overline{\overline{\mathfrak{I}}}\left(\overline{\overline{{\mathord{\buildrel{\lower1pt\hbox{$\scriptscriptstyle\smile$}}\over{\mathfrak{W}}}}_{{\mathfrak{L}}_{\tau j}}}}\right)$$

$$\overline{\overline{{\mathord{\buildrel{\lower1pt\hbox{$\scriptscriptstyle\smile$}}\over{\mathfrak{W}}}}_{{\mathfrak{L}}_{1}}}}$$	$$\overline{\overline{{\mathord{\buildrel{\lower1pt\hbox{$\scriptscriptstyle\smile$}}\over{\mathfrak{W}}}}_{{\mathfrak{L}}_{2}}}}$$	$$\overline{\overline{{\mathord{\buildrel{\lower1pt\hbox{$\scriptscriptstyle\smile$}}\over{\mathfrak{W}}}}_{{\mathfrak{L}}_{3}}}}$$	$$\overline{\overline{{\mathord{\buildrel{\lower1pt\hbox{$\scriptscriptstyle\smile$}}\over{\mathfrak{W}}}}_{{\mathfrak{L}}_{4}}}}$$	$$\overline{\overline{{\mathord{\buildrel{\lower1pt\hbox{$\scriptscriptstyle\smile$}}\over{\mathfrak{W}}}}_{{\mathfrak{L}}_{5}}}}$$
$$\overline{\overline{\mathfrak{I}}}\left(\overline{\overline{{\mathord{\buildrel{\lower1pt\hbox{$\scriptscriptstyle\smile$}}\over{\mathfrak{W}}}}_{{\mathfrak{L}}_{11}}}}\right)=2.89$$	$$\overline{\overline{\mathfrak{I}}}\left(\overline{\overline{{\mathord{\buildrel{\lower1pt\hbox{$\scriptscriptstyle\smile$}}\over{\mathfrak{W}}}}_{{\mathfrak{L}}_{11}}}}\right)=2.89$$	$$\overline{\overline{\mathfrak{I}}}\left(\overline{\overline{{\mathord{\buildrel{\lower1pt\hbox{$\scriptscriptstyle\smile$}}\over{\mathfrak{W}}}}_{{\mathfrak{L}}_{11}}}}\right)=2.89$$	$$\overline{\overline{\mathfrak{I}}}\left(\overline{\overline{{\mathord{\buildrel{\lower1pt\hbox{$\scriptscriptstyle\smile$}}\over{\mathfrak{W}}}}_{{\mathfrak{L}}_{11}}}}\right)=2.89$$	$$\overline{\overline{\mathfrak{I}}}\left(\overline{\overline{{\mathord{\buildrel{\lower1pt\hbox{$\scriptscriptstyle\smile$}}\over{\mathfrak{W}}}}_{{\mathfrak{L}}_{11}}}}\right)=2.89$$
$$\overline{\overline{\mathfrak{I}}}\left(\overline{\overline{{\mathord{\buildrel{\lower1pt\hbox{$\scriptscriptstyle\smile$}}\over{\mathfrak{W}}}}_{{\mathfrak{L}}_{12}}}}\right)=2.93$$	$$\overline{\overline{\mathfrak{I}}}\left(\overline{\overline{{\mathord{\buildrel{\lower1pt\hbox{$\scriptscriptstyle\smile$}}\over{\mathfrak{W}}}}_{{\mathfrak{L}}_{12}}}}\right)=2.93$$	$$\overline{\overline{\mathfrak{I}}}\left(\overline{\overline{{\mathord{\buildrel{\lower1pt\hbox{$\scriptscriptstyle\smile$}}\over{\mathfrak{W}}}}_{{\mathfrak{L}}_{12}}}}\right)=2.93$$	$$\overline{\overline{\mathfrak{I}}}\left(\overline{\overline{{\mathord{\buildrel{\lower1pt\hbox{$\scriptscriptstyle\smile$}}\over{\mathfrak{W}}}}_{{\mathfrak{L}}_{12}}}}\right)=2.93$$	$$\overline{\overline{\mathfrak{I}}}\left(\overline{\overline{{\mathord{\buildrel{\lower1pt\hbox{$\scriptscriptstyle\smile$}}\over{\mathfrak{W}}}}_{{\mathfrak{L}}_{12}}}}\right)=2.93$$
$$\overline{\overline{\mathfrak{I}}}\left(\overline{\overline{{\mathord{\buildrel{\lower1pt\hbox{$\scriptscriptstyle\smile$}}\over{\mathfrak{W}}}}_{{\mathfrak{L}}_{13}}}}\right)=2.92$$	$$\overline{\overline{\mathfrak{I}}}\left(\overline{\overline{{\mathord{\buildrel{\lower1pt\hbox{$\scriptscriptstyle\smile$}}\over{\mathfrak{W}}}}_{{\mathfrak{L}}_{13}}}}\right)=2.92$$	$$\overline{\overline{\mathfrak{I}}}\left(\overline{\overline{{\mathord{\buildrel{\lower1pt\hbox{$\scriptscriptstyle\smile$}}\over{\mathfrak{W}}}}_{{\mathfrak{L}}_{13}}}}\right)=2.92$$	$$\overline{\overline{\mathfrak{I}}}\left(\overline{\overline{{\mathord{\buildrel{\lower1pt\hbox{$\scriptscriptstyle\smile$}}\over{\mathfrak{W}}}}_{{\mathfrak{L}}_{13}}}}\right)=2.92$$	$$\overline{\overline{\mathfrak{I}}}\left(\overline{\overline{{\mathord{\buildrel{\lower1pt\hbox{$\scriptscriptstyle\smile$}}\over{\mathfrak{W}}}}_{{\mathfrak{L}}_{13}}}}\right)=2.92$$
$$\overline{\overline{\mathfrak{I}}}\left(\overline{\overline{{\mathord{\buildrel{\lower1pt\hbox{$\scriptscriptstyle\smile$}}\over{\mathfrak{W}}}}_{{\mathfrak{L}}_{14}}}}\right)=2.88$$	$$\overline{\overline{\mathfrak{I}}}\left(\overline{\overline{{\mathord{\buildrel{\lower1pt\hbox{$\scriptscriptstyle\smile$}}\over{\mathfrak{W}}}}_{{\mathfrak{L}}_{14}}}}\right)=2.88$$	$$\overline{\overline{\mathfrak{I}}}\left(\overline{\overline{{\mathord{\buildrel{\lower1pt\hbox{$\scriptscriptstyle\smile$}}\over{\mathfrak{W}}}}_{{\mathfrak{L}}_{14}}}}\right)=2.88$$	$$\overline{\overline{\mathfrak{I}}}\left(\overline{\overline{{\mathord{\buildrel{\lower1pt\hbox{$\scriptscriptstyle\smile$}}\over{\mathfrak{W}}}}_{{\mathfrak{L}}_{14}}}}\right)=2.88$$	$$\overline{\overline{\mathfrak{I}}}\left(\overline{\overline{{\mathord{\buildrel{\lower1pt\hbox{$\scriptscriptstyle\smile$}}\over{\mathfrak{W}}}}_{{\mathfrak{L}}_{14}}}}\right)=2.88$$

Then, we can calculate the values of $$\overline{\overline{{\Xi }_{\tau j}}}$$ by $$\overline{\overline{{\Xi }_{\tau j}}}=\frac{\left(1+\overline{\overline{\mathfrak{I}}}\left(\overline{\overline{{\mathord{\buildrel{\lower1pt\hbox{$\scriptscriptstyle\smile$}}\over{\mathfrak{W}}}}_{{\mathfrak{L}}_{\tau j}}}}\right)\right)}{\sum_{j=1}^{m}\left(1+\overline{\overline{\mathfrak{I}}}\left(\overline{\overline{{\mathord{\buildrel{\lower1pt\hbox{$\scriptscriptstyle\smile$}}\over{\mathfrak{W}}}}_{{\mathfrak{L}}_{\tau j}}}}\right)\right)}$$ (from Eq. (10), and the results are shown in Table 5.

**Table 5:** The values of $\overline{\overline{{\Xi }_{\tau j}}}$

$$\overline{\overline{{\mathord{\buildrel{\lower1pt\hbox{$\scriptscriptstyle\smile$}}\over{\mathfrak{W}}}}_{{\mathfrak{L}}_{1}}}}$$	$$\overline{\overline{{\mathord{\buildrel{\lower1pt\hbox{$\scriptscriptstyle\smile$}}\over{\mathfrak{W}}}}_{{\mathfrak{L}}_{2}}}}$$	$$\overline{\overline{{\mathord{\buildrel{\lower1pt\hbox{$\scriptscriptstyle\smile$}}\over{\mathfrak{W}}}}_{{\mathfrak{L}}_{3}}}}$$	$$\overline{\overline{{\mathord{\buildrel{\lower1pt\hbox{$\scriptscriptstyle\smile$}}\over{\mathfrak{W}}}}_{{\mathfrak{L}}_{4}}}}$$	$$\overline{\overline{{\mathord{\buildrel{\lower1pt\hbox{$\scriptscriptstyle\smile$}}\over{\mathfrak{W}}}}_{{\mathfrak{L}}_{5}}}}$$
$\overline{\overline{{\boldsymbol{\Xi }}_{11}}}=0.249$	$\overline{\overline{{\boldsymbol{\Xi }}_{11}}}=0.249$	$\overline{\overline{{\boldsymbol{\Xi }}_{11}}}=0.249$	$\overline{\overline{{\boldsymbol{\Xi }}_{11}}}=0.249$	$\overline{\overline{{\boldsymbol{\Xi }}_{11}}}=0.249$
$\overline{\overline{{\boldsymbol{\Xi }}_{12}}}=0.2516$	$\overline{\overline{{\boldsymbol{\Xi }}_{12}}}=0.2516$	$\overline{\overline{{\boldsymbol{\Xi }}_{12}}}=0.2516$	$\overline{\overline{{\boldsymbol{\Xi }}_{12}}}=0.2516$	$\overline{\overline{{\boldsymbol{\Xi }}_{12}}}=0.2516$
$\overline{\overline{{\boldsymbol{\Xi }}_{13}}}=0.251$	$\overline{\overline{{\boldsymbol{\Xi }}_{13}}}=0.251$	$\overline{\overline{{\boldsymbol{\Xi }}_{13}}}=0.251$	$\overline{\overline{{\boldsymbol{\Xi }}_{13}}}=0.251$	$\overline{\overline{{\boldsymbol{\Xi }}_{13}}}=0.251$
$\overline{\overline{{\boldsymbol{\Xi }}_{14}}}=0.2484$	$\overline{\overline{{\boldsymbol{\Xi }}_{14}}}=0.2484$	$\overline{\overline{{\boldsymbol{\Xi }}_{14}}}=0.2484$	$\overline{\overline{{\boldsymbol{\Xi }}_{14}}}=0.2484$	$\overline{\overline{{\boldsymbol{\Xi }}_{14}}}=0.2484$

***Step 2:*** Aggregate all attribute values for each alternative based on the IVA-IFAAPA operator and IVA-IFAAPG operator, and the results are shown in Table 6.

**Table 6:** The aggregated results for each alternative

Alternatives	IVA-IFAAPA Operator	IVA-IFAAPG Operator
$$\overline{\overline{{\mathord{\buildrel{\lower1pt\hbox{$\scriptscriptstyle\smile$}}\over{\mathfrak{W}}}}_{{\mathfrak{L}}_{1}}}}$$	([0.1517,0.2079],[0.3897,0.4635])	([0.6052,0.6823],[0.052,0.1])
$$\overline{\overline{{\mathord{\buildrel{\lower1pt\hbox{$\scriptscriptstyle\smile$}}\over{\mathfrak{W}}}}_{{\mathfrak{L}}_{2}}}}$$	([0.3396,0.4206],[0.3897,0.4635])	([0.8095,0.8642],[0.052,0.1])
$$\overline{\overline{{\mathord{\buildrel{\lower1pt\hbox{$\scriptscriptstyle\smile$}}\over{\mathfrak{W}}}}_{{\mathfrak{L}}_{3}}}}$$	([0.2079,0.2699],[0.6052,0.5564])	([0.6823,0.7494],[0.1517,0.2079])
$$\overline{\overline{{\mathord{\buildrel{\lower1pt\hbox{$\scriptscriptstyle\smile$}}\over{\mathfrak{W}}}}_{{\mathfrak{L}}_{4}}}}$$	([0.2699,0.3396],[0.5124,0.5182])	([0.7494,0.8095],[0.1,0.1517])
$$\overline{\overline{{\mathord{\buildrel{\lower1pt\hbox{$\scriptscriptstyle\smile$}}\over{\mathfrak{W}}}}_{{\mathfrak{L}}_{5}}}}$$	([0.1,0.2079],[0.6052,0.5564])	([0.5124,0.6823],[0.1517,0.2079])

***Step 3:*** Calculate the Score values based on Eq. (7), and the results are shown in Table 7.

**Table 7:** The score values

Alternatives	IVA-IFAAPA Operator	IVA-IFAAPG Operator
$$\overline{\overline{{\mathord{\buildrel{\lower1pt\hbox{$\scriptscriptstyle\smile$}}\over{\mathfrak{W}}}}_{{\mathfrak{L}}_{1}}}}$$	$$-0.2468$$	$$0.5678$$
$$\overline{\overline{{\mathord{\buildrel{\lower1pt\hbox{$\scriptscriptstyle\smile$}}\over{\mathfrak{W}}}}_{{\mathfrak{L}}_{2}}}}$$	$$-0.0465$$	$$0.7609$$
$$\overline{\overline{{\mathord{\buildrel{\lower1pt\hbox{$\scriptscriptstyle\smile$}}\over{\mathfrak{W}}}}_{{\mathfrak{L}}_{3}}}}$$	$$-0.3419$$	$$0.5361$$
$$\overline{\overline{{\mathord{\buildrel{\lower1pt\hbox{$\scriptscriptstyle\smile$}}\over{\mathfrak{W}}}}_{{\mathfrak{L}}_{4}}}}$$	$$-0.2106$$	$$0.6536$$
$$\overline{\overline{{\mathord{\buildrel{\lower1pt\hbox{$\scriptscriptstyle\smile$}}\over{\mathfrak{W}}}}_{{\mathfrak{L}}_{5}}}}$$	$$-0.4269$$	$$0.4176$$

***Step 4:*** Obtain the ranking results based on the Score values and the results are shown in Table 8.

**Table 8:** The ranking results

Methods	Ranking Values
IVA-IFAAPA Operator	$$\overline{\overline{{\mathord{\buildrel{\lower1pt\hbox{$\scriptscriptstyle\smile$}}\over{\mathfrak{W}}}}_{{\mathfrak{L}}_{2}}}}>\overline{\overline{{\mathord{\buildrel{\lower1pt\hbox{$\scriptscriptstyle\smile$}}\over{\mathfrak{W}}}}_{{\mathfrak{L}}_{4}}}}>\overline{\overline{{\mathord{\buildrel{\lower1pt\hbox{$\scriptscriptstyle\smile$}}\over{\mathfrak{W}}}}_{{\mathfrak{L}}_{1}}}}>\overline{\overline{{\mathord{\buildrel{\lower1pt\hbox{$\scriptscriptstyle\smile$}}\over{\mathfrak{W}}}}_{{\mathfrak{L}}_{3}}}}>\overline{\overline{{\mathord{\buildrel{\lower1pt\hbox{$\scriptscriptstyle\smile$}}\over{\mathfrak{W}}}}_{{\mathfrak{L}}_{5}}}}$$
IVA-IFAAPG Operator	$$\overline{\overline{{\mathord{\buildrel{\lower1pt\hbox{$\scriptscriptstyle\smile$}}\over{\mathfrak{W}}}}_{{\mathfrak{L}}_{2}}}}>\overline{\overline{{\mathord{\buildrel{\lower1pt\hbox{$\scriptscriptstyle\smile$}}\over{\mathfrak{W}}}}_{{\mathfrak{L}}_{4}}}}>\overline{\overline{{\mathord{\buildrel{\lower1pt\hbox{$\scriptscriptstyle\smile$}}\over{\mathfrak{W}}}}_{{\mathfrak{L}}_{1}}}}>\overline{\overline{{\mathord{\buildrel{\lower1pt\hbox{$\scriptscriptstyle\smile$}}\over{\mathfrak{W}}}}_{{\mathfrak{L}}_{3}}}}>\overline{\overline{{\mathord{\buildrel{\lower1pt\hbox{$\scriptscriptstyle\smile$}}\over{\mathfrak{W}}}}_{{\mathfrak{L}}_{5}}}}$$

From Table 8, we can get that the best choice is $$\overline{\overline{{\mathord{\buildrel{\lower1pt\hbox{$\scriptscriptstyle\smile$}}\over{\mathfrak{W}}}}_{{\mathfrak{L}}_{2}}}}$$ based on the IVA-IFAAPA operator and IVA-IFAAPG operator, i.e., there are the same best choice from the two methods. However, there are the different ranking results because of some variations of parameters involved in the proposed operators. Furthermore, we check the supremacy and worth of the proposed methods by comparative analysis.

## Comparative Analysis

In Sect. 4, we have given the decision-making results, to show the effectiveness and superiority of the derived method, we compare the proposed operators with various prevailing operators, such as averaging AOs for IFS by Xu [23], geometric AOs for IFS by Xu and Yager [24], PA operators for IFS by Jiang et al. [25], PA operators for IFS by Xu [26], Aczel–Alsina averaging AOs for IFS by Senapati et al. [27], the geometric Aczel–Alsina AOs for IFS by Senapati et al. [28], geometric AOs for IVIFS by Wei and Wang [30], the averaging AOs for IVIFSs by Wang et al. [31], Aczel–Alsina AOs for IVIFS by Senapati et al. [32], the power AOs for IVIFS by He et al. [33]. Then, the comparison results are shown in Table 9.

**Table 9:** Comparative results for the different operators

Methods	Score values	Ranking values
Xu [23]	$Cannot \,\,process\,\, this\,\, problem$	$Cannot \,\,process \,\,this \,\,problem$
Xu and Yager [24]	$Cannot \,\,process \,\,this \,\,problem$	$Cannot \,\,process \,\,this \,\,problem$
Jiang et al. [25]	$Cannot \,\,process\,\, this \,\,problem$	$Cannot\,\, process\,\, this \,\,problem$
Xu [26]	$Cannot \,\,process\,\, this \,\,problem$	$Cannot\,\, process\,\, this \,\,problem$
Senapati et al. [27]	$Cannot \,\,process\,\, this \,\,problem$	$Cannot \,\,process\,\, this\,\, problem$
Senapati et al. [28]	$Cannot\,\, process \,\,this \,\,problem$	$Cannot\,\, process\,\, this \,\,problem$
Wei and Wang [30]	$$\mathrm{0.1998,0.4999,0.09998,0.2998},-0.0503$$	$$\overline{\overline{{\mathord{\buildrel{\lower1pt\hbox{$\scriptscriptstyle\smile$}}\over{\mathfrak{W}}}}_{{\mathfrak{L}}_{2}}}}>\overline{\overline{{\mathord{\buildrel{\lower1pt\hbox{$\scriptscriptstyle\smile$}}\over{\mathfrak{W}}}}_{{\mathfrak{L}}_{4}}}}>\overline{\overline{{\mathord{\buildrel{\lower1pt\hbox{$\scriptscriptstyle\smile$}}\over{\mathfrak{W}}}}_{{\mathfrak{L}}_{1}}}}>\overline{\overline{{\mathord{\buildrel{\lower1pt\hbox{$\scriptscriptstyle\smile$}}\over{\mathfrak{W}}}}_{{\mathfrak{L}}_{3}}}}>\overline{\overline{{\mathord{\buildrel{\lower1pt\hbox{$\scriptscriptstyle\smile$}}\over{\mathfrak{W}}}}_{{\mathfrak{L}}_{5}}}}$$
Wang et al. [31]	$$\mathrm{0.2004,0.5005,0.1002,0.3003},-0.0498$$	$$\overline{\overline{{\mathord{\buildrel{\lower1pt\hbox{$\scriptscriptstyle\smile$}}\over{\mathfrak{W}}}}_{{\mathfrak{L}}_{2}}}}>\overline{\overline{{\mathord{\buildrel{\lower1pt\hbox{$\scriptscriptstyle\smile$}}\over{\mathfrak{W}}}}_{{\mathfrak{L}}_{4}}}}>\overline{\overline{{\mathord{\buildrel{\lower1pt\hbox{$\scriptscriptstyle\smile$}}\over{\mathfrak{W}}}}_{{\mathfrak{L}}_{1}}}}>\overline{\overline{{\mathord{\buildrel{\lower1pt\hbox{$\scriptscriptstyle\smile$}}\over{\mathfrak{W}}}}_{{\mathfrak{L}}_{3}}}}>\overline{\overline{{\mathord{\buildrel{\lower1pt\hbox{$\scriptscriptstyle\smile$}}\over{\mathfrak{W}}}}_{{\mathfrak{L}}_{5}}}}$$
Senapati et al. [32]	$$-0.2749,-0.0734,-0.4057,-0.2554,-0.491$$	$$\overline{\overline{{\mathord{\buildrel{\lower1pt\hbox{$\scriptscriptstyle\smile$}}\over{\mathfrak{W}}}}_{{\mathfrak{L}}_{2}}}}>\overline{\overline{{\mathord{\buildrel{\lower1pt\hbox{$\scriptscriptstyle\smile$}}\over{\mathfrak{W}}}}_{{\mathfrak{L}}_{4}}}}>\overline{\overline{{\mathord{\buildrel{\lower1pt\hbox{$\scriptscriptstyle\smile$}}\over{\mathfrak{W}}}}_{{\mathfrak{L}}_{1}}}}>\overline{\overline{{\mathord{\buildrel{\lower1pt\hbox{$\scriptscriptstyle\smile$}}\over{\mathfrak{W}}}}_{{\mathfrak{L}}_{3}}}}>\overline{\overline{{\mathord{\buildrel{\lower1pt\hbox{$\scriptscriptstyle\smile$}}\over{\mathfrak{W}}}}_{{\mathfrak{L}}_{5}}}}$$
He et al. [33]	$$\mathrm{0.2005,0.5006,0.1003,0.3004},-0.0497$$	$$\overline{\overline{{\mathord{\buildrel{\lower1pt\hbox{$\scriptscriptstyle\smile$}}\over{\mathfrak{W}}}}_{{\mathfrak{L}}_{2}}}}>\overline{\overline{{\mathord{\buildrel{\lower1pt\hbox{$\scriptscriptstyle\smile$}}\over{\mathfrak{W}}}}_{{\mathfrak{L}}_{4}}}}>\overline{\overline{{\mathord{\buildrel{\lower1pt\hbox{$\scriptscriptstyle\smile$}}\over{\mathfrak{W}}}}_{{\mathfrak{L}}_{1}}}}>\overline{\overline{{\mathord{\buildrel{\lower1pt\hbox{$\scriptscriptstyle\smile$}}\over{\mathfrak{W}}}}_{{\mathfrak{L}}_{3}}}}>\overline{\overline{{\mathord{\buildrel{\lower1pt\hbox{$\scriptscriptstyle\smile$}}\over{\mathfrak{W}}}}_{{\mathfrak{L}}_{5}}}}$$
IVA-IFAAPA Operator	$$-0.2468,-0.0465,-0.3419,-0.2106,-0.4269$$	$$\overline{\overline{{\mathord{\buildrel{\lower1pt\hbox{$\scriptscriptstyle\smile$}}\over{\mathfrak{W}}}}_{{\mathfrak{L}}_{2}}}}>\overline{\overline{{\mathord{\buildrel{\lower1pt\hbox{$\scriptscriptstyle\smile$}}\over{\mathfrak{W}}}}_{{\mathfrak{L}}_{4}}}}>\overline{\overline{{\mathord{\buildrel{\lower1pt\hbox{$\scriptscriptstyle\smile$}}\over{\mathfrak{W}}}}_{{\mathfrak{L}}_{1}}}}>\overline{\overline{{\mathord{\buildrel{\lower1pt\hbox{$\scriptscriptstyle\smile$}}\over{\mathfrak{W}}}}_{{\mathfrak{L}}_{3}}}}>\overline{\overline{{\mathord{\buildrel{\lower1pt\hbox{$\scriptscriptstyle\smile$}}\over{\mathfrak{W}}}}_{{\mathfrak{L}}_{5}}}}$$
IVA-IFAAPG Operator	$$\mathrm{0.5678,0.7609,0.5361,0.6536,0.4176}$$	$$\overline{\overline{{\mathord{\buildrel{\lower1pt\hbox{$\scriptscriptstyle\smile$}}\over{\mathfrak{W}}}}_{{\mathfrak{L}}_{2}}}}>\overline{\overline{{\mathord{\buildrel{\lower1pt\hbox{$\scriptscriptstyle\smile$}}\over{\mathfrak{W}}}}_{{\mathfrak{L}}_{4}}}}>\overline{\overline{{\mathord{\buildrel{\lower1pt\hbox{$\scriptscriptstyle\smile$}}\over{\mathfrak{W}}}}_{{\mathfrak{L}}_{1}}}}>\overline{\overline{{\mathord{\buildrel{\lower1pt\hbox{$\scriptscriptstyle\smile$}}\over{\mathfrak{W}}}}_{{\mathfrak{L}}_{3}}}}>\overline{\overline{{\mathord{\buildrel{\lower1pt\hbox{$\scriptscriptstyle\smile$}}\over{\mathfrak{W}}}}_{{\mathfrak{L}}_{5}}}}$$

From Table 9, we can obtain that the finest optimal is $$\overline{\overline{{\mathord{\buildrel{\lower1pt\hbox{$\scriptscriptstyle\smile$}}\over{\mathfrak{W}}}}_{{\mathfrak{L}}_{2}}}}$$ for all methods.

1. Wang et al. [31] proposed the averaging AOs for IVA-IFS, then we can get the ranking result $$\overline{\overline{{\mathord{\buildrel{\lower1pt\hbox{$\scriptscriptstyle\smile$}}\over{\mathfrak{W}}}}_{{\mathfrak{L}}_{2}}}}>\overline{\overline{{\mathord{\buildrel{\lower1pt\hbox{$\scriptscriptstyle\smile$}}\over{\mathfrak{W}}}}_{{\mathfrak{L}}_{4}}}}>\overline{\overline{{\mathord{\buildrel{\lower1pt\hbox{$\scriptscriptstyle\smile$}}\over{\mathfrak{W}}}}_{{\mathfrak{L}}_{1}}}}>\overline{\overline{{\mathord{\buildrel{\lower1pt\hbox{$\scriptscriptstyle\smile$}}\over{\mathfrak{W}}}}_{{\mathfrak{L}}_{3}}}}>\overline{\overline{{\mathord{\buildrel{\lower1pt\hbox{$\scriptscriptstyle\smile$}}\over{\mathfrak{W}}}}_{{\mathfrak{L}}_{5}}}}$$. Although the best choice is the same as our method, the ranking result is slightly different. The reason is that our method considers the power relation between two attributes. Wang et al. method [31] used only the Arithmetic averaging operator and the operational laws based on the algebraic t-norm and t-conorm, our method is more generalized than it.
2. Wei and Wang [30] proposed the geometric AOs for IVA-IFS, then we can get the ranking result $$\overline{\overline{{\mathord{\buildrel{\lower1pt\hbox{$\scriptscriptstyle\smile$}}\over{\mathfrak{W}}}}_{{\mathfrak{L}}_{2}}}}>\overline{\overline{{\mathord{\buildrel{\lower1pt\hbox{$\scriptscriptstyle\smile$}}\over{\mathfrak{W}}}}_{{\mathfrak{L}}_{4}}}}>\overline{\overline{{\mathord{\buildrel{\lower1pt\hbox{$\scriptscriptstyle\smile$}}\over{\mathfrak{W}}}}_{{\mathfrak{L}}_{1}}}}>\overline{\overline{{\mathord{\buildrel{\lower1pt\hbox{$\scriptscriptstyle\smile$}}\over{\mathfrak{W}}}}_{{\mathfrak{L}}_{3}}}}>\overline{\overline{{\mathord{\buildrel{\lower1pt\hbox{$\scriptscriptstyle\smile$}}\over{\mathfrak{W}}}}_{{\mathfrak{L}}_{5}}}}$$. Similarly, the best choice is the same as our method, the ranking result is slightly different. The reason is that our method considers the power relation between two attributes. Wei and Wang method [30] used only the geometric operator and the operational laws based on the algebraic t-norm and t-conorm, our method is more generalized than it.
3. He et al. [33] proposed the PA operators for IVA-IFS, then we can get the ranking result $$\overline{\overline{{\mathord{\buildrel{\lower1pt\hbox{$\scriptscriptstyle\smile$}}\over{\mathfrak{W}}}}_{{\mathfrak{L}}_{2}}}}>\overline{\overline{{\mathord{\buildrel{\lower1pt\hbox{$\scriptscriptstyle\smile$}}\over{\mathfrak{W}}}}_{{\mathfrak{L}}_{4}}}}>\overline{\overline{{\mathord{\buildrel{\lower1pt\hbox{$\scriptscriptstyle\smile$}}\over{\mathfrak{W}}}}_{{\mathfrak{L}}_{1}}}}>\overline{\overline{{\mathord{\buildrel{\lower1pt\hbox{$\scriptscriptstyle\smile$}}\over{\mathfrak{W}}}}_{{\mathfrak{L}}_{3}}}}>\overline{\overline{{\mathord{\buildrel{\lower1pt\hbox{$\scriptscriptstyle\smile$}}\over{\mathfrak{W}}}}_{{\mathfrak{L}}_{5}}}}.$$ This ranking result is the same as our method based on IVA-IFAAPA Operator. He et al.’ method [33] used only the operational laws based on the algebraic t-norm and t-conorm, our method is more generalized than it.
4. Senapati et al. [32] proposed the Aczel–Alsina averaging AOs for IVA-IFS, then we can get the ranking result $$\overline{\overline{{\mathord{\buildrel{\lower1pt\hbox{$\scriptscriptstyle\smile$}}\over{\mathfrak{W}}}}_{{\mathfrak{L}}_{2}}}}>\overline{\overline{{\mathord{\buildrel{\lower1pt\hbox{$\scriptscriptstyle\smile$}}\over{\mathfrak{W}}}}_{{\mathfrak{L}}_{4}}}}>\overline{\overline{{\mathord{\buildrel{\lower1pt\hbox{$\scriptscriptstyle\smile$}}\over{\mathfrak{W}}}}_{{\mathfrak{L}}_{1}}}}>\overline{\overline{{\mathord{\buildrel{\lower1pt\hbox{$\scriptscriptstyle\smile$}}\over{\mathfrak{W}}}}_{{\mathfrak{L}}_{3}}}}>\overline{\overline{{\mathord{\buildrel{\lower1pt\hbox{$\scriptscriptstyle\smile$}}\over{\mathfrak{W}}}}_{{\mathfrak{L}}_{5}}}}.$$ Although the best choice is the same as our method, the ranking result is different from the other methods. The reason is that our method considers the power relation between any two attributes. Since the ranking result is different from the other methods, its effectiveness is questionable. Senapati et al.’ method [32] used only the operational laws based on the Aczel–Alsina t-norm and t-conorm, our method is more generalized than it.

From the above analysis, we can see that all methods produced the same best choice for this example, however, the ranking results are different, because our method adopted the PA operators and the operational laws based on the Aczel–Alsina t-norm and t-conorm, it is more general than the others. In addition, we explained the novelty and supremacy of the proposed methods by comparative analysis with some existing methods shown in Table 10.

**Table 10:** Theoretical analysis of the proposed work

Methods	Truth grade	Falsity grade	Condition	Interval-valued information
Fuzzy sets	$$\surd$$	$$\times$$	$$0\le \overline{\overline{{\zeta }_{\overline{\overline{{\mathord{\buildrel{\lower1pt\hbox{$\scriptscriptstyle\smile$}}\over{\mathfrak{W}}}}_{\mathfrak{L}}}}}}}\left(\widetilde{{\mu }_{\varrho }}\right)\le 1$$	$$\times$$
Intuitionistic fuzzy sets	$$\surd$$	$$\surd$$	$$0\le \overline{\overline{{\zeta }_{\overline{\overline{{\mathord{\buildrel{\lower1pt\hbox{$\scriptscriptstyle\smile$}}\over{\mathfrak{W}}}}_{\mathfrak{L}}}}}}}\left(\widetilde{{\mu }_{\varrho }}\right)+\overline{\overline{{\eta }_{\overline{\overline{{\mathord{\buildrel{\lower1pt\hbox{$\scriptscriptstyle\smile$}}\over{\mathfrak{W}}}}_{\mathfrak{L}}}}}}}\left(\widetilde{{\mu }_{\varrho }}\right)\le 1$$	$$\times$$
Interval-valued intuitionistic fuzzy set	$$\surd$$	$$\surd$$	$$0\le {\overline{\overline{{\zeta }_{\overline{\overline{{\mathord{\buildrel{\lower1pt\hbox{$\scriptscriptstyle\smile$}}\over{\mathfrak{W}}}}_{\mathfrak{L}}}}}}}}^{u}\left(\widetilde{{\mu }_{\varrho }}\right)+{\overline{\overline{{\eta }_{\overline{\overline{{\mathord{\buildrel{\lower1pt\hbox{$\scriptscriptstyle\smile$}}\over{\mathfrak{W}}}}_{\mathfrak{L}}}}}}}}^{u}\left(\widetilde{{\mu }_{\varrho }}\right)\le 1$$	$$\surd$$

In a word, because the proposed methods are developed based on Aczel–Alsina operators with power aggregation operators for IVA-IFSs, they are the generalized form of some existing works such as the averaging AOs for IFS from Xu [23], the geometric AOs for IFS from Xu and Yager [24], the PA operators for IFS from Jiang et al. [25] and Xu [26], the Aczel–Alsina averaging AOs for IFS and the geometric Aczel–Alsina AOs for IFS from Senapati et al. [27] and Senapati et al. [28], Geometric AOs for IVIFS by Wei and Wang [30], the averaging AOs for IVIFSs by Wang et al. [31], Aczel–Alsina AOs for IVIFS by Senapati et al. [32], and the power AOs for IVIFS by He et al. [33]. We can explain them as follows:

1. If we only used the PA operators based on algebraic laws, then our proposed operators are reduced to ones by He et al. [33].
2. If we only used the simple averaging and geometric aggregation operators based on Aczel–Alsina operational laws, then our proposed operators are reduced to ones by Senapati et al. [32].
3. If we only used the simple averaging and geometric aggregation operators based on algebraic operational laws, then our proposed operators are reduced to ones by Wei and Wang [30] and Wang et al. [31].

Of course, because the proposed methods can only deal only with one-dimension information, they cannot process the phase term, obviously, there is shortcoming in this study, and it is further study on the Aczel–Alsina power aggregation operators based on complex IVA-IFSs.

## Conclusion

The operational laws based on the Aczel–Alsina t-norm and t-conorm are more general than some existing operations, the PA operators can consider the power relation between any two attributes, and IVA-IFS can better express the uncertain information, in this paper, we fully consider their advantages, and proposed some PA operators based on the operational laws from the Aczel–Alsina t-norm and t-conorm. The main contributions are shown as follows.

1. Proposed the IVA-IFAAPA, IVA-IFAAPOA, IVA-IFAAPG, and IVA-IFAAPOG operators.
2. Discussed the properties of the presented operators such as idempotency, monotonicity, and boundedness.
3. Developed a MADM method with IVA-IF information.
4. Demonstrated the effectiveness and superiority of the derived method by comparing it with some existing operators.

In the future, we will modify the Aczel–Alsina PA operators based on the IVA-IF set. Furthermore, we also utilize the proposed operators and method in the fields of decision-making analysis [34], artificial intelligence, neural networks, pattern recognition, clustering analysis, and medical diagnosis.

## Data Availability

Not applicable.

## Abbreviations

PA: Power aggregation
A-IFS: Atanassov-intuitionistic fuzzy set
IVA-IFS: Interval-valued Atanassov-intuitionistic fuzzy set
IVA-IFAAPA: Interval-valued Atanassov-intuitionistic fuzzy Aczel–Alsina power averaging
IVA-IFAAPOA: Interval-valued Atanassov-intuitionistic fuzzy Aczel–Alsina power ordered averaging
IVA-IFAAPG: Interval-valued Atanassov-intuitionistic fuzzy Aczel–Alsina power geometric
IVA-IFAAPOG: Interval-valued Atanassov-intuitionistic fuzzy Aczel–Alsina power ordered geometric
MADM: Multi-attribute decision-making
FS: Fuzzy sets
IFS: Intuitionistic fuzzy sets
IVIFS: Interval-valued intuitionistic fuzzy sets
AOs: Aggregation operators
